# Linking local and large-scale salient events with oscillatory and broadband arrhythmic activities in the resting human brain

**DOI:** 10.1162/IMAG.a.1193

**Published:** 2026-04-03

**Authors:** Damián Dellavale, Emahnuel Troisi Lopez, Antonella Romano, Giovanni Rabuffo, Pierpaolo Sorrentino

**Affiliations:** Consejo Nacional de Investigaciones Científicas y Técnicas (CONICET), Centro Atómico Bariloche, San Carlos de Bariloche, Río Negro, Argentina; Global Brain Health Institute (GBHI), Trinity College Dublin (TCD), Dublin, Ireland; Institute of Applied Sciences and Intelligent Systems, National Research Council (CNR), Pozzuoli, Italy; University of Naples Parthenope, Department of Motor Science and Wellness, Naples, Italy; Center for Brain and Cognition, Universitat Pompeu Fabra, Barcelona, Spain; Institut de Neurosciences des Systèmes (INS), INSERM, Aix-Marseille Universitè, Marseille, France; Università degli Studi di Napoli Parthenope, Dipartimento delle Scienze Mediche, Motorie e del Benessere, Via Ammiraglio Ferdinando Acton, Napoli, Italy

**Keywords:** human MEG, large-scale salient events, brain oscillations, broadband arrhythmic activity, group delay, complex baseband representation

## Abstract

Narrowband oscillations (NOs) and broadband arrhythmic activity (BAA) are valuable conceptualizations extensively used to interpret brain data, with NOs linked to communication and synchronization and BAA encompassing scale-free dynamics and neuronal avalanches. Although both frameworks offer critical insights into brain function, they have largely evolved in parallel, with limited integration and no unifying mechanistic account of how these dynamics interact to generate transient, salient events (SEs). This gap is particularly pressing given recent interest in how SEs—brief (≈100 ms) bursts of activity coordinated across brain regions—relate to large-scale brain function and cognition. To address this, we introduce a signal-level framework that links the Fourier spectral properties (oscillation-domain) of neural signals to the emergence of realistic SEs in the time-domain from NOs and BAA. Our approach is grounded in a novel concept—spectral group delay consistency (SGDC)—along with associated measures that quantify the temporal alignment of spectral components and capture the conditions under which NOs and BAA coalesce into transient, burst-like events. Unlike traditional power- or phase-based measures, or higher-order statistical metrics such as kurtosis and cokurtosis, SGDC provides a signal-level mechanistic account of how local and large-scale SEs emerge from the spectral structure of the underlying signals. Empirical validation is provided using source-reconstructed MEG data from a large cohort and a comprehensive array of features characterizing the statistical, spatiotemporal, and spectral properties of observed SEs. We found that the SEs identified in our empirical MEG dataset can be segregated based on their spectral signature in two main groups having different propagation patterns. Using generative models based on the SGDC mechanism, we provide a theoretical framework to interpret these experimental results showing that cluster 2 events are specifically related to the long-range spread of narrowband alpha bursts across the brain network (i.e., SNEs: salient network events), whereas cluster 1 events correspond to more short-lived and spatially localized fluctuations mainly promoted by the BAA (i.e., SLEs: salient local events). We also provide analytical arguments and numerical simulations showing that (a) high SGDC in specific narrow frequency bands, (b) transient cross-regional coherent Nos, and (c) BAA are all key ingredients for the emergence of realistic SNEs.

## 1 Introduction

The human brain generates complex behaviors from the coordinated interaction of neuronal populations, with evidence showing different degrees of specialization/distribution of these networks. Such coordination is accompanied (or driven) by neural activity patterns that can be measured using techniques such as electroencephalography (EEG) or magnetoencephalography (MEG). In general, electromagnetic brain signals are characterized by both narrowband rhythmic (i.e., oscillations) and broadband arrhythmic (e.g., 1/ f scaling in power spectra) components (He, 2014).

Oscillatory neural activity comprises rhythmic, periodic fluctuations around a central value in the brain’s signals, which occur across various narrow frequency bands and have been associated with specific cognitive functions (Buzsáki, 2006). For example, the alpha rhythm, typically between 8 and 13 Hz, emerges during eyes-closed wakefulness (Adrian & Matthews, 1934; Berger, 1929; Palva & Palva, 2007), while the gamma rhythm, exceeding 30 Hz, has been proposed to play a role in higher cognitive processes (Bosman et al., 2014). These oscillatory components manifest as “bumps” in the signals’ power spectral density (PSD). In contrast to brain oscillations, broadband arrhythmic neural activity exhibits a more complex and irregular nature, often associated with scale-free dynamics (i.e., no characteristic temporal scale) (He, 2014). It generally, but not exclusively, displays a $1/\;f^{\beta }$ decay pattern in the PSD featuring a fractal-like distribution of power across frequencies, with β spanning a range of values depending on the brain condition, frequency range, and recording modality (roughly 0.1≲β≲5 ). This broadband activity contributes significantly to the brain’s overall signal and is intricately linked with cognitive processes, potentially carrying valuable information (Nanda et al., 2023). Traditionally, the study of brain *Narrowband Oscillations* (NOs) and *Broadband Arrhythmic Activity* (BAA) has provided two lenses through which electrophysiological data have been examined (Gerster et al., 2022). In general, spectral (i.e., oscillation-domain) attributes such as power and phase offer rich insights into brain dynamics, enabling the discrimination of brain activity during perceptual tasks and distinguishing between healthy and pathological dynamics in resting states (Keitel et al., 2022). For instance, the literature on brain connectivity has traditionally diverged into two primary streams: one emphasizing NOs—rhythmic, frequency-specific activity linked to communication and synchronization (Brookes et al., 2011; de Pasquale et al., 2010; Hipp et al., 2012; Marzetti et al., 2013; Schoffelen & Gross, 2009)—and another focused on BAA, encompassing scale-free dynamics such as 1/ f activity and neuronal avalanches (Becker et al., 2018; Pauls et al., 2024; Wen & Liu, 2016). Although both frameworks offer critical insights into brain function, they have largely evolved in parallel, with limited integration and no unifying mechanistic account of how these dynamics interact to generate transient, *Salient Events* (SEs). This gap is particularly pressing given recent interest in how SEs—brief (≈100 ms) bursts of activity coordinated across brain region—relate to large-scale brain function and cognition (Arviv et al., 2019; Lombardi et al., 2014; Lombardi et al., 2023a, 2023b; Miller et al., 2019; Palva et al., 2013; Plenz, 2014; van Ede et al., 2018).

Besides NOs and BAA, the analysis of collective brain dynamics reveals that system-level neuronal activity is interspersed by two types of SEs: *Salient Local Events* (SLEs) and *Salient Network Events* (SNEs). During SNEs, subsets of brain regions collectively exhibit rare fluctuations above a threshold (e.g., signal amplitude >3 standard deviations), igniting from specific brain sites, propagating across the brain circuitry in an avalanche-like cascade of activations, and finally decaying below the threshold. As an example, Figure C.1 in Supplementary Material C shows an SE observed in our MEG dataset, constituted by transient above-threshold fluctuations overlapped (i.e., disclosing time-overlap, coordinated) across five brain regions. As the SE shown in Supplementary Figure C.1 involves the activation of more than one brain region, it is referred to as a Salient Network Event (SNE). However, a local transient above-threshold fluctuation involving the activation of just one brain region is named *Salient Local Event* (SLE). SNEs occur aperiodically and are consistently observed across imaging modalities, including multielectrode array recordings (Beggs & Plenz, 2003, 2004), EEG (Duma et al., 2023; Palva et al., 2013), MEG (Palva et al., 2013; Shriki et al., 2013), SEEG (Priesemann et al., 2013; Zhigalov et al., 2015), fMRI (Tagliazucchi et al., 2012), and calcium imaging (Bowen et al., 2019; Yaghoubi et al., 2018). In particular, SNEs have drawn considerable interest due to their potential significance in information processing (Shew & Plenz, 2013; Shew et al., 2011), facilitating responses with a wide dynamical range (Kinouchi & Copelli, 2006; Larremore et al., 2011), and playing a role in achieving flexible dynamics (Sorrentino et al., 2021a, 2022; Tognoli & Kelso, 2014). A specific subtype of SNEs is known as *neuronal avalanches*. The latter were largely studied in the context of the *critical brain hypothesis*, which posits that the brain might be operating near a critical point (i.e., at the edge of a phase transition). In fact, neuronal avalanches display hallmark properties expected in systems that self-organize at a critical point, such as the power-law distribution of avalanche durations (life span) and sizes (number of regions recruited) (Moretti & Muñoz, 2013; Palva et al., 2013; Shriki et al., 2013). However, previous studies raised concerns about the interpretation of power-law statistics associated with neuronal avalanches. First, power-law distributed avalanches have been found in stochastic noncritical systems (see Chan et al., 2025 and references therein). These works highlight the fact that power-law distributions are not unique to systems near a critical point or a phase transition and can be generated by other mechanisms (Newman, 2005). Second, several factors can contribute to deviations from power-law statistics such as finite size effects (size of the neuronal network or the sampling region) and thresholding procedures used for avalanche detection (Touboul & Destexhe, 2010, 2017). Third and more crucially, the neuronal avalanches statistics can be influenced by heterogeneous factors such as network interaction/synchronization, the concomitant presence of oscillations, and/or other type of SEs (e.g., IEDs: interictal epileptiform discharges) and also external interventions (e.g., antiseizure medications) (Muller et al., 2025).

The signal processing tools proposed in this work can be used to study a variety of SLEs and SNEs: sleep spindles and K-complexes observed during non-rapid-eye-movement sleep, IEDs, and spike and wave discharges (SWDs) associated with epileptogenicity (Dellavale et al., 2023) and paroxysmal slow-wave events (PSWEs) observed in epilepsy and age-related neuropathology (e.g., Alzheimer’s disease) (Milikovsky et al., 2019; Power et al., 2024). In general, these SEs do not follow power-law statistics, indeed, IEDs, SWDs, and PSWEs have been observed in a wide range of dynamical regimes associated with clinical and subclinical brain states (see for instance fig. 5 in Muller et al. (2025)). Thus, for the sake of generality, we focus our analysis on the relationship among NOs, BAA, and SEs, without implying a connection to power-law distributed neuronal avalanches nor the brain criticality hypothesis.

NOs, BAA, and SEs offer valuable conceptualizations to interpret brain data; however, these well-established perspectives have mainly progressed in parallel, with only limited literature linking them largely restricted to the context of neuronal avalanches (Arviv et al., 2019; Lombardi et al., 2014; Lombardi et al., 2023a, 2023b; Miller et al., 2019; Palva et al., 2013; Plenz, 2014). Given the ubiquitous and concurrent presence of NOs, BAA, and SEs in the brain during rest, a fundamental question arises: Can we establish a connection between these perspectives? In other words, can we invoke a parsimonious explanation that justifies the simultaneous presence of these phenomena? To address this, we introduce a signal-level framework that links the Fourier spectral properties (oscillation-domain) of neural signals to the emergence of realistic SEs in the time-domain from rhythmic and broadband aperiodic dynamics. Our approach is grounded in a novel concept—spectral group delay consistency (SGDC)—along with associated measures that quantify the temporal alignment of spectral components and capture the conditions under which NOs and BAA coalesce into transient, burst-like events. Unlike traditional power- or phase-based measures, or higher-order statistical metrics such as kurtosis and cokurtosis, SGDC provides a signal-level mechanistic account of how local (SLEs) and large-scale (SNEs) salient events emerge from the spectral structure of the underlying signals.

While previous works primarily focused on describing the *interaction* between neuronal avalanches and NOs, in this work we adopt a bottom-up approach, using generative models based on the SGDC mechanism, aimed at elucidating how local and large-scale SEs *emerge* from the oscillatory and broadband arrhythmic components of the brain activity. The proposed data analysis tools are supported by a signal-level analytical SGDC framework designed to be applicable across a variety of (bio)physical domains, regardless of the specific details of the underlying system.

In addition, empirical validation is provided using source-reconstructed MEG data from a large cohort, demonstrating that transient phase-structured alpha bursts, shaped by the SGDC mechanism, contribute to long-range coordination during rest. This extends the communication-through-coherence (CTC) hypothesis, according to which neuronal information is transferred via phase alignment (coherence) of rhythmic activity (Fries, 2005, 2015), to the transient domain. Additionally, SGDC links to findings that NOs interact with fast microstates (≈100–200 ms) (Baker et al., 2014; Becker et al., 2020; Pauls et al., 2024; Tibon & Tsvetanov, 2022) and may modulate long-range dependencies across time scales (Becker et al., 2018). Thus, while previous studies have described SEs within the framework of neuronal avalanches, they often lacked a generative, signal-level account. Here, we bridge that divide by offering a mathematically grounded and empirically validated framework that accounts for oscillatory and aperiodic bursts perspectives on brain activity.

## 2 Methods

### 2.1 Participants and data

**Fig. 1.**
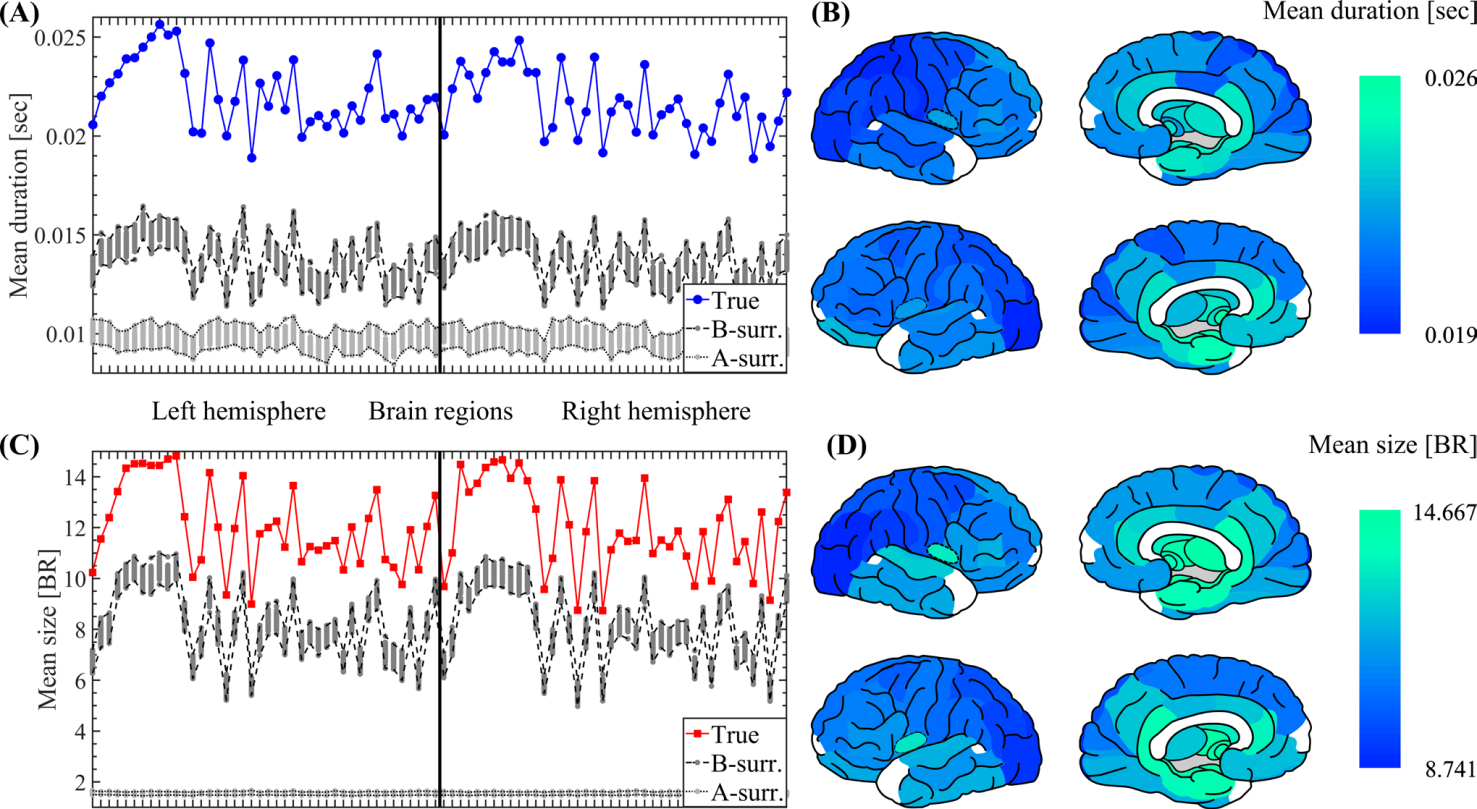
Spatiotemporal characterization of SEs. (A) Spatial profile showing the mean duration of SEs propagating through each brain region (mean value across the 47 participants, see Section 2.4 in Methods). The mean event duration is shown for the MEG data together with the 100 A- and B-surrogates (see Section 2.8 in Methods). The labels and ordering of the brain regions are the same as those shown in Supplementary Figure C.2. (B) Brain topographies for the mean duration of SEs as shown in panel A. (C) Same as in A for the size of SEs. (D) Same as in B for the size of SEs. Symbols and abbreviations: SEs, salient events; BR, brain regions.

We analyzed a source-reconstructed MEG dataset previously published in Sorrentino et al. (2021a, 2021b). In short, 58 young adults (32 males/26 females, mean age ± SD was 30.72 ± 11.58) were recruited from the general community. All participants were right-handed and native Italian speakers. The inclusion criteria were (1) no major internal, neurological, or psychiatric illnesses; and (2) no use of drugs or medication that could interfere with MEG/MRI signals. The study complied with the Declaration of Helsinki and was approved by the local ethics committee. All participants gave written informed consent. The details regarding the MRI acquisition are described in Sorrentino et al. (2021b). All technical details in connection with the MEG device are reported in Rombetto et al. (2014). MEG pre-processing and source reconstruction were performed as in Sorrentino et al. (2021a, 2021b). Briefly, the MEG registration was divided into two eyes-closed segments of 3:30 min each. To identify the position of the head, four anatomical points and four position coils were digitized. Electrocardiogram (ECG) and electro-oculogram (EOG) signals were also recorded. The MEG signals, after an anti-aliasing filter, were acquired at 1024 Hz, then a fourth-order Butterworth IIR band-pass filter in the 0.5–48 Hz band was applied. Principal component analysis was used to remove environmental noise measured by reference magnetometers. Supervised independent component analysis was adopted to clean the data from physiological artifacts, such as eye blinking (if present) and heart activity (generally one component). Noisy channels were identified and removed manually by an expert rater (136 ± 4 sensors were kept). After this pre-processing, 47 subjects were selected for this work and all further analyses were conducted on traces of 1 min in duration source reconstructed to 84 brain regions of interest (ROI) based on the Desikan–Killiany–Tourville (DKT) anatomical parcellation atlas (see brain topographies in Fig. 1 and Supplementary Fig. C.3).

### 2.2 Salient events detection

To estimate SEs, we first detected the local above-threshold fluctuations on the pre-processed and source-reconstructed MEG time series as described in Section 2.1. In each participant, the 1-min source-reconstructed MEG time series of each brain region were individually z-scored. Positive and negative excursions beyond a threshold were then identified. The amplitude threshold was set to |z|=3 , equivalent to 3 standard deviations (±3σ or equivalently |z|=3 ). The same amplitude threshold |z|=3 was used in all analyzed brain regions. This procedure was applied separately to all the 47 participants included in the study. An analysis supporting the validity and robustness of using a single amplitude threshold (|z|=3 ) consistently across all 47 participants is presented in Supplementary Material C.1 (Figures C.7 - C.9). Then the SEs duration was assessed by considering that a salient event begins when, in a sequence of contiguous time bins, at least one brain region is active (i.e., above the amplitude activation threshold: |z|>3 ) and ends when all the brain regions are inactive (i.e., below the amplitude activation threshold: |z|≤3 ) (Beggs & Plenz, 2003; Shriki et al., 2013). Besides, the SEs size was defined as the total number of brain regions activated during a given event. Note that a salient event involving more than one brain region (i.e., SNE) is associated with a sequence adjacent time bins in which at least one brain region is active (|z|>3 ). Thus, the detection of SNE depends on the time binning of the analyzed time series. Unless otherwise specified, in this study we used a time binning corresponding to one time sample per time bin (time binning = 1 ms). This procedure allowed the detection of both SLEs (i.e., SEs of size =1 brain region) and SNEs (i.e., SEs of size >1 brain region, see Supplementary Fig. C.1).

### 2.3 Salient events activation and co-activation matrices

**Fig. 2.**
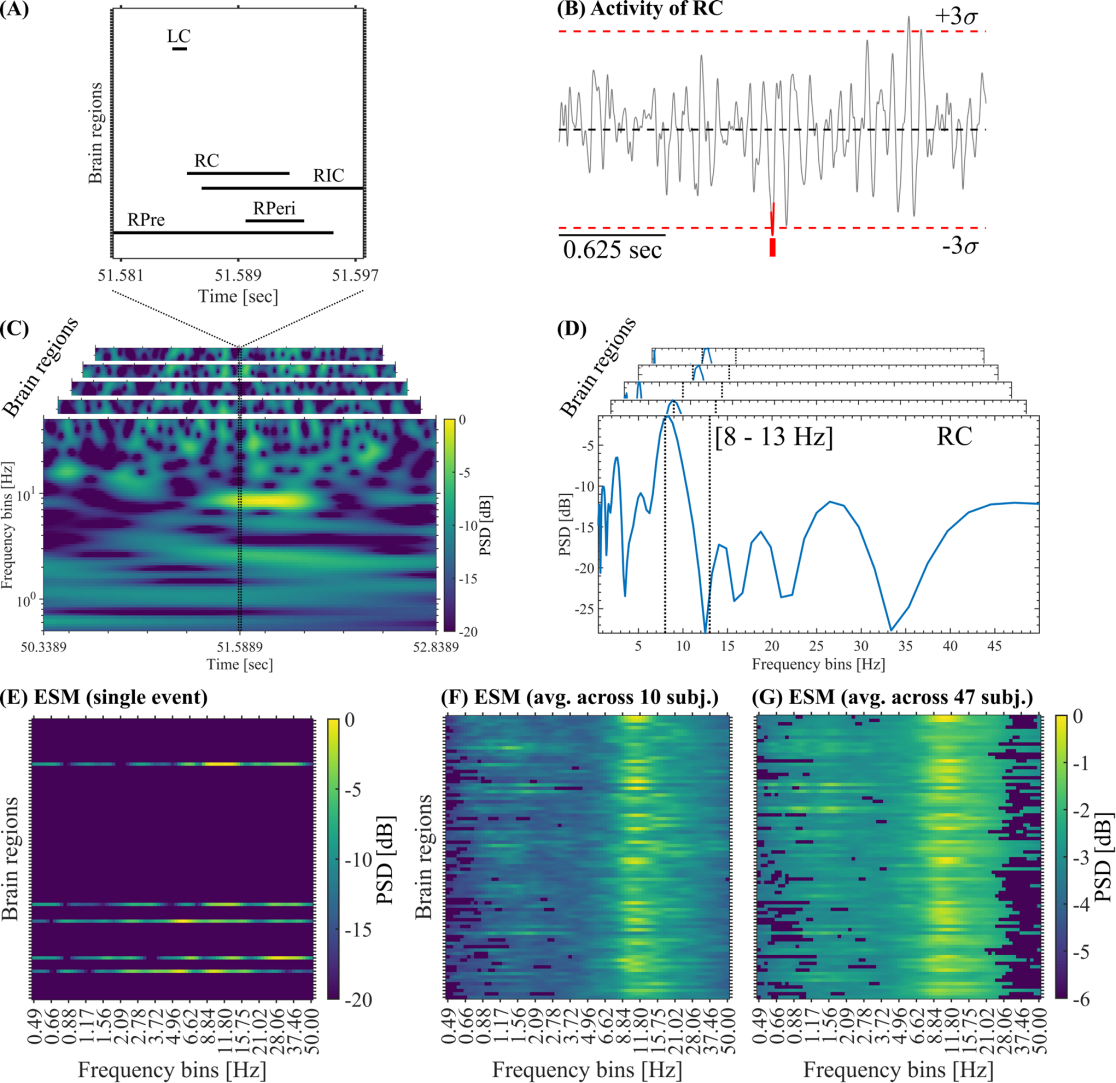
Event Spectral Matrix. (A) Activation matrix of a single SE showing the time intervals in which each brain region was active (i.e., absolute amplitude >3σ ). (B) Activity of the brain region RC disclosing the above-threshold fluctuation (highlighted in red) associated with the SE shown in panel A. (C) Whitened time–frequency maps of each brain region involved in the SE shown in panel A. (D) Whitened power spectra associated with each brain region involved in the SE shown in panel C. The vertical dotted lines indicate the alpha band. To build the ESM, we average the whitened time–frequency maps selectively across the time samples corresponding to the occurrence of each SE. As a result, we obtain a whitened power spectrum for each brain region (see Section 2.5 in Methods). (E) ESM corresponding to the SE shown in panel C. (F) Mean ESM resulting from the average across all the SEs detected in the 10 subjects, and then, Bonferroni thresholded using the C-surrogates (see Methods). (G) Mean ESM resulting from the average across all the SEs detected in the 47 subjects, and then, Bonferroni thresholded using the B-surrogates (see Methods). Symbols and abbreviations: SEs, salient events; ESM, Event Spectral Matrix; PSD, power spectral density; RPre, right precuneus; RC, right cuneus; RPeri, right pericalcarine; RIC, right isthmus cingulate; LC, left cuneus.

For each detected SE, we computed the activation matrix (brain regions × time bins) as follows. The source-reconstructed, z-scored, and time binned signal were binarized, such that, at any time bin, a brain region exceeding ±3 was set to 1 (active), and all other regions were set to 0 (inactive, see Fig. 2A, B). For each detected SE, we also computed the co-activation matrix (brain regions × brain regions) by assigning 1 to all the brain regions recruited in that particular event. Thus, the diagonal of the co-activation matrix contains 1 sec in all the brain regions active during a given SE. Besides, summation across rows (or columns) produce, in each brain region, the number of co-activated regions during a given SE (i.e., in terms of graph theory, this is known as the degree of each brain region). The mean co-activation matrix shown in Figure 7C was computed by first averaging the co-activation matrices corresponding to all the SEs detected in each subject, and then, averaging the resulting matrix across all the participants.

### 2.4 Salient events spatiotemporal profile

To characterize SEs spatiotemporal profile, we introduce two ROI-wise metrics: The *mean event duration* measuring the typical duration of SEs propagating through a brain region; and the *mean event size* measuring the typical size of SEs propagating through a brain region. Specifically, we assign to each brain region the mean event duration (or size) computed on all the SEs recruiting that particular region. The *mean event duration* and *mean event size* profiles shown in Figure 1 were computed by first considering all the SEs detected in each subject, and, then, averaging the resulting profiles across all the participants.

### 2.5 Event spectral matrix

**Fig. 3.**
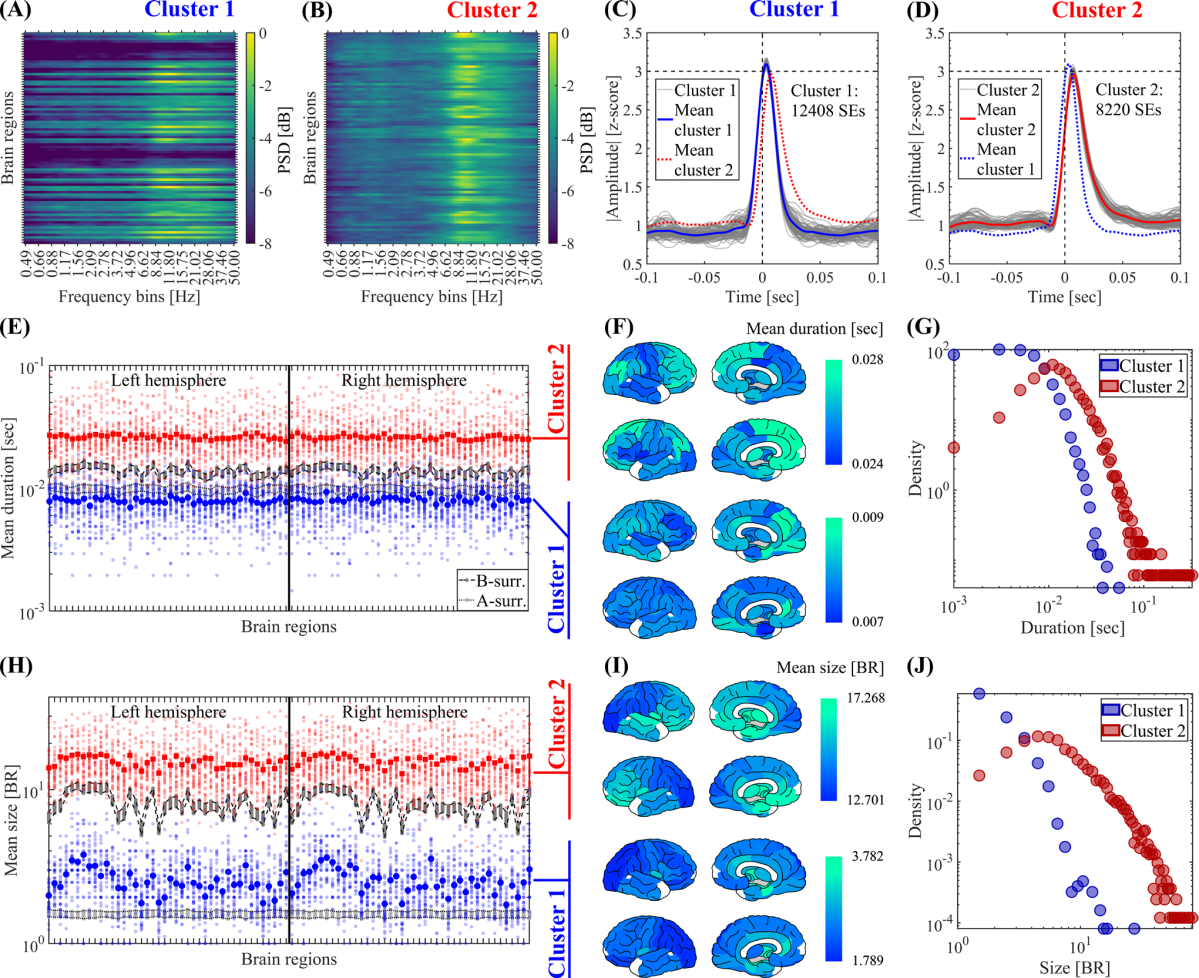
Clustering of SEs according to their spectral signature. The SEs obtained from 41 subjects were clustered using the Louvain algorithm (resolution parameter γ=1 , see Methods). (A, B) Mean ESM of the two SE clusters identified by the Louvain algorithm computed on the SEs detected in the 41 participants. (C, D) Waveform shapes of the SEs pertaining to the two SE clusters identified by the Louvain algorithm. Thin gray lines correspond to the average waveform shape in each brain region. Thick blue and red lines correspond to the resulting waveform shape averaged across the brain regions for clusters 1 and 2 SEs, respectively. (E) Spatial profile showing the mean duration of SEs pertaining to cluster 1 (in blue) and cluster 2 (in red). For the true data, the small and big markers correspond to the mean spatial profile in each patient and the average across the 41 participants, respectively (see Methods). The labels and ordering of the brain regions are the same as those shown in Supplementary Figure C.2. To test the significance of the difference of the mean SEs duration between cluster 1 and cluster 2, in each brain region, we computed a non-parametric permutation test (random sampling without replacement, $1\times 10^{4}$ permutations). All the brain regions disclosed a statistically significant difference of the mean SEs duration between clusters 1 and 2 (the Bonferroni-adjusted two-tailed P values result P<0.001 in all the brain regions). (F) Brain topographies for the mean duration of SEs averaged across the 41 participants as shown in panel E. (G) Distribution of the duration of SEs pertaining to cluster 1 and cluster 2 observed in the 41 participants. (H) Same as in E for the size of SEs. To test the significance of the difference of the mean SEs size between cluster 1 and cluster 2, in each brain region we computed a non-parametric permutation test (random sampling without replacement, $1\times 10^{4}$ permutations). All the brain regions disclosed a statistically significant difference of the mean SEs size between clusters 1 and 2 (the Bonferroni-adjusted two-tailed P values result P<0.001 in all the brain regions). (I) Same as in F for the size of SEs. (J) Same as in G for the size of SEs. Symbols and abbreviations: SEs, salient events; ESM, Event Spectral Matrix; BR, brain regions.

For the spectral characterization of SEs, we introduce the Event Spectral Matrix (ESM). To obtain the ESM we first compute the whitened time–frequency representation on the whole time series of each brain region (see Fig. 2C). Then, the time–frequency maps were selectively averaged across the time points corresponding to the occurrence of the SE of interest. As a result, we obtain a whitened power spectrum corresponding to each brain region recruited by that particular SE (see Fig. 2D). Finally, these power spectra are arranged in a single matrix conforming the ESM (Brain regions × Frequency bins, see Fig. 2F). The time–frequency maps were computed as scalograms using Morlet wavelets of duration 2gwidth/(2πf) sec., where g=3 (std. dev.), width=7 (cycles), and f∈[0.5,50] Hz. Spectral whitening, via $Z_{H0}$ -score normalization of each frequency bin across time samples as described in Dellavale et al. (2023), was included in the computation of the time–frequency maps to facilitate the visualization of the high-frequency components in the resulting ESM. The ESM can be defined at the single event level (see Fig. 2F), by averaging all the SEs in each subject (data not shown) and by averaging the mean ESM of each subject across participants (see Figs. 2F, G and 3A, B). Of note, the ESM does not represent the frequency content of SEs since the latter are very short-duration transient events, instead, the ESM reveals the spectral signature associated with the oscillatory activity co-occurring with each SE. That is, the ESM reveals the co-occurrence (or coupling) between the oscillatory activity and SEs across brain regions. To assess the statistical significance of the spectral signatures associated with the SEs, we compute pixel-level thresholding on the mean ESM with Bonferroni correction for multiple comparisons. More specifically, we computed the mean ESM on each one of the 100 B- or C-surrogate datasets (see Section 2.8). Then these 100 surrogate mean ESMs were used to compute pixel-level thresholding on the true mean ESM using a Bonferroni-adjusted two-tailed statistical threshold =0.05 / (Brain regions×Frequency bins). Note that this Bonferroni correction for multiple comparisons assuming independence between adjacent spatial/frequency bins of the mean ESM is a quite conservative test, yet, the observed spectral signature in the alpha band is evident even after this stringent thresholding process (see Fig. 2F, G).

### 2.6 Salient events waveform shape

To characterize the waveform shape of SEs, we follow an ROI-wise approach. First, in each brain region, we computed the average across the 200 ms signal epochs (absolute value) centered around the start time of the SEs of interest recruiting that particular region (see gray lines in Fig. 3C, D and Supplementary Fig. D.1C, D). Then, we obtained the mean SEs waveform shape by computing the average of the resulting time series across the brain regions (see the red and blue lines in Fig. 3C, D and Supplementary Fig. D.1C, D).

### 2.7 Salient events propagation modes

To assess the SEs starting modes, we assign to each brain region the number of events igniting in that particular site (e.g., see the RPre brain region in the activation matrix shown in Fig. 2A). Similarly, for the SEs ending modes, we assign to each brain region the number of events extinguishing in that particular site (e.g., see the RIC brain region in the activation matrix shown in Fig. 2A). For the SEs maximum recruitment modes, we assign to each brain region the number of events involving that particular site during the event maximum size (e.g., see the four brain regions active at Time≈51.591 sec in the activation matrix shown in Fig. 2A). Last, by dividing the event count obtained in each brain region by the total number of processed SEs, we obtained the mean spatial profiles for the starting, maximum recruitment and ending SEs modes as shown in the Supplementary Figures C.5, C.6, D.2 and D.3.

### 2.8 Surrogate datasets

We generated phase-randomized A-surrogate datasets, which preserve the PSD in each brain region, while disrupting the phase relationships of the spectral components (both within and between brain regions) (Prichard & Theiler, 1994). For this, in each brain region, we implemented a frequency-domain randomization procedure, which involves taking the Discrete Fourier Transform (DFT) of the time series, adding a random phase shift in the range [−π,π] on each spectral component of the DFT (preserving the odd phase symmetry associated with real signals (Bracewell, 2000)), and then taking the inverse DFT to obtain the surrogate signal back in the time-domain (Cohen, 2014). The 100 phase-randomized A-surrogate datasets were obtained by applying this procedure 100 times on each brain region independently. In addition, we also generated B-surrogate datasets that randomize the phases similarly to the A-surrogate, but in this case preserving both the regional PSDs and the cross-spectra. For this, we follow a similar procedure as described above with the difference that the same random phase shift was applied to all the brain regions. This implies that the phase difference between any pair of brain regions in *homologous frequency components* is preserved (i.e., preservation of cross-spectra). This implies to preserve the Pearson’s correlations between brain regions (see Supplementary Material A.1). Note that the B-surrogates destroy the phase relationships only between *non-homologous frequency components*. Finally, we generated 100 C-surrogate sets of SEs by randomizing the starting time of each observed salient event and keeping unaltered all the other properties such as the event duration and brain regions recruited in each event.

### 2.9 Clustering of salient events

SEs were clustered according to their spectral signature by using the Louvain method for community detection based on modularity maximization (Blondel et al., 2008; Rubinov & Sporns, 2011). First, the Matrix of Paired Distance (MPD) was obtained by computing the Euclidean distance between the vectorized ESMs corresponding to the SEs of interest taken in pairs. The resulting MPD (Events × Events) was normalized to be in the range [0,1], and the adjacency matrix (AM) was computed as AM = 1 − MPD. Then, the Louvain algorithm was repeated 100 times on the AM for resolution parameter values in the range 0.5≤γ≤2 (Blondel et al., 2008; Shafiei et al., 2019). Optimization of modularity quality function, based on the maximization of the similarity measure (z-scored Rand index) (Shafiei et al., 2019), was achieved for resolution parameter values within the range 0.9≲γ≲1.1 . Finally, a consensus partition was found from the 100 partitions (Lancichinetti & Fortunato, 2012; Bassett et al., 2013; Betzel, 2020). For the events detected in our source-reconstructed MEG dataset, the Louvain algorithm consistently identified two SE clusters with significant differences in terms of mean event duration, size, and spectral signature in their mean ESM (see Fig. 3; Supplementary Figs. C.11 and D.1).

### 2.10 Spectral group delay consistency measures

**Fig. 4.**
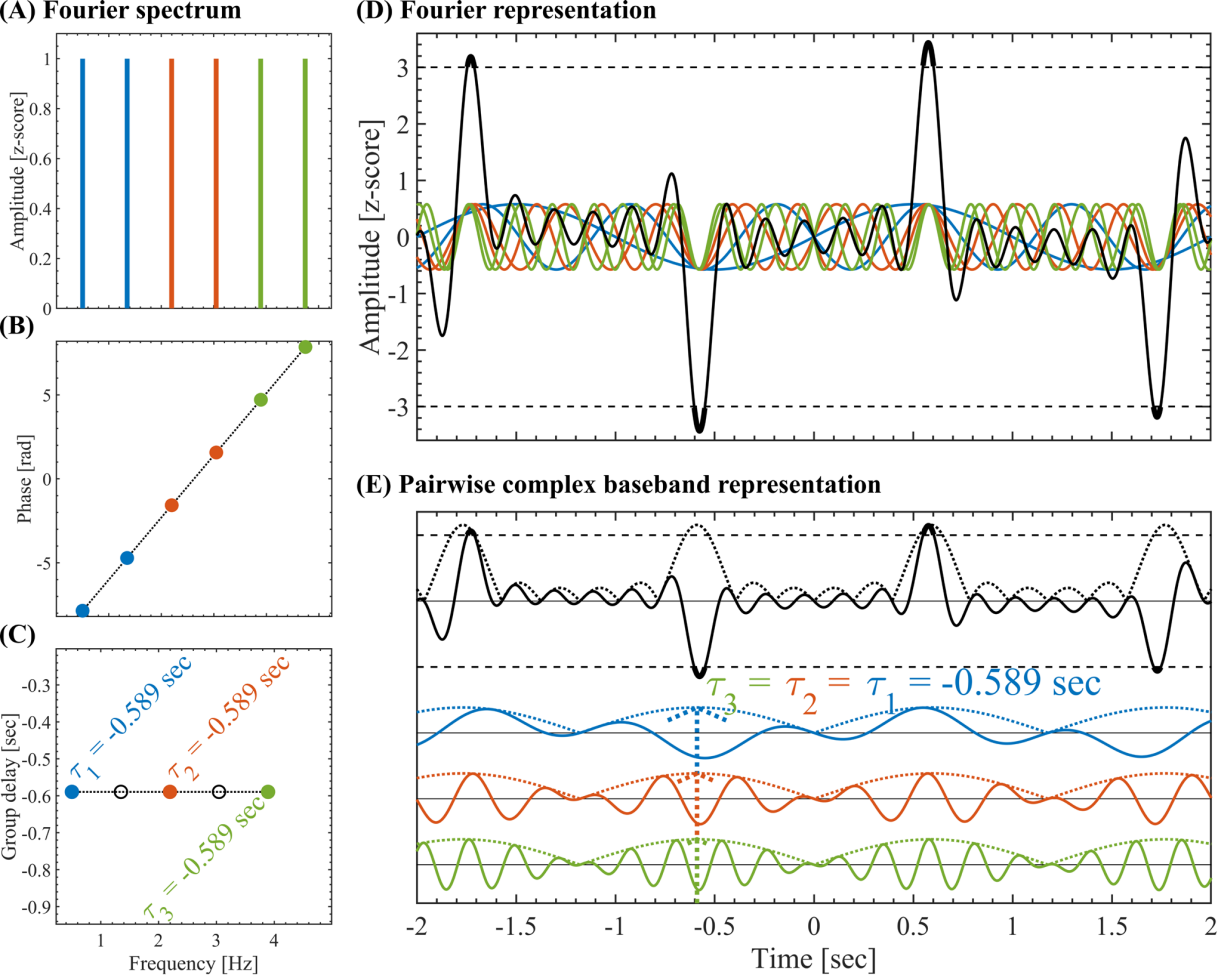
Pairwise complex baseband representation. (A) Set of constant-amplitude A(k)=1 oscillatory components uniformly spaced ($f_{s}\Delta \omega /\left(2\pi \right)=1.2/\sqrt{2}$ Hz) and having non-harmonic frequencies $f_{s}\omega \left(k\right)/\left(2\pi \right)=0.5+$ $kf_{s}\Delta \omega /\left(2\pi \right)\in \left[0.5-5\right]$ Hz, where $f_{s}=1024$ Hz is the sampling rate. The pairwise complex baseband representation (Eq. A.13) was obtained by grouping the oscillatory components in adjacent non-overlapping pairs color coded in blue, red, and green. (B) Phases ϕ(k) having a linear dependence as a function of the frequency within the range ϕ(k)∈2.5[−π,π]. (C) Group delay $\tau \left(k\right)/f_{s}=-\Delta \phi \left(k\right)/\left(f_{s}\Delta \omega \right)$ for the pairs of adjacent oscillatory components. The color-coded filled markers correspond to the $\tau \left(2k\right)/f_{s}$ values, and the black empty markers correspond to $\tau \left(2k+1\right)/f_{s}$ values (see Eq. A.13). (D) Z-scored signals associated with the Fourier representation. The solid color-coded lines represent the individual oscillatory components, the solid black line is the resulting signal x(t), the horizontal dashed black lines indicate the threshold at |z|=3 . (E) Pairwise complex baseband representation. The solid color-coded lines represent the individual amplitude-modulated signals (pairs of adjacent oscillatory components), the solid black line is the resulting signal x(t), the color-coded and black doted lines are the corresponding amplitude envelopes. For an in depth mathematical description of the pairwise complex baseband representation, see Supplementary Material A.2.

In this study, we introduce the pairwise complex baseband representation of band-limited signals (Eqs. A.7–A.10 and A.13–A.16; Haykin, 2001; Oppenheim et al., 1999; Proakis, 2008) to provide analytical arguments showing that the link between local above-threshold fluctuations and oscillations can be understood in terms of the group delay consistency across the spectral components (i.e., Fourier oscillatory constituents) of the neuronal activity. Specifically, in Supplementary Material A.2, we show that the time-domain representation of any finite-length time series x(t) (inverse DFT, Eq. A.5) can be re-arranged by grouping the Fourier spectral components X(k) in non-overlapping adjacent pairs, leading to the pairwise complex baseband representation (Eq. A.7). In this new representation (Eq. A.7), the signal x(t) is decomposed into a linear superposition of amplitude-modulated components, each synthesized from an adjacent pair of spectral components (X(2k), X(2k+1) in Eq. A.7). Crucially, Eq. A.7 explicitly shows that the group delay is the key spectral feature determining the transient synchronization of the Fourier oscillatory constituents of the signal x(t) leading to the emergence of SEs (see Fig. 4; Supplementary Eq. A.17 and Figs. A.2–A.4). More precisely, the group delay determines the time alignment of the amplitude-modulated oscillatory constituents of the signal x(t) in the pairwise complex baseband representation. Such time alignment promotes transient large-amplitude excursions of the signal (i.e., above-threshold fluctuations). Thus, Eq. A.7 constitutes a group delay-domain representation of the signal x(t), which lies in-between and links the time-domain and frequency-domain representations:

- *Time-domain representation*: Waveform shape of the x(t) (inverse DFT, Eq. A.5).
- *Group delay-domain representation*: Amplitude-modulated oscillatory constituents of x(t) defined by the adjacent pairs X(2k), X(2k+1) in Eq. A.7.
- *Frequency-domain representation*: Constant-amplitude oscillatory constituents of x(t) defined by the spectral components X(k) in the DFT (Eq. A.4).

We used the group delay-domain representation to analytically show that the emergence of SEs (i.e., above-threshold fluctuations) in the time-domain is associated with a high group delay consistency across the oscillatory components in the frequency-domain representation (i.e., approx. constant group delay disclosed by the Fourier constituents of the signal, see Supplementary Material A.2). This mathematical fact, conceptually illustrated in Figure 4, constitutes an essential signal-level feature inherent to the frequency-domain representation of time series and holds true regardless of both the x(t) waveform shapes and the underlying biological mechanisms associated with the analyzed SEs.

The group delay is defined as the rate of change of the phase with respect to the frequency, then, a constant group delay across the Fourier frequencies implies a constant incremental phase across frequencies (provided that Δω=const. ).

Thus, highly structured Fourier phase values, that is, incremental phase values disclosing low variability across frequencies, promote the time alignment of the amplitude-modulated components of the signal (see Fig. 4), and, therefore, the emergence of transient above-threshold fluctuations. To quantitatively assess this effect, we introduce the SGDC measures as described below.

The spectral group delay associated with the activity of the brain region r is defined as the rate of change of the phase $\phi_{r}\left(\omega \right)$ with the frequency ω computed on the Fourier spectrum of the brain activity (i.e., the DFT): $\tau_{r}\left(\omega \right)=-\Delta \phi_{r}\left(\omega \right)\;/\;\Delta \omega \left(\omega \right)$. The incremental phase $\Delta \phi_{r}\left(\omega \right)$ is defined as the phase difference between spectral components (adjacent in frequency ω) constituting the neural activity of the brain region r. The theoretical analysis presented in Supplementary Material A.2 shows that the spectral group delay consistency (SGDC) is an important feature linking the oscillatory properties of a signal to the above-threshold fluctuations associated with SEs. For an in-depth mathematical description of the oscillatory mechanisms eliciting above-threshold fluctuations in the brain signals and the measures quantifying the SGDC, the reader is referred to Supplementary Materials A.2 and A.3. Here, we briefly introduce the SGDC measures designed to efficiently quantify this feature in the experimental data,

$$SGDC\left(r\right)=\frac{1}{N}\sum_{\omega }e^{-i\Delta \phi_{r}\left(\omega \right)}:\Delta \omega =\text{const across }r$$ (1)

$$SGDC\left(\omega \right)=\frac{1}{N}\sum_{r}e^{-i\Delta \phi_{r}\left(\omega \right)}:\Delta \omega =\text{const across }\omega .$$ (2)

Eqs. 1 and 2 define the SGDC measures as the Euler transformed incremental phase values $\Delta \phi_{r}\left(\omega \right)$ averaged across the spectral components or brain regions, respectively, with N being the number of either frequency values or brain regions as appropriate. Importantly, the SGDC(r) measure (Eq. 1) assesses the emergence of local above-threshold fluctuations from the spectral components constituting the activity of the brain region r, whereas the SGDC(ω) measure (Eq. 2) quantifies the synchronization of the above-threshold fluctuations at the frequency ω across brain regions. We also define the pairwise spectral group delay consistency (pSGDC) to quantify the burstiness and cross-regional bursts synchronization in a single measure.

$$\begin{array}{llll} & & pSGDC\left(r_{1},r_{2}\right)= & \\ & & \underset{\text{Mean pairwise burstiness}}{\underset{︸}{\left(\frac{SGDC\left(r_{1}\right)+SGDC\left(r_{2}\right)}{2}\right)}}\underset{\text{Correlation of burstiness across}\;\omega }{\underset{︸}{\frac{1}{N}\sum_{\omega }e^{-i\left(\Delta \phi_{1}\left(\omega \right)-\Delta \phi_{2}\left(\omega \right)\right)}}} & \\ & & :\Delta \omega =\text{const across}\;r. & \end{array}$$ (3)

Eq. 3 shows that $pSGDC\left(r_{1},r_{2}\right)$ is a linear measure conformed by two factors: a factor quantifying the cross-regional correlation between the group delays across the frequency values, weighted by a coefficient quantifying the burstiness of the two involved brain regions ($r_{1},r_{2}$).

The SGDC measures (Eqs. 1, 2, and 3) were computed using both non-time-resolved and time-resolved approaches. In the non-time-resolved case, the SGDC measures (Eqs. 1, 2, and 3) were computed on the whole time series of each brain region. That is, we first obtain the phase values corresponding to the Fourier spectral components by computing the DFT (via the Fast Fourier Transform algorithm) on the whole time series of each brain region. Then, SGDC measures (Eqs. 1, 2, and 3) were computed on the incremental phase $\Delta \phi_{r}\left(\omega \right)$ obtained as the phase difference between the Fourier spectral components taken in non-overlapping adjacent pairs across the frequency ω. In particular, this non-time-resolved approach was used to produce the results shown in Figure 7 and Supplementary Figure A.7A, B. In contrast, in Supplementary Figures A.7C, D and C.11, we follow a time-resolved approach. That is, the SGDC(r) and SGDC(ω) measures (Eqs. 1 and 2) were computed on each detected SE by considering the brain regions and time interval associated with each particular event. In the case of Supplementary Figure C.11, the SGDC(r) and SGDC(ω) arrays were averaged selectively across the SEs segregated in the two clusters produced by the Louvain algorithm (see Section 2.9).

## 3 Results

### 3.1 Statistical, spatiotemporal, and spectral characterization of salient events

We identified SEs in our dataset and studied their characteristic signatures. In particular, we introduced a comprehensive array of features describing the statistical, spatiotemporal, and spectral properties of SEs. The proposed tools allowed for the characterization of SNEs by the way they spread across the brain network. Indeed, we found the role that each brain region plays in the propagation of these SNEs is not homogeneous. To characterize the spatiotemporal profiles of SEs, we defined two ROI-wise metrics (see Methods, Section 2.4): The *mean event duration* measures the typical duration of SEs propagating through a brain region (Fig. 1A, B); and the *mean event size* measures the typical size of SNEs propagating through a brain region (Fig. 1C, D). The brain plots in Figure. 1B and D reveal a characteristic topography, demonstrating the heterogeneous role that each brain region plays in the propagation of SNEs. In particular, SEs with bigger size and longer duration seem to be more associated with the temporal and deep brain regions.

Regarding the statistical characterization, we found that the SEs detected in our MEG data obtained from 47 subjects (1 min MEG time series source reconstructed to 84 brain regions) disclose exponential-like distributions of the events size and duration with steep slope exponents (≲−3 , see Supplementary Fig. C.3), which do not follow the power-law statistics putatively associated with the dynamical regime around a critical point or phase transition (see Introduction). Importantly, the exponential-like distributions of the events size and duration shown in Supplementary Figure C.3 do not modify significantly when the time binning value is varied from 1 to 5 samples per time bin (time binning ranging from 1 ms to 5 ms, data not shown).

Next, we introduce a tool to characterize the spectral signature of SEs, by first transforming the regional signals into a time–frequency representation and then averaging the time–frequency maps selectively across the time points corresponding to the occurrence of each SE (Fig. 2A–D). This way, we defined the spectral fingerprint of each SE, which we named Event Spectral Matrix (ESM, see Methods and Fig. 2E–G). Of note, the ESM does not represent the frequency content of SEs since the latter are very short-duration transient events, instead, the ESM reveals the spectral signature associated with the oscillatory activity co-occurring with each SE. That is, the ESM reveals the co-occurrence (or coupling) between the oscillatory activity and SEs across brain regions. Figure 2 displays the ESM for a single event (panel E), the average ESM across 10 subjects (panel F) and the ESM averaged across all the 47 subjects (panel G). Figure. 2F and G shows that the oscillatory activity of most brain regions peaks in the alpha band (8–13 Hz) during SEs. In other words, during SEs, brain regions fluctuate predominantly in alpha. This is also observed away from the occipital regions, suggesting that synchronization in the alpha band might spread on a large-scale during SNEs. Note that this result provides a relevant insight regarding the connection between SEs and NOs and, it is non-trivial since SEs are rare phenomena, occupying only a small fraction of the total recording (in space and time).

### 3.2 Salient events and phase coherence: surrogate data analysis

The spectral signature in the alpha band disclosed by the averaged ESMs shown in Figure. 2F and G suggests that a significant fraction of the SEs observed in our MEG data co-occur with (or are coupled to) alpha oscillations. To test this hypothesis, we generated 100 C-surrogate sets of SEs (see Methods, Section 2.8) that randomize the starting time of each observed SE and keep unaltered all the other properties such as the time width and brain regions recruited during each event. Importantly, as shown in Figure 2F, the average ESM of the true SEs thresholded with the average ESM of the C-surrogate SEs (see Methods, Section 2.5) discloses a prominent spectral signature in the alpha band. This result reveals a significant (i.e., above chance level) coupling between the true SEs and alpha oscillations, supporting our hypothesis that the large-scale spreading of transient alpha bursts is associated with SNEs. Taking together these results suggest that during SNEs, the brain activity displays large deviations from the baseline, which are coordinated across regions, giving rise to complex activation patterns with well-defined statistical, spatiotemporal, and spectral features.

To investigate the statistical properties of the signals associated with the emergence of realistic SEs, we first tested whether the observed SEs require additional features beyond the autocorrelation (PSD) of each MEG trace, which could include cross-correlation, non-stationarity, or non-Gaussianity. A common way to test the necessary and/or sufficient conditions underlying a phenomenon (here, SEs) is the use of surrogate data analysis (Prichard & Theiler, 1994). This approach involves creating surrogate datasets that remove or alter a specific property (e.g., phase relationships) while preserving other statistical characteristics, allowing one to determine whether the absence or modification of the property affects the observed feature of interest. Following this line of reasoning, we generated 100 phase-randomized A-surrogate datasets (see Methods). Each A-surrogate preserves the PSD (and thus the autocorrelation) of each brain region but disrupts the phase relationships of spectral components. When phases are randomized independently across regions, this procedure also disrupts inter-regional phase relationships and, therefore, removes cross-correlation structure that depends on those phases. Hence, A-surrogates implement the null hypothesis that the observed SEs can be explained solely by the preserved PSDs (i.e., by stationary, approximately Gaussian signals with inter-regional phase relationships removed). Despite the A-surrogates having the same spectral content as the original data, they disclose distributions with significantly less SEs with large size and duration values when compared with those observed in the true data (see A-surrogates in Supplementary Fig. C.3A, B). Besides, A-surrogates do not reproduce realistic spatiotemporal patterns of propagation (see A-surrogates in Fig. 1A, C) and ESMs (data not shown).

We then tested whether the observed SEs require additional structure beyond the auto- and cross-correlation of the MEG trace, which could include non-stationarity, or non-Gaussianity. To test this hypothesis, we generated 100 phase-randomized B-surrogate datasets (see Methods) that randomize the phases similarly to the A-surrogate, but in this case preserving both the regional PSDs and the cross-spectra. The preservation of cross-spectra implies that the phase difference between any pair of brain regions in *homologous frequency components* is preserved. This implies to preserve Pearson’s correlations between brain regions (see Supplementary Material A.1 and Fig. A.1). However, the B-surrogates destroy the phase relationships between *non-homologous frequency components*. B-surrogates, therefore, implement the null hypothesis that the observed SEs can be explained by the preserved auto- and cross-correlation (i.e., by stationary, approximately Gaussian signals with inter-regional phase relationships preserved). The observed mean spatiotemporal properties (see B-surrogates in Fig. 1A, C), the alpha signature disclosed by the ESM (see the average ESM thresholded using the B-surrogates shown in Fig. 2G), and the distributions of SEs duration and size (see B-surrogates in Supplementary Fig. C.3A, B) are not explained by the B-surrogates. Notice that these results are non-trivial, since in both the original and the B-surrogate datasets, the number of SEs is almost identical, and large events are also observed in the surrogate data (see B-surrogates in Supplementary Fig. C.3A, B).

To summarize, despite retaining the same power spectra and cross-spectra, the loss of synchronization across spectral components (given by the phase randomization) impairs large-scale coordinated SNEs, significantly disrupting the statistics and features of SEs.

### 3.3 Clustering of salient events

The ESM can be defined at the single event level (Fig. 2E). Thus, we asked whether SEs with different spectral signatures propagate differently. In particular, we hypothesized a relationship between the event spectral signature (as measured by the ESM) and the event duration, size, and propagation topographies (see Methods). To test this relationship, we clustered SEs according to their ESM using the Louvain algorithm (see Methods). We found that SEs cluster into two main groups based on their spectral signature (Fig. 3A, B). The SEs belonging to cluster 1 (Fig. 3A) display less marked and widespread alpha peak in the ESM as compared with cluster 2 (Fig. 3B). Importantly, we found statistically significant differences in the mean event duration and size between cluster 1 and cluster 2 (see Fig. 3E, H). To assess this, in each brain region, we computed a non-parametric permutation test (random sampling without replacement, $1\times 10^{4}$ permutations). All the brain regions disclosed a statistically significant difference of the mean event duration and size between clusters 1 and 2 (the Bonferroni-adjusted two-tailed P values result P<0.001 in all the brain regions). Consistently, the two clusters are also well distinguished by their different waveform shapes, with cluster 1 showing shorter temporal profiles of above-threshold fluctuations. Figure 3C and D shows the average waveform shapes of SEs, obtained by averaging in each brain region (BR) the absolute value of the time series associated with each event (see Methods).

These results suggest that cluster 2 events are specifically related to the long-range spread of narrowband alpha bursts across the brain network (i.e., SNEs), whereas cluster 1 events correspond to more short-lived and spatially localized fluctuations mainly promoted by the BAA (i.e., SLEs. See Fig. 3A–E, H). Consistently, the two identified clusters are also characterized by different event duration and size, which supports our hypothesis. In particular, cluster 1 events are generally small and short lived when compared with cluster 2 events, although both clusters display event size and duration distributions spanning across a few orders of magnitude (see Fig. 3G, J). Interestingly, the event duration and size distributions are different between the two clusters, which could have implications for the study of the spectral background statistics.

We also found that SEs propagate in a cluster-specific manner (see Figs. C.5 and C.6 in Supplementary Material C). In cluster 1, the spatial profiles associated with the events start, maximum recruitment and end are highly correlated (see Supplementary Fig. C.5A, pairwise Pearson’s correlations r>0.978 , P<0.001 two-tailed Student’s t-test), pointing out that cluster 1 events do not propagate to brain regions distant from those igniting the events. This result strongly supports the evidence presented above regarding the spatially localized nature of cluster 1 events. However, the spatial profiles associated with the events start and end are also highly correlated in cluster 2 SEs (see Supplementary Fig. C.5B, Pearson’s correlation r=0.895 , P<0.001 two-tailed Student’s t-test), suggesting that the brain regions involved in the ignition of a particular cluster 2 event tend to remain active until the event extinction. However, the maximum recruitment profile of cluster 2 events discloses a weak negative correlation with respect to the start spatial profile (Pearson’s correlation r=−0.298 , P<0.01 two-tailed Student’s t-test), supporting our hypothesis that cluster 2 events spread in the form of narrowband alpha bursts across the brain network. Intriguingly, the spatial profiles associated with the events start and end are highly correlated between the two clusters (see Supplementary Fig. C.6A–C, G–I), whereas a different scenario is observed in terms of how the brain regions are recruited by the two event clusters. Specifically, brain regions that are recruited by the longer events of cluster 1 will be recruited by the shorter events of cluster 2, and vice versa (see Supplementary Fig. C.6D). Within cluster 1, the longest SEs occupy the frontal and occipital regions (see Supplementary Fig. C.6E), whereas in cluster 2, associated with the spectral signature in the alpha band, the longest SEs are in the parietal and temporal regions (see Supplementary Fig. C.6F). The opposite trend is observed for the shortest SEs. In fact, performing Pearson’s correlations between the spatial profiles of cluster 1 and cluster 2 corresponding to the maximal size of recruitment across brain regions, we obtain a strong negative correlation (r=−0.841 , P<0.001 two-tailed Student’s t-test, see Supplementary Fig. C.6D). Note that the specificity of cluster 2 events, associated with transient above-threshold alpha bursts, in recruiting parietal and temporal brain regions cannot be trivially explained by the presence of elevated (steady) alpha oscillatory power, which is commonly observed in occipital brain regions during the eyes-closed resting state (see Fig. 2B and Supplementary Fig. C.4).

In summary, in this section we have introduced a comprehensive array of SE features, showing that rare, short-lived SEs propagating across the brain during spontaneous resting-state activity are highly structured in terms of their spatial, temporal, and spectral properties. In particular, the spectral characterization using the ESM provided relevant insights regarding the connection between the observed SNEs and NOs in the alpha band.

### 3.4 Analytical framework: Spectral group delay consistency

We next explored the mechanism mediating the reduction of local and cross-regional burstiness observed in our surrogate data computed via phase randomization (see Section 3.2). Notice that this is a relevant question since surrogate data analysis based on phase randomization is extensively used in many (bio)physical domains including neuroscience. Importantly, being the phase an intrinsic property of NOs, it is not obvious how the modification (e.g., randomization) of this oscillation-domain parameter affects the emergence of SEs (compare the true data with the A- and B-surrogates in Fig. 1 and Supplementary Fig. C.3). This question becomes apparent by taking into account that despite preserving both the power spectrum (PSD) in each brain region and the cross-spectra (i.e., functional connectivity), B-surrogates fail to account for the SEs observed in our MEG dataset. To address this question, we developed a signal-level analytical framework, named spectral group delay consistency (SGDC), designed to provide an analytical rationale supporting the emergence of SEs from the oscillatory constituents of the brain activity.

Let us focus on a single brain activity time series. We first compute the DFT to decompose the time series as a linear superposition of its Fourier oscillatory components (see Fig. 4A, B, D and Eq. A.4). Then, we group the Fourier components in (non-overlapping) pairs adjacent in frequency (see color-paired Fourier components in Fig. 4A). This led to the pairwise complex baseband representation of the time series. In this representation, the time series of interest is decomposed as a linear superposition of amplitude-modulated components (see the color-coded amplitude-modulated signals in Figure 4E and Eqs. A.7 and A.17). Importantly, the time offset of each amplitude-modulated component is determined by the spectral group delay τ(ω)≈−Δϕ(ω) / Δω , where τ(ω) is computed on Fourier spectrum of the brain activity (i.e., the DFT), as the rate of change of the phase ϕ(ω) with the frequency ω. Essentially, when all the Fourier components are added together to synthesize the signal in the time-domain (i.e., the inverse DFT), the spectral group delay determines the time alignment of the envelope of the amplitude-modulated components associated with each pair of adjacent spectral components. Such time alignment promotes transient large-amplitude excursions of the signal (i.e., above-threshold fluctuations). In the case of adjacent spectral components with phase values depending linearly with ω, we obtain approximately constant spectral group delay values for all the pairs of adjacent spectral components (see Fig. 4C). In such a case, the signal has a high spectral group delay consistency (SGDC) that promotes the time alignment of the amplitude-modulated components (see the color-coded amplitude-modulated signals in Fig. 4E), hence, supporting the occurrence of above-threshold fluctuations (see the large-amplitude excursions of the black time series in Fig. 4D, E). In contrast, for adjacent frequency components having phase values disclosing a nonlinear dependence with the frequency ω (e.g., a quadratic dependence as shown in Supplementary Fig. A.2G), the resulting spectral group delay depends on ω (see Supplementary Fig. A.2H). The latter disrupts the time alignment of the amplitude-modulated components (see the color-coded amplitude-modulated signals in Supplementary Fig. A.2J). In this case, we say that the signal has low SGDC that reduces the occurrence of above-threshold fluctuations (see the sub-threshold fluctuations of the black time series in Supplementary Figs. A.2I, J).

The results discussed above constitutes strong analytical arguments pointing out that the reduction of the local burstiness observed in our surrogate data computed via phase randomization can be understood in terms of the group delay consistency across the spectral components of the neuronal activity (i.e., SGDC). Specifically, the phase randomization process produces phase values having a nonlinear (random) dependence with the frequency of the Fourier components, hence, reducing the SGDC of the resulting surrogate time series. We confirmed this theoretical result using analytically tractable model of synthetic time series (see the discussion about Figs. A.2 and A.3 in Supplementary Material A.2), numerical simulations (see Section 3.5), and empirical MEG data (see the discussion about Fig. A.7A, C in Supplementary Material A.4). In particular, in Supplementary Material A.4, we analytically show that, despite preserving the regional power spectrum (PSD), the phase randomization associated with both A- and B-surrogates significantly reduces the burstiness of each brain region as assessed by the SGDC(r) measure (Eq. 1). Importantly, the reduction of the regional SGDC, as quantified by the SGDC(r) measure, offers an analytical rationale supporting the evidence showing that B-surrogates failed to reproduce the SEs observed in our MEG dataset despite preserving both the regional PSDs and the cross-spectra. As a conclusion, the SGDC constitutes a signal-level analytical model linking the emergence of SEs from the oscillatory components of the brain activity and underpining the evidence showing that our A- and B-surrogates computed via phase randomization failed to reproduce realistic SEs (see Section 3.2).

For an in-depth mathematical description of the SGDC framework and measures, the reader is referred to Supplementary Material A.2.

### 3.5 Numerical models: SEs, Nos, and BAA

**Fig. 5.**
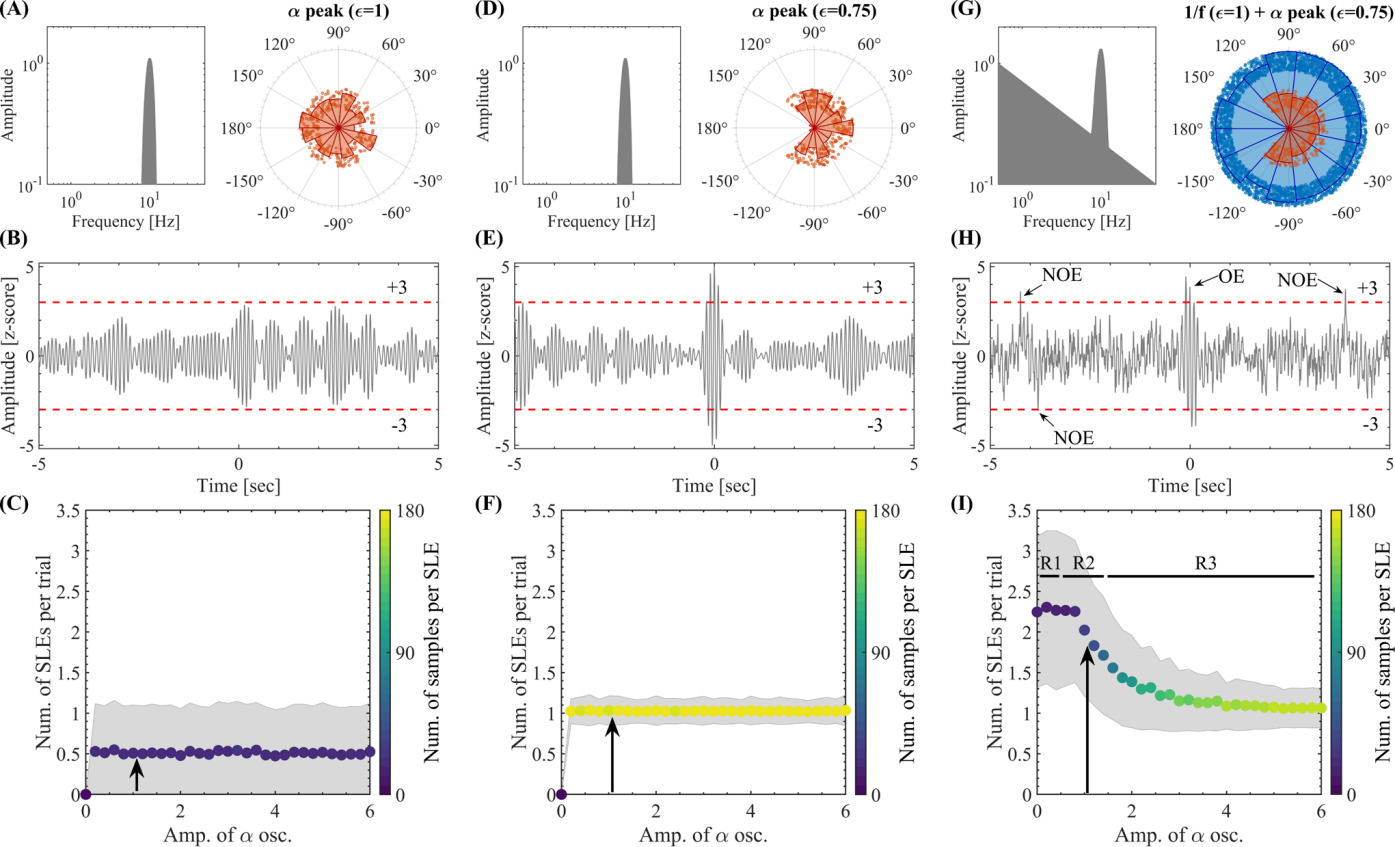
Model for local above-threshold fluctuations. (A) Amplitude spectrum (left) and distribution of the phase values assigned to the spectral components (right) for the oscillatory activity in the alpha band (Hann window with null-to-null bandwidth = 8–13 Hz, frequency resolution df=1/60sec≈0.017 Hz). Random phases were assigned to all the spectral components within the range [−ϵπ,ϵπ] with a phase factor ϵ=1 . (B) 10 sec epoch extracted from the synthetic time series produced by the amplitude spectrum and phase distribution shown in panel A (sampling rate of $f_{s}=1024$ Hz). The horizontal dashed lines in red indicate the 3 standard deviation (±3σ ) thresholds used to compute the SLEs as above-threshold amplitude fluctuations. (C) Number of SLEs per trial as a function of the maximum amplitude of the oscillatory activity in the alpha band. For each maximum amplitude value, we counted the number of SLEs across 1000 time series of 10 sec in duration (trials) synthesized as the one shown in panel B. In each trial, we recomputed the random phases of the spectral components within the range [−ϵπ,ϵπ] with ϵ=1 . The colored markers indicate the mean number of SLEs per trial across the 1000 trials. The shaded error bars in gray correspond to the standard deviation around the mean value. The pseudocolor scale represents the mean value for the number of above-threshold samples per SLE. The black arrow indicates the maximum amplitude of the alpha oscillations used in panels A and B. (D–F) Same as in A–C for spectral components with random phases constrained within the range [−ϵπ,ϵπ] with ϵ=0.75 (see the distribution of the phase values in panel D right). (G) Amplitude spectral profile (left) resulting from the linear superposition of (1) a narrowband amplitude spectrum around the alpha band (Hann window with null-to-null bandwidth = 8-13 Hz), and (2) a set of spectral components with power $A^{2}\left(f\right)\propto 1/\;f$ (frequency resolution df=1/60sec≈0.017 Hz). The right side of panel G shows the distribution of phase values assigned to the spectral components. Random phases within the range [−ϵπ,ϵπ] with ϵ=1 were assigned to the spectral components constituting the 1/ f background (blue circles) and ϵ=0.75 were assigned to the spectral components associated with the alpha bump (red circles). (H) Same as in B and E for the spectrum shown in panel G. In this case, it is possible to distinguish oscillatory events (OEs) and non-oscillatory (NOEs) salient local events. (I) Same as in C and F for the spectrum shown in panel G. R1, R2, R3 indicate regions characterized by *Amp. of alpha oscillations* less than, approx. equal to and greater than the *Amp. of* 1/ f *activity*, respectively. Symbols and abbreviations: SLEs, salient local events; OEs, oscillatory salient local events; NOEs, non-oscillatory salient local events.

We built a numerical signal model to elucidate the relationship between SEs, NOs, and BAA. We model the activity of single brain regions as the linear superposition of Fourier components oscillating in a narrow frequency band. As a result, the corresponding spectral representation discloses a “bump” of (null-to-null) bandwidth in the alpha band (8–13 Hz, Fig. 5A, D, G). The BAA was modeled by imposing a 1/ f trend in the PSD of each signal (Fig. 5G). This 1/ f spectral background was chosen to mimic the −10dB / dec log-log decay rate observed in the PSDs associated with our MEG dataset (see Supplementary Fig. C.4). To model different degrees of phase coherence, we assign random phase values to the spectral components within a range [−ϵπ,ϵπ] with ϵ∈[0,1] (see the polar plots in Fig. 5A, D, G). On the one hand, for ϵ≃1 , the spectral components of the signal were desynchronized (i.e., independent oscillatory components, Fig. 5A). On the other hand, for ϵ≃0 the spectral components were highly synchronized (i.e., high cross-frequency coherence). We first focused on a *single brain signal* and measured the number of SLEs (i.e., transient amplitude excursions above a fixed threshold of 3 standard deviations: ±3σ ) across 1000 realizations (i.e., trials), depending on the presence or absence of coherent NOs and 1/ f activity (see Fig. 5C, F, I). In the absence of 1/ f activity and for uniformly distributed random phases assigned to the spectral components in the alpha band (ϵ=1 , Fig. 5A), the model displays very few above-threshold fluctuations across trials (Fig. 5B, C). Increasing the coherence of the spectral components in the alpha band (ϵ=0.75 , Fig. 5D), despite the absence of 1/ f activity, the number of above-threshold fluctuations increased, producing a salient burst in most of the trials (Fig. 5E, F). Importantly, Supplementary Figure B.1 shows that the results discussed above, in connection with the emergence of local above-threshold fluctuations from the Fourier oscillatory constituents of the brain activity (i.e., SLEs), can be understood in terms of the SGDC as quantified by the SGDC(r) measure (Eq. 1). Specifically, Supplementary Figure B.1 shows that the increase of the signal burstiness, as quantified by the kurtosis of the signal’s amplitude values, associated with more constrained random phase values (i.e., low phase factor ϵ values) correlates with the increase in the SGDC as quantified by the SGDC(r) measure. In Supplementary Figure B.1, the time series were synthesized by adding pure sinusoidal signals. The SGDC(r) was then computed directly from the synthetic phases of these sinusoidal components. Because the phases were taken from the exact analytical components, no spectral leakage was present in this case. In contrast, in Supplementary Figure B.2, 1 min in duration time series were synthesized following the same procedure as in Supplementary Figure B.1, but this time the SGDC(r) was computed using the alpha band phases obtained from the DFT applied to the synthesized time series. This procedure inherently introduces spectral leakage due to the time-domain tapering (rectangular window), which affects the phase values involved in the computation of the SGDC(r) measure and is visible in the corresponding power spectra. Supplementary Figure B.2 shows that the increase of the salience of transient fluctuations in a signal, as quantified by the kurtosis of the signal’s amplitude values, is reproduced by the SGDC(r) measure. Importantly, these results highlight that the SGDC(r) measure is not primarily driven by the spectral leakage. Instead, it reflects the relationship between the salience of transient fluctuations and the consistency (spread or variability) of the group delay across the Fourier frequencies, independently of the spectral leakage. In addition, we re-compute the signal model for the same set of phase factor values used in Supplementary Figure B.1, this time using spectral phase values disclosing not a random but a linear dependence with the frequency (i.e., a time-shift in the time-domain). The results obtained with this configuration are shown in Supplementary Figure B.3. As predicted by the SGDC mechanism (see Fig. 4A–E), we obtained |SGDC(r)|≈1 independently of the phase factor value (ϵ∈[0,1]), and the time series produced by the signal model disclosed (time-shifted) above-threshold fluctuations in all the cases (see Supplementary Fig. B.3). These numerical results constitute further evidence showing that the SGDC effectively underlies the emergence of local above-threshold fluctuations from NOs, as in the case shown in Figure 5D–F. Then, we introduced the broadband 1/ f activity into the model through a linear superposition (addition) with the oscillatory activity in the alpha band. As a result, the presence of the broadband 1/ f activity with ϵ=1 and coherent spectral components in the alpha band with ϵ=0.75 (Fig. 5G) further increased the number of salient events in a single brain signal (Fig. 5H, I). Importantly, the 1/ f activity also influences the rhythmicity of above-threshold fluctuations, which occur aperiodically. More specifically, if we synthesize a long time series by concatenating trials constructed without the 1/ f activity (as in Fig. 5E), the concatenated time series will disclose a periodic series of above-threshold alpha bursts (i.e., one salient alpha burst per trial). Instead, in the presence of 1/ f activity, we obtain above-threshold fluctuations occurring aperiodically in each trial besides the salient alpha burst, hence, the time series resulting from concatenating trials (as in Fig. 5H) will disclose an aperiodic series of above-threshold fluctuations, elicited by the interaction between the 1/ f and oscillatory activities. Furthermore, the regime R2 in Figure 5I points out a plausible range for the relative amplitude between NOs and the BAA in order to obtain realistic aperiodically occurring above-threshold fluctuations. That is, in the regime R1 only non-oscillatory salient events (NOEs) are observed, in the regime R3 only oscillatory salient events (OEs) are observed. In contrast, the regime R2 is characterized by a stochastic resonance-like effect in which the resulting local activity exhibits both NOEs and OEs mirroring the two SE clusters observed in our MEG dataset. In Supplementary Material C.2 (Figures C.10 and C.11), we discuss additional empirical evidence supporting the theoretical findings described in Sections 3.4 and 3.5.

In summary, these results suggest that the mere presence of oscillations associated with an increase of power around a narrow frequency band does not guarantee the stable occurrence of above-threshold fluctuations (Fig. 5A–C). However, if the phases of the spectral components are coherent producing high |SGDC(r)| values, then high-amplitude fluctuations are consistently observed in the signal (Fig. 5D–F).

**Fig. 6.**
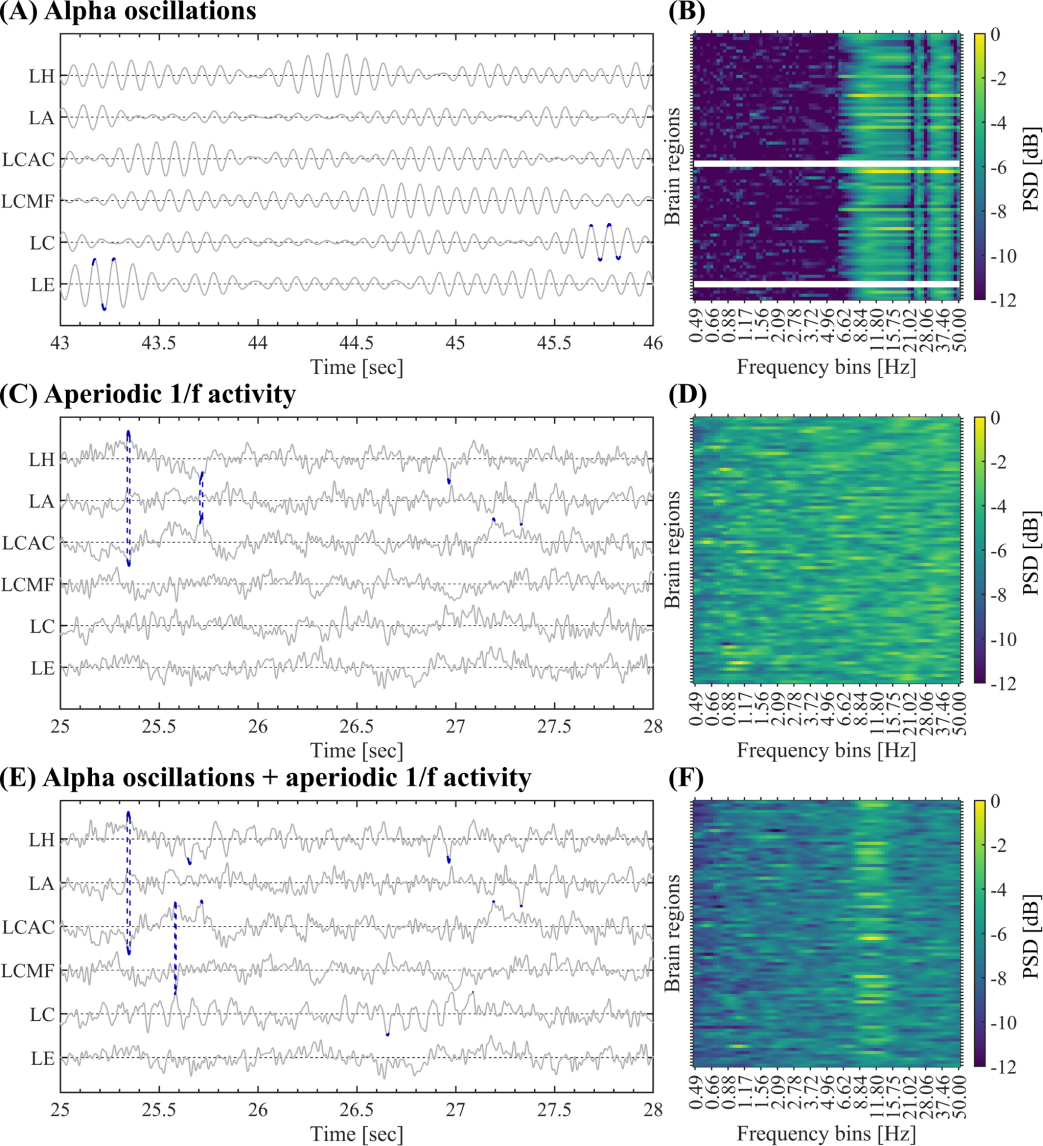
Large-scale signal model for SEs. (A, B) Large-scale model for SEs including only alpha oscillations (random phase values in the alpha band constrained to the range [−ϵπ,ϵπ] with ϵ∈[0.75,1]). Panel A shows a subset of synthetic activities. In each time series, the above-threshold fluctuations (±3σ ) are highlighted in dark blue. Vertical dashed lines connect the activations associated with SEs completely contained in the subset of signals shown. Panel B shows the resulting ESM averaged across the SEs. Panels A and B were computed on all the SEs detected in a simulated time series of 1-min duration. (C, D) Same as in A and B for the large-scale model including only broadband 1/ f activity, and no oscillatory activity in the alpha band nor phase consistency values were present (ϵ=1 ). (E, F) Same as in A and B for the large-scale model including both broadband 1/ f activity with non-constrained random phases (ϵ=1 ) and alpha oscillations with random phases constrained proportionally to the observed alpha power in the range (ϵ∈[0.75,1]). Symbols and abbreviations: SEs, salient events; LH, left hippocampus; LA, left amygdala; LCAC, left caudal anterior cingulate; LCMF, left caudal middle frontal; LC, left cuneus; LE, left entorhinal.

Next, we extended the above setup to model *whole-brain activity* and SNEs. For each simulated brain signal, we set the amplitude of the alpha peak (with alpha amplitude ∈[0,1]) proportionally to the mean alpha amplitude (average across the 47 participants) observed in the empirical MEG recordings, thus modeling the non-homogeneous presence of alpha activity across brain regions. In addition, in each region, we bounded the random phases assigned to the spectral components in the alpha band within a range [−ϵπ,ϵπ], whose width ϵ∈[0.75,1] was inversely proportional to the empirical alpha power (i.e., the higher the alpha peak, the higher the phase coherence among the spectral components). This choice was motivated by the fact that high PSD bumps are generally interpreted as stronger narrowband synchronization within local neuronal populations (Fries, 2005) (see Discussion). Using this setup, we measured synthetic SEs and tested their dependence on the 1/ f activity. When only alpha oscillations were present, and no broadband 1/ f activity (Fig. 6A), the resulting ESM was not realistic compared with the empiric one (compare Figs. 6B and 2G), and the distributions of SEs duration and size were not approximating the exponential-like distributions observed in our MEG dataset (compare Supplementary Figs. B.4A, B and C.3). Instead, when only broadband 1/ f activity was present, and no oscillatory activity in the alpha band nor coherent phase values were used (i.e., ϵ=1 ; Fig. 6C), the ESM did not show the spectral signature associated with the alpha component (Fig. 6D). Also, the distribution of SEs duration was similar to the empirical data, while the distribution of SEs size was shrunk, as the model did not display SEs involving large populations (Supplementary Fig. B.4C, D). Finally, when both broadband 1/ f activity and alpha oscillations were simultaneously present (Fig. 6E), the emerging SEs displayed a realistic ESM (compare Figs. 6F and 2G) as well as exponential-like distributions of SEs duration and size (Supplementary Fig. B.4E, F); although the SEs size decayed in a markedly more rapid fashion than in the empirical data (compare Supplementary Figs. B.4E, F and C.3). The Pearson’s correlation between the vectorized versions of the empirical (Fig. 2G) and simulated (Figs. 6B, D, F) ESMs is as follows: Empirical (non-thresholded version of the ESM shown in Fig. 2G) vs. large-scale model including only alpha oscillations (ESM shown in Fig. 6A): r=0.594 , P<0.001 . Empirical (non-thresholded version of the ESM shown in Fig. 2G) vs. large-scale model including only broadband arrhythmic activity (ESM shown in Fig. 6D): r=0.167 , P<0.001 . Empirical (non-thresholded version of the ESM shown in Fig. 2G) vs. large-scale model including both alpha oscillations and broadband arrhythmic activity (ESM shown in Fig. 6F): r=0.611 , P<0.001 . The statistical significance of these linear correlations was assessed by using the Student’s t distributions of the two-tailed hypothesis test under the null hypothesis that the correlation is zero. These results suggest that both NOs and broadband 1/ f spectral background contribute to the signal deviations from baseline activity and realistic SEs, provided that the narrowband spectral components display appropriate levels of SGDC.

### 3.6 Mechanisms of long-range interactions

Whereas the SGDC(r) assesses the emergence of local above-threshold fluctuations from the Fourier oscillatory constituents of the activity in a single brain region (i.e., SLEs), it does not account for cross-regional effects associated with SNEs. To quantitatively study the cross-regional effects of SGDC on our data, we introduce the SGDC(ω) measure (Eq. 2). The magnitude of SGDC(ω) is bounded in the range [0,1] and quantifies how much the group delay at a given frequency ω varies across brain regions. By using synthetic time series, in Supplementary Material A.3 we show that the SGDC(ω) measure assesses the contribution of each frequency component in the co-activation (synchronization in time) of above-threshold fluctuations across brain regions (see Supplementary Figs. A.5 and A.6). Of note, Supplementary Figures A.5 and A.6 show that the SGDC(ω) measure effectively resolves the cross-regional synchronization of SEs across frequency bands, whereas phase coherence measures (e.g., PLV: phase locking value) (Berens, 2009; Lachaux et al., 1999; Tass et al., 1998) are completely blind to this effect (see detailed description in Supplementary Material A.3). In Supplementary Material C.2 (Figures C.10 and C.11), we present additional empirical evidence supporting the connection between the SGDC(r) and SGDC(ω) measures and the emergence of local and large-scale salient events. In particular, Supplementary Figure C.11C shows that only cluster 2 SEs, associated with the spectral signature in the alpha band, disclose |SGDC(r)| values higher than those disclosed by the C-surrogate SEs. Importantly, Supplementary Figure C.11D shows the increase of transient cross-regional coherence around the alpha band, as quantified by the SGDC(ω) measure, associated with the SEs disclosing the alpha spectral signature in the average ESM (i.e., cluster 2 SEs). Notably, Supplementary Figure C.11E shows that the transient cross-regional coherence around the alpha band associated with the cluster 2 SEs is also captured by the large-scale model presented in Section 3.5.

**Fig. 7.**
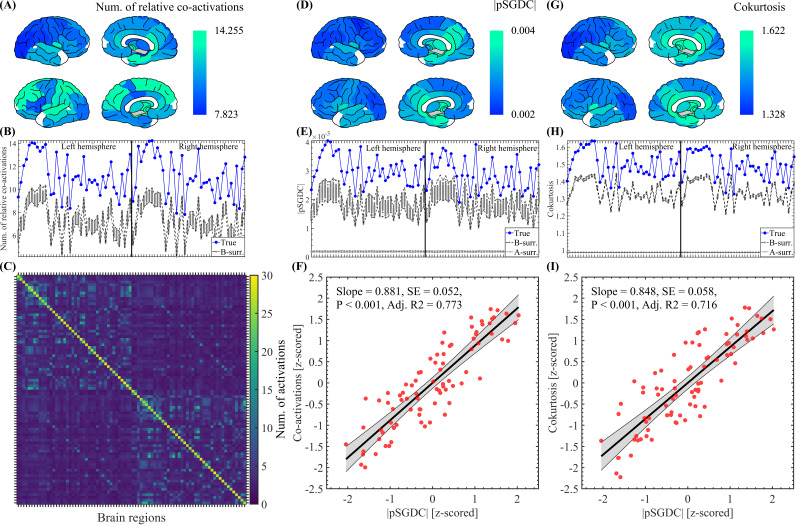
Co-activation pattern of SEs compared against pSGDC and cokurstosis measures computed on whole time series of the brain regions taken in pairs. (A) Brain topographies corresponding to the co-activation profile shown in panel B (blue markers). (B) Spatial profile showing the number of relative co-activations (mean value across the 47 participants), that is, the accumulated number of activations in each row of the co-activation matrix relative to the total number of activations in each brain region (diagonal of the co-activation matrix). Note that the spatial profiles corresponding to the 100 B-surrogates (dark gray markers) fail to reproduce the spatial profile associated with the true MEG data (blue markers). (C) Co-activation matrix averaged across the SEs observed in the 47 participants (see Section 2.3 in Methods). (D-E) Same as in A-B for the pSGDC measure. (F) Scatter plot showing the correlation between the co-activation and pSGDC spatial profiles shown in panels B and E, respectively. Number of samples (red circles) = Number of brain regions = 84. The thick black line and black shaded error bars represent the linear regression and the 95% confidence interval, respectively. The reported P value for the statistical significance of the linear regression was assessed using Student’s t distributions of the two-tailed hypothesis test under the null hypothesis that the correlation is zero. (G, H) Same as in D and E for the cokurtosis measure. (I) Same as in F for the correlation between the cokurtosis and pSGDC spatial profiles. In panels B, E, and H, the labels and ordering of the brain regions are the same as those shown in Supplementary Figure C.2. Symbols and abbreviations: SEs, salient events; pSGDC, pairwise spectral group delay consistency.

Synchrony is thought to play a role in coordinating information processing across different brain regions. However, correlation structures such as hemodynamic functional connectivity are better explained in terms of power amplitude correlations of electrophysiological signals (e.g., MEG), rather than phase synchrony. In a recent work, it was demonstrated that power correlation between two signals can be analytically decomposed into signal coherence (a measure of phase synchronization), cokurtosis (a measure of the probability of simultaneous large fluctuations), and conjugate coherence (Hindriks & Tewarie, 2023). In particular, it was proposed that the cokurtosis between two signals provides a measure of co-bursting that offers a robust neurophysiological correlate for hemodynamic resting-state networks (Hindriks & Tewarie, 2023). Here we show that the SGDC conceptualization provides a coherent account of both the co-burstiness and the cokurtosis in terms of the group delay consistency of the signals’ spectral content, therefore, advancing our understanding of the signal-level mechanisms of long-range communication. For this, we counted the co-participation of pairs of brain regions across SEs (see Methods, Section 2.3). Figure 7C shows the co-activation matrix indicating the number of co-activations between each pair of brain regions. Figure 7B shows the number of relative co-activations, that is, the accumulated number of activations in each row of the co-activation matrix relative to the total number of activations in each brain region (diagonal of the co-activation matrix). Figure 7A displays the brain plots corresponding to the number of relative co-activations shown in Figure 7B. Importantly, the topography of co-activations shown in Figures 7A–C cannot be trivially explained by the chance co-occurrence of rare above-threshold fluctuations in the brain activity. Note that the B-surrogates shown in Figure 7B fail to reproduce the topography of co-activations despite preserving both the power spectrum (PSD) in each brain region and the cross-correlations (i.e., functional connectivity). Moreover, we found that the kurtosis and SGDC(r), two measures related to the occurrence of local above-threshold fluctuations (i.e., SLEs), when computed in a non-time-resolved manner in each brain region, fail to reproduce the topography of co-activations associated with the observed SEs. In the case of the SGDC(r) measure, compare the spatial profiles shown in Supplementary Figure A.7A and Figure 7B. To account for both the burstiness and cross-regional bursts synchronization in a non-time-resolved manner, we used the pairwise SGDC measure (pSGDC). The $pSGDC\left(r_{1},r_{2}\right)$ is defined as the product of two fators: a factor quantifying the cross-regional correlation between the group delays across the frequency components, weighted by the average SGDC(r) of each pair of signals $r_{1}$ and $r_{2}$ (see Eq. 3 in Methods and Eqs. A.23 and A.24 in Supplementary Material A.3). In Hindriks & Tewarie (2023), it was analytically shown that power correlation depends on signal coherence, cokurtosis, and conjugate coherence. In particular, co-occurring bursts in neuronal activity, statistically measured by cokurtosis, are relevant for our discussion of SNEs. We computed the pSGDC and cokurtosis (Eq. A.26) measures on our MEG dataset by using the whole time series of the brain regions taken in pairs (i.e., non-time-resolved approach). As a result, we found that the pSGDC measure and the cokurtosis disclose a similar correlation degree with the observed co-activations topography (compare Fig. 7F and I) and generate statistics that are lost in the A- and B-surrogates (see Fig. 7E and H). Linear correlations between topographies: Co-activations vs pSGDC, r=0.881 , P<0.001 (Fig. 7F). Cokurtosis vs pSGDC, r=0.848 , P<0.001 (Fig. 7I). Co-activations vs cokurtosis, r=0.937 , P<0.001 (Fig. 7B, H). Co-activations vs pairwise Pearson’s correlation, r=0.612 , P<0.001 (Fig. 7B; Supplementary Fig. A.1B). The statistical significance of these linear correlations was assessed by using the Student’s t distributions of the two-tailed hypothesis test under the null hypothesis that the correlation is zero. The pSGDC measure quantifies the co-occurrence of above-threshold bursts mainly associated with SGDC in the alpha band, whereas cokurtosis assesses the presence of both oscillatory and non-oscillatory co-burstiness across brain regions. Importantly, the analytical framework proposed in this work based on the SGDC(r), SGDC(ω), and $pSGDC\left(r_{1},r_{2}\right)$ measures admits relevant signal-level mechanistic interpretations linking the Fourier oscillatory constituents of the brain activity and SEs. Note that the latter is less evident when considering measures based on higher-order statistical moments such as the kurtosis and cokurtosis. Specifically, using the group delay-domain representation, one can quantify the group delay consistency of the spectral (Fourier) constituents of the signals of interest (via the SGDC measures) to predict the emergence of SEs (without doing any explicit computation in the time-domain). This prediction linking the oscillation and time-domains cannot be done by higher-order statistical moments like the kurtosis and cokurtosis, mainly because they operate exclusively in the time-domain. Therefore, the SGDC framework provides a deeper understanding of the link between the oscillation-domain (Fourier representation) and the emergence of transient, salient fluctuations in the time-domain. Thus, the SEs co-activation pattern reproduced by the pSGDC measure (see Fig. 7A–F) can be mechanistically segregated in two components: (1) the results associated with the SGDC(r) measure (Fig. C.11C) supporting the emergence of local above-threshold fluctuations via SGDC mainly in the alpha band and (2) the results associated with the SGDC(ω) measure (Fig. C.11D) supporting the co-occurrence of above-threshold alpha bursts across brain regions (i.e., transient cross-regional coherence around the alpha band). We speculate that component 1 can be interpreted as an entrainment mechanism that produces transient synchronization of the oscillatory activity of neuronal populations around specific frequency bands (local cross-frequency synchronization), whereas component 2 can be associated with long-range interaction mediated by transient cross-regional coherence in NOs.

In summary, these results suggest that (a) spectral group delay consistency in specific narrow frequency bands (as assessed by the SGDC(r) measure), (b) transient cross-regional coherent NOs (intra-frequency coherence across brain regions assessed by the SGDC(ω) measure), and (c) BAA are all key ingredients for the emergence of realistic SEs. In particular, the (pairwise) long-range interactions mediated by oscillatory SNEs can be effectively quantified using the $pSGDC\left(r_{1},r_{2}\right)$ measure.

## 4 Discussion

Frequency-domain representation of signals, via Fourier transforms (e.g., DFT), has been extensively used for decades in many neuroscience fields to analyze neuronal and brain activities across several spatiotemporal scales. Regardless of the functional significance of neural oscillations, if any, the Fourier basis functions provide an arguably good characterization of the rhythmic components observed in the brain activity. In this study, we used the complex baseband representation of signals, based on the Fourier theory, to analytically define the spectral group delay consistency (SGDC) as a novel conceptualization linking SEs with the signals’ spectral content. Importantly, the signal-level analytical framework associated with the SGDC concept allowed us to provide a unifying rationale for the emergence of salient local and large-scale events from the Fourier oscillatory constituents of the brain activity. First, the analytical arguments described in Sections 3.4, 3.5, and Supplementary Material A.2 point out that in order to observe realistic local above-threshold fluctuations, the spectral components constituting the brain signals must disclose a certain degree of cross-frequency coherence as assessed by the SGDC(r) measure. Second, in Section 3.4 and Supplementary Material A.4, we analytically showed that A- and B-surrogates failed to reproduce realistic SEs mainly because the phase randomization reduces the SGDC across frequency bands in each brain region, which impairs the burstiness of each signal (occurrence of local above-threshold fluctuations). In the case of the A-surrogates, the phase randomization also reduces the SGDC across brain regions in each frequency band, which impairs the synchronization of above-threshold fluctuations across brain regions. Third, in Section 3.1 we showed that the spectral signature in the alpha band disclosed by the averaged ESM of cluster 2 SEs constitutes relevant evidence linking the observed SEs with NOs. Importantly, in Section 3.6 and Supplementary Material C.2, we demonstrated that the synchronization of above-threshold alpha bursts across brain regions can be described at the signal level by the SGDC mechanism. Specifically, we showed that the SNEs disclosing the alpha spectral signature in the average ESM (see cluster 2 in Supplementary Fig. C.11B) also disclose an increase of transient cross-regional coherence around the alpha band, as quantified by the SGDC(ω) measure (see cluster 2 in Supplementary Fig. C.11D). Of note, the SGDC(ω) measure effectively captures transient, cross-regional coherent NOs associated with SNEs, a phenomenon that traditional coherence metrics, such as the phase locking value (PLV) (Berens, 2009; Lachaux et al., 1999; Tass et al., 1998), fail to detect (see Supplementary Figs. A.5 and A.6). Thus, we combine analytical arguments, based on the SGDC framework, with experimental evidence obtained using novel tools such as the ESM and SGDC measures, to provide a more direct and generative link for NOs (e.g., alpha oscillations) role in the coordination of SNEs observed in spontaneous MEG activity. This moves beyond mere correlation or characterization to offer a plausible generative model for SNEs as spatiotemporal cascades of above-threshold fluctuations associated with phase-structured NOs. Fourth, the SGDC conceptualization allowed us, via the $pSGDC\left(r_{1},r_{2}\right)$ measure, to account for both the co-activation pattern of brain avalanches and cokurtosis in terms of the coherence of the signals’ spectral content, therefore, advancing our understanding of the signal-level mechanisms of long-range communication. The empiric, modeling, and analytical results presented in this work guided us to identify the essential building blocks underlying the emergence of realistic SEs as observed in our MEG dataset, which can be summarized as follows:

1. Spectral group delay consistency. This feature provides a signal-level mechanism for the emergence, in a single brain region (i.e., locally), of transient above-threshold fluctuations associated with a specific frequency band (e.g., alpha bursts). We speculate that the SGDC (e.g., bounded phase differences across spectral components within a narrowband) may be associated with the presence of mesoscopic neural oscillators that are not tightly tuned. We hypothesize that different brain regions may host mesoscopic oscillators disclosing rhythmic (likely non-sinusoidal) dynamics whose fundamental frequencies span a quasi-continuum within a given frequency band (e.g., alpha band), rather than clustering around a single sharply defined value. Thus, the linear superposition of these rhythmic components with slightly different frequencies within a narrowband (e.g., alpha range) could support the emergence of SEs via the SGDC signal-level mechanism.
2. Transient cross-regional coherent alpha oscillations. This feature is associated with the transient synchronization of the above-threshold alpha bursts across brain regions, giving rise to the SNEs producing the alpha spectral signature in the ESM (i.e., cluster 2 SEs). This type of SEs may be associated with a long-range interaction mechanism mediated by specific NOs taking place in a transient manner (i.e., transient CTC).
3. BAA. This feature is associated with the emergence of non-oscillatory above-threshold fluctuations occurring in an aperiodic manner, mainly related to the short-lived SEs with no characteristic spectral signature in the ESM (i.e., cluster 1 SEs). We hypothesize that the close relationship between cluster 1 SEs and arrhythmic broadband spectral features implies that cluster 1 SEs may play a more local role, linked either to local excitation–inhibition balance or to critical dynamics (Nanda et al., 2023).

Linking the presence of SEs to the group delay consistency across the Fourier oscillatory components of the brain activity is a relevant result of this study implying that SEs might mediate interactions across both frequency bands and brain regions as discussed above. In this regard, the CTC hypothesis posits that neural communication is facilitated by the presence of synchronized (steady) oscillations across brain regions. Our results extend the CTC hypothesis by showing that long-range interaction through specific NOs may take place in a transient manner via SNEs (i.e., transient CTC). Indeed, our results suggest that the large-scale spreading of transient alpha bursts is associated with SNEs. As a conclusion, this evidence suggests that transient cross-regional coherence associated with the occurrence of SEs disclosing the spectral signature in the alpha band (i.e., cluster 2 SEs) may play a functional role as a long-range interaction mechanism in the resting human brain.

One of the main limitations of this study is related to the uncertain capability of our dataset to accurately identify deep brain sources along the cortical surface, mainly due to the ill-posed nature of the source-reconstructed MEG data. In order to address this issue, we re-computed the analysis of SEs presented above, but this time excluding the deep sources. It was found that all the conclusions and, in particular, all the characteristics of the observed SEs remain essentially unaltered when the deep sources are excluded from the SE analysis (see Supplementary Material D). Specific analyses demonstrating that volume conduction alone is unlikely to account for the cascade of above-threshold fluctuations (i.e., SNEs) observed in our empirical MEG dataset have been presented and discussed in a previous publication (Sorrentino et al., 2021b). The spatial leakage analyses and the full discussion can be accessed via this link: https://elifesciences.org/articles/67400/peer-reviews#content.

## 5 Conclusion

In this work we provided a detailed analytical description of the mechanisms underlying the emergence of SEs from NOs and BAA co-existing in the human brain. The proposed analytical arguments were tested and confirmed using local and large-scale numerical models together with experimental MEG recordings obtained in healthy subjects during eyes-closed resting state. While previous studies have described SEs within the framework of neuronal avalanches, they often lacked a generative, signal-level account. Here, we bridge that divide by offering a mathematically grounded and empirically validated framework that accounts for oscillatory and aperiodic bursts perspectives on brain activity. We combine experimental evidence supported by a signal-level analytical framework and numerical simulations based on generative models to demonstrate that transient phase-structured alpha bursts, shaped by the SGDC mechanism, contribute to long-range coordination during rest. This extends the communication-through-coherence hypothesis into the transient domain. In summary, our multi-pronged approach, grounded in experimental evidence supported by analytical arguments and extensive model-based validation, enhances the robustness and interpretive depth of our results, offering a more comprehensive picture of how SEs arise from NOs and BAA as fundamental components of MEG activity during resting state.

## Supplementary Material

Supplementary Material

## Data Availability

The MEG data are available upon request to the corresponding author (Pierpaolo Sorrentino), conditional on appropriate ethics approval at the local site. The availability of the data was not previously included in the ethical approval, and, therefore, data cannot be shared directly. In case data are requested, the corresponding author will request an amendment to the local ethical committee. Conditional to approval, the data will be made available. The code and simulated data that support the findings of this study are available from the corresponding author (Damián Dellavale), upon reasonable request. We are willing to provide technical support to investigators who express an interest in implementing the SGDC tools in other programming languages, integrate it in open-source software toolboxes, or use it for non-profit research activities.
