# Supplementary material for: Linking local and large-scale salient events with oscillatory and broadband arrhythmic activities in the resting human brain
Source: Imaging Neurosci (Camb). 2026 Apr 3;4:IMAG.a.1193. doi: 10.1162/IMAG.a.1193 (PMC13051684; doi:10.1162/IMAG.a.1193)
Supplement: Supplementary Material [file IMAG.a.1193_supp.pdf]

Supplementary Material A. Supplementary analytical results

Supplementary Material A.1. Preservation of the Pearson's cross-correlation in the B-surrogates

Let us start by considering the circular cross-correlation $R_{xy}(t')$ between the time series $x(t)$ and $y(t)$ representing the activities of two brain regions (Oppenheim et al., 1999, pp. 571, 746),

$$R_{xy}(t') = \sum_{t=0}^{N_s-1} x^*(t-t')_{\text{mod } N_s}\, y(t) \tag{A.1}$$

where $(x(t),\ y(t)) \in \mathbb{R}$ are finite-length discrete time series having N_s time samples satisfying $(x(t) = 0,\ y(t) = 0)\ \forall\ 0 > t > N_s - 1$, being $t \in \mathbb{Z}$ the discrete time index. By applying the Discrete Fourier Transform (DFT) $\mathfrak{F}\{.\}$ on both sides of Eq. A.1 we obtain (Oppenheim et al., 1999, pp. 575, 746),

$$\begin{aligned} S_{xy}(\omega) &= \mathfrak{F}\{R_{xy}(t')\} \\ &= \mathfrak{F}\{x(t)\}^*\ \mathfrak{F}\{y(t)\} = A_x(\omega)e^{-i\phi_x(\omega)}\ A_y(\omega)e^{i\phi_y(\omega)} \end{aligned} \tag{A.2}$$

where $A_x(\omega)$, $\phi_x(\omega)$ and $A_y(\omega)$, $\phi_y(\omega)$ are the magnitude and phase angle of the DFT spectrum corresponding to the signals $x(t)$ and $y(t)$, respectively. The computation of surrogate time series involves the addition of random phases $\theta(\omega)$ to the corresponding DFT spectra as follows,

$$\begin{aligned} S^s_{xy}(\omega) &= A_x(\omega)e^{-i(\phi_x(\omega)+\theta_x(\omega))}\ A_y(\omega)e^{i(\phi_y(\omega)+\theta_y(\omega))} \\ &= A_x(\omega)e^{-i\phi_x(\omega)}\ A_y(\omega)e^{i\phi_y(\omega)}\ e^{i(\theta_y(\omega)-\theta_x(\omega))} \end{aligned} \tag{A.3}$$

In the A.3, $S^s_{xy}(\omega)$ is the DFT of the circular cross-correlation associated with the surrogated time series $x^s(t) = \mathfrak{F}^{-1}\left\{A_x(\omega)e^{-i(\phi_x(\omega)+\theta_x(\omega))}\right\}$ and $y^s(t) = \mathfrak{F}^{-1}\left\{A_y(\omega)e^{-i(\phi_y(\omega)+\theta_y(\omega))}\right\}$, where $\mathfrak{F}^{-1}\{.\}$ stands for the inverse DFT. In the particular case of the B-surrogates (see Section 2.8 in Methods) we add the same random phase-shift in all the brain regions, that is, $\theta_x(\omega) = \theta_y(\omega)$ producing $e^{i(\theta_y(\omega)-\theta_x(\omega))} = 1$ in the Eq. A.3. Under this condition, the Eqs. A.2 and A.3 becomes equivalent which in turn implies the equivalence between the circular cross-correlations associated with the true data and the B-surrogate,

$$\begin{aligned} S_{xy}(\omega) = S^s_{xy}(\omega) \quad &\Longrightarrow \quad \mathfrak{F}\{R_{xy}(t')\} = \mathfrak{F}\left\{R^s_{xy}(t')\right\} \\ &\Longrightarrow \quad R_{xy}(t') = R^s_{xy}(t') \end{aligned}$$

We confirmed this analytical results by computing the time-averaged functional connectivity as quantified by the pairwise Pearson's correlation on our empirical MEG dataset and the corresponding A- and B-surrogates (see Section 2.8 in Methods). Fig. A.1C shows the matrix resulting from computing the Pearson's correlation on whole time series of the brain regions taken in pairs. Fig. A.1B shows the spatial profile obtained by averaging the Pearson's correlation matrix across rows. Fig. A.1A displays the brain plots corresponding to the

spatial profile of the Pearson's correlation shown in Fig. A.1B. Importantly, Fig. A.1B shows that only B-surrogates reproduce the spatial profile of the Pearson's correlation computed on the MEG data, hence, confirming that the pairwise Pearson's correlation is preserved in the B-surrogates, and not in the case of A-surrogates.

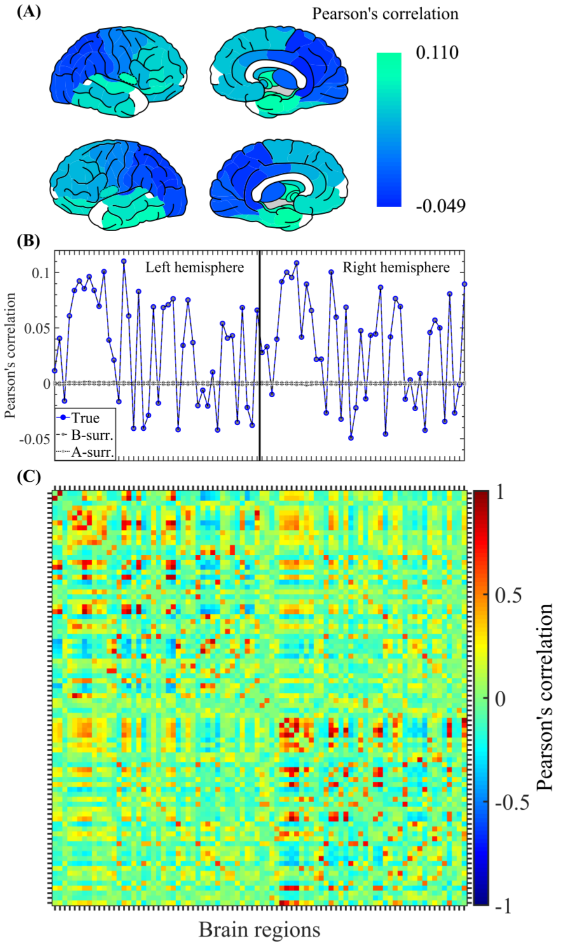

Figure A.1: Pearson's correlation pattern computed on whole time series of the brain regions taken in pairs. (A) Brain topographies corresponding to the Pearson's correlation profile shown in panel B (blue markers). (B) Spatial profile showing the Pearson's correlation (mean value across the 47 participants), i.e., the mean value computed on in each row of the Pearson's correlation matrix. Note that the spatial profiles corresponding to the 100 B-surrogates (dark gray markers) overlap with the spatial profile associated with the true MEG data (blue markers). (C) Pearson's correlation matrix (average across the 47 participants) obtained by computing the Pearson's correlation on the whole time series of the brain regions taken in pairs. In panel B, the labels and ordering of the brain regions are the same as those shown in Fig. C.2.

Supplementary Material A.2. Oscillatory mechanisms underlying the emergence of local above-threshold fluctuations

In this section we provide a detailed description of the mechanism underlying the emergence of local above-threshold fluctuations from the Fourier oscillatory constituents of the brain activity. Our analysis start by projecting the brain signal of interest $x(t)$ onto the Fourier basis functions using the Discrete Fourier Transform (DFT) equations (Oppenheim et al., 1999, Chapters 8 and 10). In doing so we are assuming that $x(t)$ satisfies certain conditions so the resulting spectral estimates exist and are meaningful. Specifically, by considering finite-length time series constituted by N_s time samples, the existence of the DFT representation requires that $x(t)$ is bounded ($|x(t)| < M \in \mathbb{R}\ \forall\ 0 > t > N_s - 1$). Besides, the analyzed brain activity are in general nonstationary, that is, the time series $x(t)$ can be represented as a sum of sinusoidal components with time-varying amplitudes, frequencies, or phases. In this regard, we consider a small enough number of time samples N_s such that the spectral characteristics of the signal $x(t)$ can be assumed stationary during the analyzed time window. Thus, by considering $x(t) \in \mathbb{R}$ being a finite-length discrete time series having an even number of time samples N_s and $x(t) = 0\ \forall\ 0 > t > N_s - 1$, where $t \in \mathbb{Z}$ is the discrete time index. The analysis equation corresponding to the Discrete Fourier Transform (DFT) of $x(t)$ can be written as follows (Oppenheim et al., 1999, p. 561, Eq. (8.67)),

$$X(k) = \sum_{t=0}^{N_s-1} x(t) e^{-i\omega_0 k t} \quad : \quad \omega_0 = \frac{2\pi}{N_s} \tag{A.4}$$

where $k \in \mathbb{Z}$ is the discrete frequency index, in general producing complex Fourier coefficients $X(k) \in \mathbb{C}$ and $X(k) = 0\ \forall\ 0 > k > N_s - 1$. Then, the synthesis equation associated with the inverse DFT (iDFT) is (Oppenheim et al., 1999, p. 561, Eq. (8.68)),

$$x(t) = \frac{1}{N_s} \sum_{k=0}^{N_s-1} X(k) e^{i\omega_0 k t} \tag{A.5}$$

Taking into account that $X(k) = |X(k)| e^{i\phi(k)} \in \mathbb{C}$, the Eq. A.5 can be rewritten as,

$$x(t) = \frac{1}{N_s} \sum_{k=0}^{N_s-1} |X(k)| e^{i(\omega_0 k t + \phi(k))} \tag{A.6}$$

The core of the proposed conceptualization is to note that the Eq. A.6 can be expressed as a sum of (non-overlapping) pairwise adjacent spectral components as follows,

$$\begin{aligned} x(t) &= \frac{1}{N_s} \sum_{k=0}^{N_s/2-1} |X(2k)| e^{i(\omega_0 2kt + \phi(2k))} \\ &+ |X(2k+1)| e^{i(\omega_0 (2k+1)t + \phi(2k+1))} \end{aligned}$$

$$x(t) = \frac{1}{N_s}\sum_{k=0}^{N_s/2-1}\left(|X(2k)|e^{i\phi(2k)} + |X(2k+1)|e^{i(\omega_0 t+\phi(2k+1))}\right)e^{i\omega_0 2kt}$$

By defining the forward phase difference as $\Delta\phi(2k) = \phi(2k+1) - \phi(2k)$, and substituting $\phi(2k+1) = \phi(2k) + \Delta\phi(2k)$ in the previous equation we have,

$$\begin{aligned} x(t) &= \frac{1}{N_s}\sum_{k=0}^{N_s/2-1}\Bigg(|X(2k)|e^{-i\left(\frac{\omega_0}{2}t+\frac{\Delta\phi(2k)}{2}\right)} \\ &+ |X(2k+1)|e^{i\left(\frac{\omega_0}{2}t+\frac{\Delta\phi(2k)}{2}\right)}\Bigg)e^{i\frac{\omega_0}{2}t}e^{i\left(\phi(2k)+\frac{\Delta\phi(2k)}{2}\right)}e^{i\omega_0 2kt} \end{aligned}$$

$$\begin{aligned} x(t) &= \frac{1}{N_s}\sum_{k=0}^{N_s/2-1}\Bigg(|X(2k)|e^{-i\left(\frac{\omega_0}{2}t+\frac{\Delta\phi(2k)}{2}\right)} \\ &+ |X(2k+1)|e^{i\left(\frac{\omega_0}{2}t+\frac{\Delta\phi(2k)}{2}\right)}\Bigg)e^{i\left(\frac{\omega_0}{2}(4k+1)t+\phi(2k)+\frac{\Delta\phi(2k)}{2}\right)} \end{aligned}$$

Then, by introducing in the previous equation the forward frequency difference $\Delta\omega = \omega_0\ (k+1) - \omega_0\ k = \omega_0$, it results,

$$\begin{aligned} x(t) &= \frac{1}{N_s}\sum_{k=0}^{N_s/2-1}\Bigg(|X(2k)|e^{-i\left(\frac{\Delta\omega}{2}t+\frac{\Delta\phi(2k)}{2}\right)} \\ &+ |X(2k+1)|e^{i\left(\frac{\Delta\omega}{2}t+\frac{\Delta\phi(2k)}{2}\right)}\Bigg)e^{i\left(\frac{\Delta\omega}{2}(4k+1)t+\phi(2k)+\frac{\Delta\phi(2k)}{2}\right)} \end{aligned}$$

Taking out $\Delta\omega/2$ as a common factor we have,

$$\begin{aligned} x(t) &= \frac{1}{N_s}\sum_{k=0}^{N_s/2-1}\Bigg(|X(2k)|e^{-i\frac{\Delta\omega}{2}\left(t+\frac{\Delta\phi(2k)}{\Delta\omega}\right)} \\ &+ |X(2k+1)|e^{i\frac{\Delta\omega}{2}\left(t+\frac{\Delta\phi(2k)}{\Delta\omega}\right)}\Bigg)e^{i\left(\frac{\Delta\omega}{2}(4k+1)t+\phi(2k)+\frac{\Delta\phi(2k)}{2}\right)} \end{aligned}$$

The rate of change of the phase with the frequency is associated with the group delay defined as $\tau(k) = -\Delta\phi(k)/\Delta\omega$. Using this definition, the previous equation can be written as,

$$\begin{aligned} x(t) &= \frac{1}{N_s}\sum_{k=0}^{N_s/2-1}\underbrace{\left(|X(2k)|e^{-i\frac{\Delta\omega}{2}(t-\tau(2k))} + |X(2k+1)|e^{i\frac{\Delta\omega}{2}(t-\tau(2k))}\right)}_{\text{Complex envelope (sidebands)}} \\ &\times\ \underbrace{e^{i\left(\frac{\Delta\omega}{2}(4k+1)t+\phi(2k)+\frac{\Delta\phi(2k)}{2}\right)}}_{\text{Complex carrier}} \end{aligned} \tag{A.7}$$

It is essential to note that in Eq. A.7, each (non-overlapping) pair of adjacent spectral components $X(2k), X(2k+1)$ can be interpreted as the sidebands of an amplitude modulated carrier at $(4k+1)\Delta\omega/2$. Importantly, the frequency of the carrier $(4k+1)\Delta\omega/2$ is a function of the frequency index k, that is, it depends on the particular pair of spectral components under consideration $(X(2k), X(2k+1))$. However, the frequency of the modulating component is the same for all the pair of spectral components involved in Eq. A.7, i.e., it is independent of the frequency index k and only determined by the frequency resolution of the DFT as $\Delta\omega/2 = \omega_0/2$ (i.e., half the separation between the two sidebands). Another important characteristic of the representation given by the Eq. A.7 is that the frequencies associated with the complex envelopes $(\Delta\omega/2)$ and with the complex carrier $((4k+1)\Delta\omega/2)$ satisfy the condition $\Delta\omega/2 \leq (4k+1)\Delta\omega/2$. In the telecom theory, a spectral profile satisfying these characteristics is known as the complex baseband representation of a band-limited signal (e.g., amplitude modulated signal) (Oppenheim et al., 1999, Chapter 11.4.2, p. 796; Proakis, 2008, Chapter 2.1, p. 18; Haykin, 2001, Chapter A2.4, p. 725). Accordingly, we refer to the Eq. A.7 as the inverse DFT based on the pairwise complex baseband representation of $x(t)$. In line with this, the Eq. A.7 can be rewritten as a summation of amplitude modulated signals corresponding to each pair of adjacent spectral components as follows,

$$x(t) = \frac{1}{N_s} \sum_{k=0}^{N_s/2-1} x_{k+}(t) \tag{A.8}$$

$$x_{k+}(t) = \tilde{x_k}(t-\tau(k))\, e^{i\left(\frac{\Delta\omega}{2}(4k+1)t+\phi(2k)+\frac{\Delta\phi(2k)}{2}\right)} \tag{A.9}$$

$$\tilde{x_k}(t-\tau(k)) = |X(2k)|e^{-i\frac{\Delta\omega}{2}(t-\tau(2k))} + |X(2k+1)|e^{i\frac{\Delta\omega}{2}(t-\tau(2k))} \tag{A.10}$$

In the Eq. A.8, $x_{k+}(t)$ is the discrete time analytic signal (a.k.a., pre-envelope) corresponding to each amplitude modulated component constituting the original signal $x(t)$, and it is defined in Eq. A.9. In the Eq. A.9, $\tilde{x_k}(t-\tau(k))$ is the complex envelope of each amplitude modulated component constituting the original signal $x(t)$, and it is defined in terms of the spectral components $X(k)$ in the Eq. A.10. It is important to note that the alignment in time of the complex envelopes $\tilde{x_k}(t-\tau(k))$ synthesizing the original signal $x(t)$, via the Eq. A.8, is determined by the group delay $\tau(k)$.

The Eqs. A.7 - A.10 constitute a useful conceptualization linking the DFT and the complex baseband representation to account for the emergence of salient events from the Fourier oscillatory constituents of a band-limited signal. Due to the fact that the analysis proposed above is based on the DFT, in the case of $x(t) \in \mathbb{R}$ the result of the summation in Eqs. A.7 and A.8 is guaranteed to be real valued. At the same time, this also restrict the validity of the analysis to harmonic spectral components $\omega_0\, k$ associated with the fundamental frequency $\omega_0 = 2\pi/N_s$. Now we will present the general equations valid for all the cases, that is, harmonic ($\Delta\omega(k) = \text{cte}$, $\omega(k+1)/\omega(k) \in \mathbb{Q}$), non-harmonic ($\Delta\omega(k) = \text{cte}$, $\omega(k+1)/\omega(k) \in \mathbb{R} \setminus \mathbb{Q}$) and non-uniformly spaced ($\Delta\omega(k) \neq \text{cte}$) Fourier oscillatory components. Let us consider a real valued signal $x(t) \in \mathbb{R}$ resulting

from the linear superposition of an even number N_s of oscillatory components of arbitrary amplitude $A(k)$, frequency $\omega(k)$ and phase $\phi(k)$.

$$x(t) = \sum_{k=0}^{N_s-1} A(k)\ \cos\left(\omega(k)t + \phi(k)\right) : A(k) \in \mathbb{R} \tag{A.11}$$

Since the Eq. A.11 is linear we can introduce the complex notation via the Euler's formula as follows,

$$x(t) = \mathfrak{Re}\left\{ \sum_{k=0}^{N_s-1} A(k)\ e^{i(\omega(k)t+\phi(k))} \right\} \tag{A.12}$$

In the Eq. A.12, the operator $\mathfrak{Re}\{.\}$ stands for "the real part of". By following a similar procedure applied above on the Eq. A.6, the Eq. A.12 can be rewritten as follows,

$$\begin{aligned}
x(t) &= \mathfrak{Re}\left\{ \sum_{k=0}^{N_s/2-1} \left(A(2k)\ e^{-i\left(\frac{\Delta\omega(2k)}{2}t+\frac{\Delta\phi(2k)}{2}\right)} + A(2k+1)\ e^{i\left(\frac{\Delta\omega(2k)}{2}t+\frac{\Delta\phi(2k)}{2}\right)} \right) \right. \\
&\times \left. e^{i\left(\bar{\omega}(2k)t+\bar{\phi}(2k)\right)} \right\} \\
\Delta\phi(2k) &= \phi(2k+1) - \phi(2k) \\
\bar{\phi}(2k) &= \frac{\phi(2k+1) + \phi(2k)}{2} = \phi(2k) + \frac{\Delta\phi(2k)}{2} \\
\Delta\omega(2k) &= \omega(2k+1) - \omega(2k) \\
\bar{\omega}(2k) &= \frac{\omega(2k+1) + \omega(2k)}{2} = \omega(2k) + \frac{\Delta\omega(2k)}{2}
\end{aligned}$$

In this case the group delay is defined as $\tau(k) = -\frac{\Delta\phi(k)}{\Delta\omega(k)}$, thus, the previous equation results,

$$\begin{aligned}
x(t) &= \mathfrak{Re}\left\{ \sum_{k=0}^{N_s/2-1} \underbrace{\left(A(2k)\ e^{-i\frac{\Delta\omega(2k)}{2}(t-\tau(2k))} + A(2k+1)\ e^{i\frac{\Delta\omega(2k)}{2}(t-\tau(2k))} \right)}_{\text{Complex envelope (sidebands)}} \right. \\
&\times \left. \underbrace{e^{i\left(\bar{\omega}(2k)t+\bar{\phi}(2k)\right)}}_{\text{Complex carrier}} \right\}
\end{aligned} \tag{A.13}$$

The Eq. A.13 is the pairwise complex baseband representation of the signal $x(t)$. Provided that the frequencies associated with the complex envelopes $(\Delta\omega(2k)/2)$ and the complex carrier $(\bar{\omega}(2k))$ satisfy the condition $\Delta\omega(2k)/2 < \bar{\omega}(2k)$, the Eq. A.13 can also be written as a summation of discrete time analytic signals $x_{k+}(t)$ associated with amplitude modulated signals corresponding to each pair

of adjacent oscillatory components as follows,

$$x(t) = \mathfrak{Re}\left\{ \sum_{k=0}^{N_s/2-1} x_{k+}(t) \right\} \tag{A.14}$$

$$x_{k+}(t) = \tilde{x_k}(t-\tau(k))\; e^{i\left(\bar{\omega}(2k)t+\bar{\phi}(2k)\right)} \tag{A.15}$$

$$\begin{aligned} \tilde{x_k}(t-\tau(k)) &= A(2k)\; e^{-i\frac{\Delta\omega(2k)}{2}(t-\tau(2k))} \\ &+ A(2k+1)\; e^{i\frac{\Delta\omega(2k)}{2}(t-\tau(2k))} \end{aligned} \tag{A.16}$$

Similarly to the previous case the time alignment of the complex envelopes $\tilde{x_k}(t-\tau(k))$ synthesizing the original signal $x(t)$, via the Eq. A.14, is determined by the group delay $\tau(k)$.

In what follows we will use the Eq. A.13 to illustrate the role of the group delay in the emergence of above-threshold fluctuations from the oscillatory constituents of the synthetic signal $x(t)$. As a first example, let us consider a spectral profile given by a set of constant-amplitude $A(k) = A = 1$ oscillatory components uniformly spaced $f_s\;\Delta\omega/(2\pi) = 1.2/\sqrt{2}$ Hz and having non-harmonic frequencies $f_s\;\omega(k)/(2\pi) = 0.5 + k\; f_s\;\Delta\omega/(2\pi) \in [0.5-5]$ Hz, where $f_s = 1024$ Hz is the sampling rate (see Figs. A.2A and A.2F). Accordingly, the Eq. A.13 becomes,

$$x(t) = A\;\mathfrak{Re}\left\{ \sum_{k=0}^{N_s/2-1} \left(e^{-i\frac{\Delta\omega}{2}(t-\tau(2k))} + e^{i\frac{\Delta\omega}{2}(t-\tau(2k))} \right) e^{i\left(\bar{\omega}(2k)t+\bar{\phi}(2k)\right)} \right\}$$

By using the Euler's formula to rearrange the modulating factor, the previous equation results,

$$\begin{aligned} x(t) &= \frac{A}{2}\;\mathfrak{Re}\left\{ \sum_{k=0}^{N_s/2-1} \cos\left(\frac{\Delta\omega}{2}(t-\tau(2k))\right) e^{i\left(\bar{\omega}(2k)t+\bar{\phi}(2k)\right)} \right\} \\ &= \frac{A}{2} \sum_{k=0}^{N_s/2-1} \underbrace{\cos\left(\frac{\Delta\omega}{2}(t-\tau(2k))\right)}_{\text{Modulating component}} \underbrace{\cos\left(\bar{\omega}(2k)t+\bar{\phi}(2k)\right)}_{\text{Modulated component}} \end{aligned} \tag{A.17}$$

The Eq. A.17 explicitly shows that any pair of adjacent oscillatory components associated with the signal $x(t)$ can be interpreted as an amplitude modulated signal with the same modulating function $\cos\left(\frac{\Delta\omega}{2}(t-\tau(2k))\right)$. The key concept here is to note that, when all the oscillatory components in Eq. A.17 are added together to synthesize the signal $x(t)$ in the time-domain, the group delay τ will determine the time alignment of the modulating functions associated with each pair of adjacent oscillatory components. As a consequence, in the case of all the spectral components $A(k)\; e^{i(\omega(k)t+\phi(k))}$ in Eq. A.12 having constant phase produces $\Delta\phi = 0 \implies \tau = -\Delta\phi/\Delta\omega = 0$, hence, all the modulating functions $\cos\left(\frac{\Delta\omega}{2}(t-0)\right)$ in Eq. A.17 will be aligned in time (at

$t = 0$) giving rise to a sinc-like function representing the maximum amplitude excursion (i.e., a salient event) that can be elicited by the set of Fourier oscillatory components constituting the Eq. A.12. In the case of all the spectral components in Eq. A.12 having a phase proportional to the discrete frequency index $\phi(k) = -\tau_0\ \Delta\omega\ k \implies \Delta\phi(k) = -\tau_0\ \Delta\omega$, results in a group delay which does not dependent on the frequency $\tau(k) = -\Delta\phi(k)/\Delta\omega = \tau_0$, thus, in Eq. A.12 we obtain a modulating component $\cos\left(\frac{\Delta\omega}{2}(t - \tau_0)\right)$. That is, all the modulating functions will again be aligned in time producing the same salient event given by the sinc-like function as in the previous case but this time centered at $t = \tau_0$ (i.e., a time-shift, see Figs. A.2A-E). On the other hand, in the case of the phases associated with the spectral components in Eq. A.12 having a non-linear dependence with the discrete frequency index, e.g., $\phi(k) = -\tau_0\ \Delta\omega\ k^2 \implies \Delta\phi(k) = -\tau_0\ \Delta\omega(2k + 1)$, the group delay results a function of the frequency $\tau(k) = \tau_0(2k+1)$, hence, preventing the alignment in time of the modulating functions associated with each pair of adjacent spectral components $\cos\left(\frac{\Delta\omega}{2}(t - \tau(k))\right)$. In this case, the signal $x(t)$ discloses sub-threshold excursions of amplitude (see Figs. A.2F-J). It is worth mentioning that in deriving the pairwise complex baseband representation of $x(t)$ given by the Eqs. A.7 and A.13, we grouped the original spectral components (Eqs. A.5 and A.11) in subsets of (non-overlapping) pairs adjacent in frequency. The strategy of grouping the spectral components in subsets is necessary to obtain a representation based on a sum of complex envelopes modulating the complex carriers. Representations similar to those presented in the Eqs. A.7 and A.13 can be obtained by defining subsets containing more than 2 non-overlapping spectral components (not necessarily adjacent in frequency). However, our approach based on grouping adjacent spectral components in non-overlapping pairs discloses the following relevant features:

1 By defining subsets of 2 spectral components, we obtain the simplest complex envelopes characterized by a cos- or sin-like waveform shape (see the modulating component in the Eq. A.17 and the colored solid lines in Figs.A.2E and A.3E).

2 By defining pairs of spectral components adjacent in frequency, we maximize the waveform shape similarity among the resulting complex envelopes. In the case of uniformly spaced spectral components ($\Delta\omega = \text{cte}$), we obtain complex envelopes having the same time period $2/\Delta\omega$ (see the colored doted lines in Figs.A.2E and A.3E).

3 By defining pairs of spectral components adjacent in frequency, we also maximize the similarity among the resulting complex carriers (see the colored solid lines in Figs.A.2E and A.3E).

Taking together, these features are of particular importance to support the link between the spectral group delay consistency (SGDC) defining the time alignment of the modulating components (complex envelopes) with the constructive interference of the modulated components (complex carriers), which

in turn lead to the occurrence of salient events. As a conclusion, the results described above in connection with the Eqs. A.7, A.13, show that the emergence of above-threshold fluctuations in the signal $x(t)$ is related to the consistency of the group delay $\tau(k)$ across the discrete frequency values k. That is, the occurrence of salient events is supported by a slowly varying group delay as a function of the frequency, and this hold true for harmonic, non-harmonic and also for non-uniformly spaced Fourier oscillatory constituents of the signal under analysis.

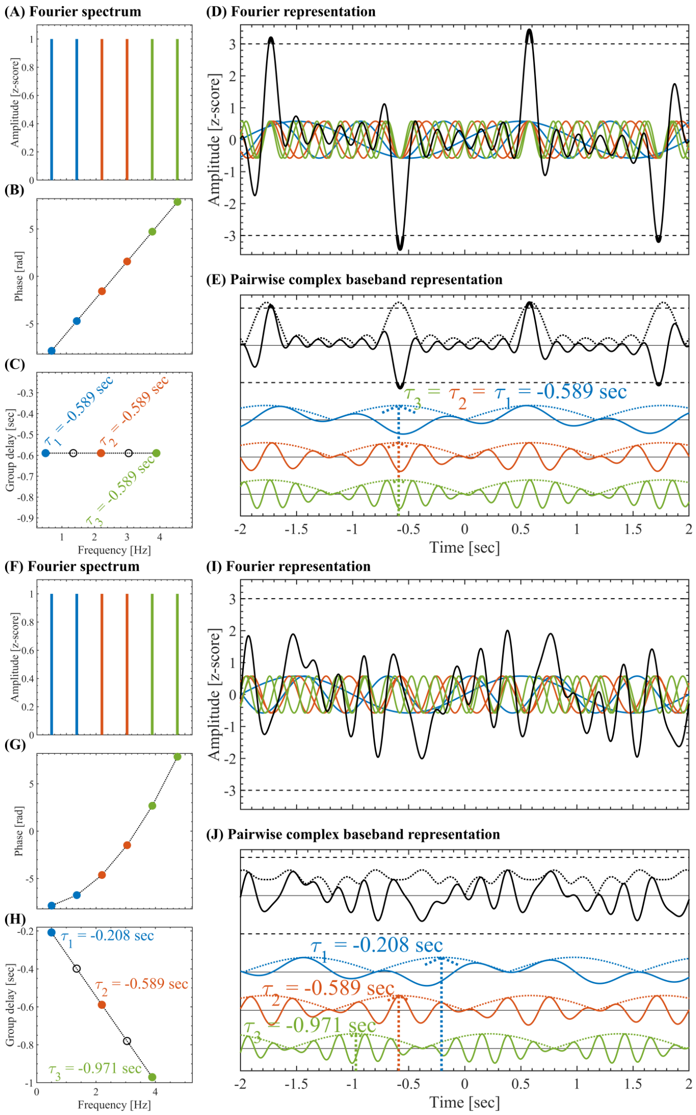

Figure A.2: Pairwise complex baseband representation for a set of oscillatory components with A_k = cte. (A) Set of constant-amplitude $A(k) = 1$ oscillatory components uniformly spaced ($f_s\ \Delta\omega/(2\pi) = 1.2/\sqrt{2}$ Hz) and having non-harmonic frequencies $f_s\ \omega(k)/(2\pi) = 0.5 + k\ f_s\ \Delta\omega/(2\pi) \in [0.5 - 5]$ Hz, where $f_s = 1024$ Hz is the sampling rate. The pairwise complex baseband representation (Eq. A.13) was obtained by grouping the oscillatory components in adjacent non-overlapping pairs color-coded in blue, red and green. (B) Phases $\phi(k)$ having a linear dependence as a function of the frequency within the range $\phi(k) \in 2.5\ [-\pi, \pi]$. (C) Group delay $\tau(k)/f_s = -\Delta\phi(k)/(f_s\ \Delta\omega)$ for the pairs of adjacent oscillatory components. The color-coded filled markers correspond to the $\tau(2k)/f_s$ values, and the black empty markers correspond to $\tau(2k+1)/f_s$ values (see Eq. A.13). (D) Z-scored signals. The solid color-coded lines represent the individual oscillatory components, the solid black line is the resulting signal $x(t)$, the horizontal dashed black lines indicate the threshold at $|z| = 3$. (E) Pairwise complex baseband representation. The solid color-coded lines represent the individual amplitude modulated signals (pairs of adjacent oscillatory components), the solid black line is the resulting signal $x(t)$, the color-coded and black doted lines are the corresponding amplitude envelopes. (F - J) Same as panels (A - E), this time with phases $\phi(k)$ having a quadratic dependence as a function of the frequency within the range $\phi(k) \in 2.5\ [-\pi, \pi]$ (see panel G).

The group delay is defined in terms of the rate of change of the phase with the frequency, being independent on the amplitude of the spectral components. As a consequence, the consistency of the spectral group delay as a mechanism supporting the emergence of salient events is also valid for spectral profiles other than the constant-amplitude spectrum shown in the Fig. A.2. The Fig. A.3 shows the results for a spectral profile given by a set of (uniformly spaced) non-harmonic oscillatory components with amplitudes $A(k) \propto 1/\sqrt{k}$, that is, the power of the spectral components $A^2(k)$ is proportional to $1/k$ (see Figs. A.3A and A.3F). Figs. A.3A-E show the case in which the phases $\phi(k)$ of the spectral components $A(k)\ e^{i(\omega(k)t+\phi(k))}$ in Eq. A.12 are randomly distributed in a very small range around zero ($\phi(k) \in [-\pi/10, \pi/10]$). Under this condition, the pairwise complex baseband representation (Eq. A.13) shown in the Fig. A.3E is constituted by amplitude modulated signals highly aligned in time. As a consequence, prominent salient events can be distinguished in the resulting signal (see solid black line in panels D and E of Fig. A.3). On the other hand, Figs. A.3A-E show the case in which the phase values $\phi(k)$ are randomly distributed in a wider range $\phi(k) \in [-\pi, \pi]$. Under this condition, the pairwise complex baseband representation (Eq. A.13) shown in the Fig. A.3J is constituted by amplitude modulated signals non-aligned in time. As a consequence, the resulting signal $x(t)$ only discloses sub-threshold excursions of amplitude (see solid black line in panels I and J of Fig. A.3).

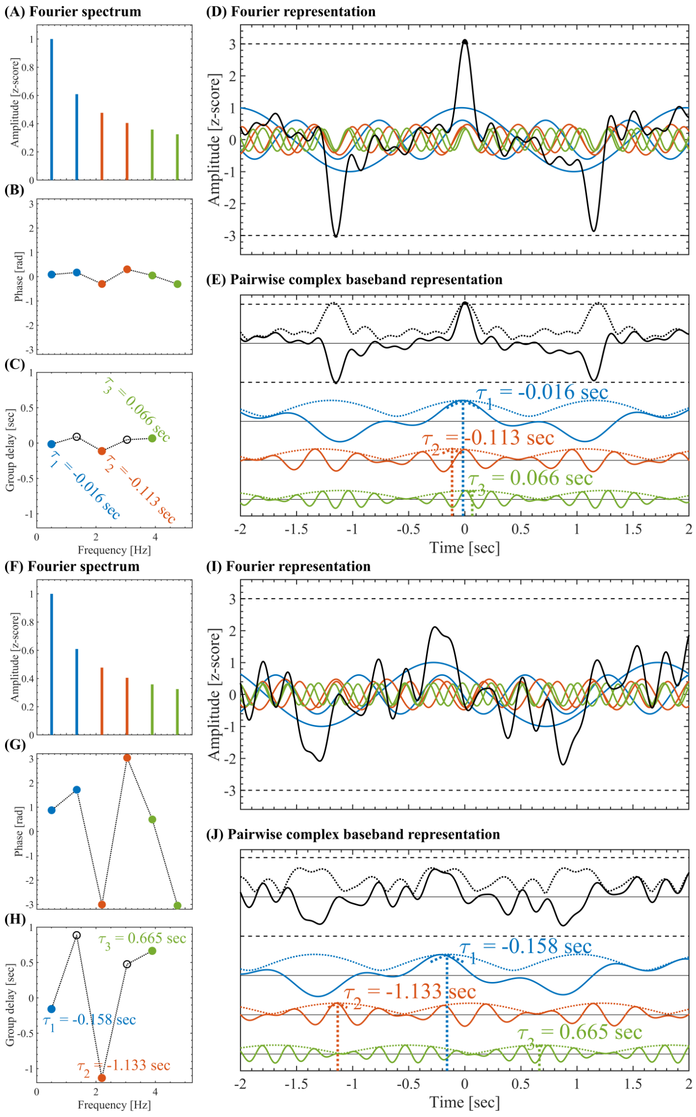

Figure A.3: Pairwise complex baseband representation for a set of oscillatory components with $A(k) \propto 1/\sqrt{k}$. (A) Set of non-constant amplitude $A(k) \propto 1/\sqrt{k}$ oscillatory components uniformly spaced ($f_s\ \Delta\omega/(2\pi) = 1.2/\sqrt{2}$ Hz) and having non-harmonic frequencies $f_s\ \omega(k)/(2\pi) = 0.5 + k\ f_s\ \Delta\omega/(2\pi) \in [0.5 - 5]$ Hz, where $f_s = 1024$ Hz is the sampling rate. The pairwise complex baseband representation (Eq. A.13) was obtained by grouping the oscillatory components in adjacent non-overlapping pairs color-coded in blue, red and green. (B) Phases $\phi(k)$ randomly distributed within a very small range around zero ($\phi(k) \in [-\pi/10, \pi/10]$). (C) Group delay $\tau(k)/f_s = -\Delta\phi(k)/(f_s\ \Delta\omega)$ for the pairs of adjacent oscillatory components. The color-coded filled markers correspond to the $\tau(2k)/f_s$ values, and the black empty markers correspond to $\tau(2k+1)/f_s$ values (see Eq. A.13). (D) Z-scored signals. The solid color-coded lines represent the individual oscillatory components, the solid black line is the resulting signal $x(t)$, the horizontal dashed black lines indicate the threshold at $|z| = 3$. (E) Pairwise complex baseband representation. The solid color-coded lines represent the individual amplitude modulated signals (pairs of adjacent oscillatory components), the solid black line is the resulting signal $x(t)$, the color-coded and black doted lines are the corresponding amplitude envelopes. (F - J) Same as panels (A - E), this time the phases $\phi(k)$ are randomly distributed within the range $\phi(k) \in [-\pi, \pi]$ (see panel G).

In summary, the analytical arguments presented above, condensed in the Eqs. A.7 - A.10 and A.13 - A.16, allowed us to identify the consistency of the group delay across the spectral components as a mechanism accounting for the emergence of above-threshold fluctuations from the Fourier oscillatory constituents of the activity associated with a single brain region. In the next section we describe the signal processing tools proposed to quantify the SGDC in empirical data.

Supplementary Material A.3. Measures to assess the spectral group delay consistency

The analytical arguments presented in the Supplementary Material A.2 have profound consequences regarding the interpretation of the experimental results in connection with the emergence of salient events from NOs and broadband $1/f$ activity. Specifically, the pairwise complex baseband representation of band-limited signals (Eqs. A.7 - A.10 and A.13 - A.16), explicitly shows that the mechanism underlying the emergence of above-threshold fluctuations in a signal $x(t)$ can be understood in terms of the consistency of the group delay across the Fourier oscillatory constituents of the signal (see the complex envelopes $\tilde{x_k}(t-\tau(k))$ in Eqs. A.10 and A.16). By considering a multi-regional approach, the pairwise complex baseband representation can be applied on the activity $x_r(t)$ of each brain region r, to obtain complex envelopes of the form $\tilde{x}_{r,k}(t-\tau_r(k))$. Here we recall that $\tilde{x}_{r,k}(t-\tau_r(k))$ determine the envelopes of the individual amplitude modulated signals constituting the signal $x_r(t)$ (see the solid and doted color-coded curves in the Figs. A.2E,J and A.3E,J). Hence, the consistency of the spectral group delay $\tau_r(k)$ determines the synchronization of the complex envelopes $\tilde{x}_{r,k}(t-\tau_r(k))$ across both frequency values $\omega(k)$ and brain regions r. In what follows we describe the proposed measures designed to quantify the spectral group delay consistency (SGDC) in experimental data across either frequency values and/or brain regions. In order to simplify the notation, in the rest of this section we will use ω instead of the discrete frequency index k, implicitly assuming that $\omega=\omega(k)$. In the most general case, the spectral group delay can be estimated as $\tau_r(\omega)=-\Delta\phi_r(\omega)/\Delta\omega(\omega)$, where $\Delta\phi_r(\omega)$ and $\Delta\omega(\omega)$ are the incremental phase and incremental frequency between adjacent spectral components associated with the activity $x_r(t)$ of the brain region r, respectively. Let us consider first the particular case of $\Delta\omega(\omega)=\Delta\omega=\text{const}$, in which the group delay results $\tau_r(\omega)\propto-\Delta\phi_r(\omega)$. Therefore, the SGDC can be simply assessed via the Euler's transform of the incremental phase as follows,

$$SGDC(r) = \frac{1}{N}\sum_{\omega} e^{-i\Delta\phi_r(\omega)} : \Delta\omega = \text{const across } r \quad (A.18)$$

$$SGDC(\omega) = \frac{1}{N}\sum_{r} e^{-i\Delta\phi_r(\omega)} : \Delta\omega = \text{const across } \omega \quad (A.19)$$

The modulus of Eqs. A.18 and A.19 satisfies,

$$|SGDC| = \left|\frac{1}{N}\sum e^{-i\Delta\phi_r(\omega)}\right| = R = (1-S) \in [0,1] \quad (A.20)$$

In the Eqs. A.18, A.19 and A.20, N is the number of either frequency values or brain regions as appropriate, R is the resultant vector length and S is the circular variance (Berens, 2009). The Eq. A.20 explicitly shows that the SGDC is assessed as one minus the circular variance of the incremental phase. The definition of the SGDC measures given in the Eqs. A.18, A.19 and A.20 should not be confused with the traditional measure for quantifying coherence known as Phase Locking Value (PLV) (Tass et al., 1998; Lachaux et al., 1999). Specifically, the SGDC measures as defined in the Eqs. A.18, A.19 and A.20 assess the consistency of the incremental phase $\Delta\phi_r(\omega)$ across the frequency values ω. In contrast, the PLV assesses the consistency of phase difference across the time samples, where the phase difference is computed between two phase time series corresponding to two specific frequency bands in the same or different brain regions (Tass et al., 1998; Lachaux et al., 1999). As stated in the Eq. A.18, the $SGDC(r)$ is a bounded measure in the range $[0, 1]$ and quantifies how much the group delay varies across the spectral components conforming the activity of interest $x_r(t)$. On the one hand, constant group delay values $\tau_r(\omega) \propto -\Delta\phi_r(\omega)$ across the spectral components produce $|SGDC(r)| \approx 1$ indicating a high SGDC, which is associated with high burstiness of the signal $x_r(t)$ (see Figs. A.2A-E and A.3A-E). On the other hand, in the case of group delay values varying randomly (or non-linearly) across the spectral components produces $|SGDC(r)| \approx 0$ indicating low SGDC associated with low burstiness of the signal $x_r(t)$ (see Figs. A.2F-J and A.3F-J). Similarly, the $SGDC(\omega)$ defined in the Eq. A.19 is a bounded measure in the range $[0, 1]$ and quantifies how much the spectral group delay at a given frequency ω, varies across the brain regions r. On the one hand, constant group delay values $\tau_r(\omega) \propto -\Delta\phi_r(\omega)$ across the brain regions produce $|SGDC(\omega)| \approx 1$ indicating a high group delay consistency, which is associated with high cross-regional synchronization of the bursts at a given frequency ω. On the other hand, in the case of group delay values varying randomly (or non-linearly) across the brain regions produces $|SGDC(\omega)| \approx 0$ indicating low group delay consistency associated with low cross-regional synchronization of the bursts at a given frequency ω. Now we will consider the more general case in which $\Delta\omega(k) \neq$ cte. In line with the previous analysis, the SGDC measures can be defined in terms of the linear variance of the group delay $\mathrm{Var}(\tau)$ as follows,

$$|SGDC| = 1 - \frac{\mathrm{Var}(\tau)}{\max\{\mathrm{Var}(\tau)\}} \in [0, 1] \tag{A.21}$$

$$\mathrm{Var}(\tau) = \frac{1}{N} \sum (\tau - \langle\tau\rangle)^2 \tag{A.22}$$

In the Eq. A.22, the mean group delay value $\langle\tau\rangle$ and the the sum associated with the linear variance $\mathrm{Var}(\tau)$ are computed across the N frequency values ω or brain regions r in which case the Eq. A.21 produces $|SGDC(r)|$ or $|SGDC(\omega)|$, respectively. Importantly, the Eqs. A.18, A.19 and A.21 constitute an specialized framework to quantify the emergence of large-scale bursts (i.e., salient network events) from the brain activity. That is, the $SGDC(r)$ assesses the emergence of local above-threshold fluctuations from the spectral components constituting

the activity of a single brain region, whereas the $SGDC(\omega)$ measure quantifies the synchronization of the above-threshold bursts across brain regions. In line with this, we introduce the pairwise spectral group delay consistency (pSGDC) to quantify the burstiness and cross-regional bursts synchronization in a single measure. In the case of $\Delta\omega(\omega) = \Delta\omega = \text{const}$, the pSGDC is defined as follows,

$$\begin{aligned} &pSGDC(r_1, r_2) = \\ &\underbrace{\left(\frac{SGDC(r_1) + SGDC(r_2)}{2}\right)}_{\text{Mean pairwise burstiness}} \underbrace{\frac{1}{N}\sum_{\omega} e^{-i(\Delta\phi_1(\omega) - \Delta\phi_2(\omega))}}_{\text{Correlation of burstiness across } \omega} \\ &: \Delta\omega = \text{const across } r \end{aligned} \tag{A.23}$$

In the Eq. A.23, the quantities $SGDC(r_1)$ and $SGDC(r_2)$ are computed using the Eq. A.18. In the case of $\Delta\omega(\omega) \neq \text{cte}$ the pSGDC is defined as follows,

$$\begin{aligned} &pSGDC(r_1, r_2) = \\ &\underbrace{\left(\frac{SGDC(r_1) + SGDC(r_2)}{2}\right)}_{\text{Mean pairwise burstiness}} \underbrace{\frac{\text{Cov}(\tau_1(\omega), \tau_2(\omega))}{\text{Var}(\tau_1(\omega))\ \text{Var}(\tau_2(\omega))}}_{\text{Correlation of burstiness across } \omega} \\ &\text{Cov}(\tau_1(\omega), \tau_2(\omega)) = \frac{1}{N}\sum_{\omega} \left(\tau_1(\omega) - \langle \tau_1(\omega) \rangle\right)\left(\tau_2(\omega) - \langle \tau_2(\omega) \rangle\right) \end{aligned} \tag{A.24}$$

In the Eq. A.24, the quantities $SGDC(r_1)$ and $SGDC(r_2)$ are computed using the Eqs. A.21 and A.22. Besides, the quantities $\text{Var}(\tau_1(\omega))$ and $\text{Var}(\tau_2(\omega))$ are computed using the Eq. A.22. In both cases the sum associated with the Eq. A.22 is computed over the frequency values ω. The Eqs. A.23 and A.24 show that the $pSGDC(r_1, r_2)$ is a linear measure conformed by a factor quantifying the cross-regional correlation between the group delays across the frequency values, weighted by a coefficient quantifying the burstiness of the two involved brain regions (r_1, r_2). Importantly, we found that the pSGDC performs similarly to the cokurtosis (fourth standardized cross central moment) (Hindriks and Tewarie, 2023) in reproducing the observed salient events topographies and co-activation patterns (see Fig. 7 in Section 3.6 of the main text). This is particularly interesting taking into account that these two non-time-resolved measures (i.e., computed on the whole time series) effectively reproduce the salient events topographies through two different approaches. That is, the cokurtosis is a non-linear time-domain measure, whereas the pSGDC is a linear measure entirely based on the frequency-domain. Moreover, the pSGDC and cokurtosis disclose a better performance to reproduce the observed salient events topographies and co-activation patterns when compared to the kurtosis (scaled version of the fourth central moment) and the Pearson's linear correlation (see discussion in Section 3.6 of the main text). These results are consistent with the fact that kurtosis measures the presence of outliers (tails of the distribution of amplitude values) and the Pearson's correlation coefficient the linear correlations between the two time series. On the other hand, pSGDC and cokurtosis measures quantify these two features simultaneously. In this work the kurtosis (K) and the

cokurtosis (CK) were assessed via the following standard unbiased estimators,

$$
\begin{aligned}
K(r) &= \frac{(N_s-1)}{(N_s-2)(N_s-3)}\big((N_s+1)k(r)-3(N_s-1)\big) \qquad (A.25)\\
k(r) &= \frac{\mu_{r,4}}{\sigma_r^4}\\
\mu_{r,4} &= \frac{1}{N_s}\sum_t \big(x_r-\langle x_r\rangle\big)^4\\
\sigma_r^4 &= \left(\frac{1}{N_s}\sum_t \big(x_r-\langle x_r\rangle\big)^2\right)^2
\end{aligned}
$$

$$
\begin{aligned}
CK(r_1,r_2) &= \frac{ck(r_1,r_2)}{\sigma_1^2\sigma_2^2} \qquad (A.26)\\
ck(r_1,r_2) &= \frac{1}{N_s}\sum_t \big(x_1-\langle x_1\rangle\big)^2\big(x_2-\langle x_2\rangle\big)^2\\
\sigma_r^2 &= \frac{1}{N_s}\sum_t \big(x_r-\langle x_r\rangle\big)^2
\end{aligned}
$$

In the Eqs. A.25 and A.26, N_s is the number of time samples and $\langle . \rangle$ stands for mean value across the time samples.

In the rest of this section, we present illustrative examples using the Eqs. A.18 and A.19 on synthetic multi-channel bursts emerging from narrowband oscillatory activity. Fig. A.4 shows the $|SGDC(r)|$ computed using the Eq. A.18 for three time series synthesized using the Eq. A.11. In each channel, the signal was synthesized by the linear superposition of 10 sinusoidal tones with uniformly spaced frequencies ($\Delta\omega = \text{const}$) in the range $f_s\ \omega/(2\pi) \in [0.5-3]$ Hz. In channels 1 and 2, the phase of the tones were set as a quadratic function of the frequency within the range $\phi_1(\omega) \propto 2\pi\omega^2 \in [-2\pi, 2\pi]$ and $\phi_2(\omega) \propto \pi\omega^2 \in [-\pi, \pi]$, respectively. In channel 3, the phase of the tones were set as a linear function of the frequency within the range $\phi_3(\omega) \propto \pi\omega \in [-\pi, \pi]$. Fig. A.4B shows that the higher the burstiness (i.e., amplitude of the transient fluctuations) disclosed by the resulting signal (see solid black line in the Fig. A.4A), the higher the $|SGDC(r)|$ value. The channel 3, corresponding to the tones having a linear phase dependence with the frequency, discloses the maximum $|SGDC(r)| \approx 1$.

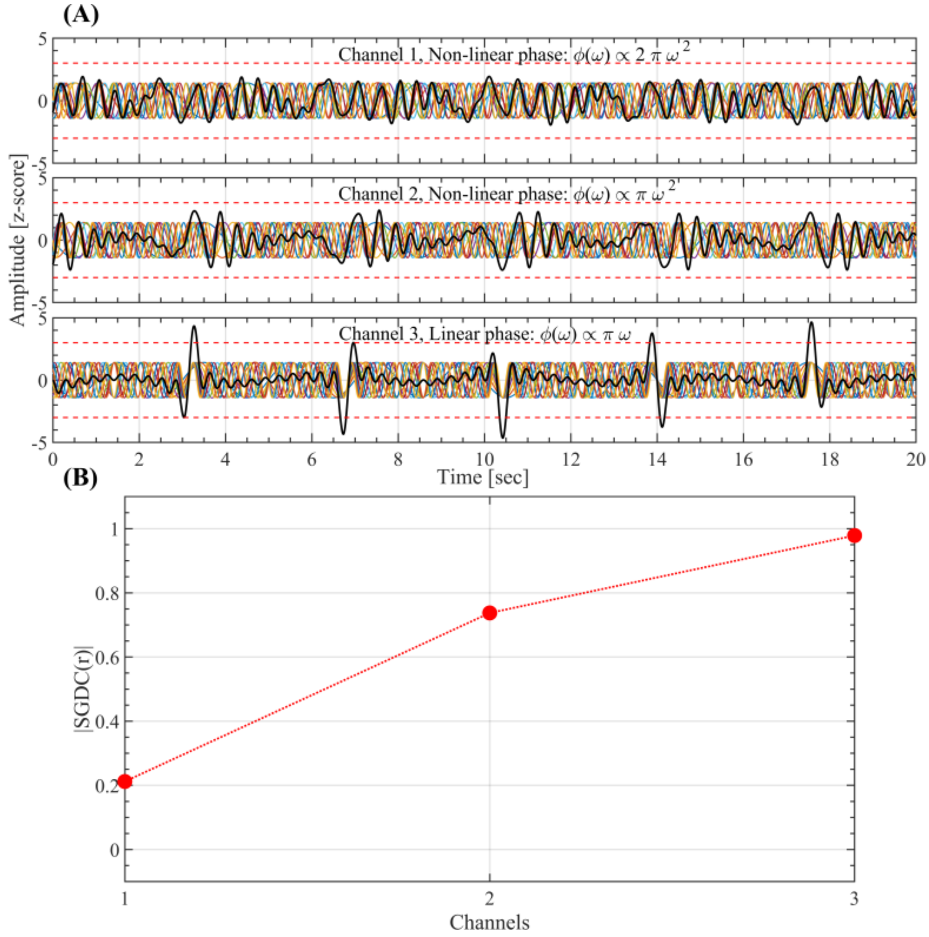

Figure A.4: $SGDC(r)$ computed using the Eq. A.18 on a multi-channel configuration. (A) Three time series $x_r(t)$ (black solid lines) synthesized using the Eq. A.11. In each channel, the signal $x_r(t)$ was synthesized by the linear superposition of 10 sinusoidal tones (colored solid lines) with unitary amplitude and uniformly spaced frequencies ($f_s\ \Delta\omega/(2\pi) = 0.278$ Hz) within the range $f_s\ \omega(k)/(2\pi) = 0.5 + k\ f_s\ \Delta\omega/(2\pi) \in [0.5 - 3]$ Hz. In the channels 1 and 2, the phase of the tones were set as a quadratic function of the frequency within the range $\phi_1(\omega) \propto 2\pi\omega^2 \in [-2\pi, 2\pi]$ and $\phi_2(\omega) \propto \pi\omega^2 \in [-\pi, \pi]$, respectively. In the channel 3, the phase of the tones were set as a linear function of the frequency within the range $\phi_3(\omega) \propto \pi\omega \in [-\pi, \pi]$. (B) Modulus of the $SGDC(r)$ for each channel. Note that the higher the burstiness (i.e., amplitude of the transient fluctuations) disclosed by the resulting signal (see solid black line in the panel A), the higher the $|SGDC(r)|$ value. As expected, the channel 3 corresponding to the tones having a linear phase dependence with the frequency discloses the maximum $|SGDC(r)| \approx 1$.

Figs. A.5 and A.6 show the $SGDC(\omega)$ computed using the Eq. A.19 compared against the Phase Locking Value (PLV) assessed using the following ex-

pression (Tass et al., 1998; Lachaux et al., 1999),

$$PLV = \frac{1}{N} \sum_t e^{i(n\psi(t) - m\theta(t))} \tag{A.27}$$

In Eq. A.27, $\psi(t)$ and $\theta(t)$ are the phase time series of interest and the integers $n, m \in \mathbb{N}$ are required to allow the comparison of phase time series pertaining to different frequency bands. Of note, the $SGDC(\omega)$ quantifies, at each frequency value, the bursts synchronization across the brain regions (channels), whereas the PLV quantifies either local or cross-regional phase coherence between two frequency bands and it is not related to the signal burstiness, i.e., the PLV is not sensitive to the emergence of above-threshold fluctuations neither to the cross-regional synchronization of salient events. Fig. A.5A shows three channels in which the resulting time series (solid black line) have been synthesized as the linear superposition of 10 sinusoidal tones with uniformly spaced frequencies ($\Delta\omega$ = const) in the range $f_s\ \omega/(2\pi) \in [0.5 - 3]$ Hz (see Eq. A.11). In each channel, the phase of all the oscillatory components was set to zero ($\phi_r(\omega) = 0\ \forall\ \omega$). The local and cross-regional effects of this setup can be summarized as follows,

- In each channel (local effect), we obtain the maximum group delay consistency across frequency values accounting for the emergence of above-threshold fluctuations. That is, $\phi_r(\omega) = 0 \implies \Delta\phi_r(\omega) = 0 \implies \tau_r(\omega) = -\Delta\phi_r/\Delta\omega = 0 = \text{cte} \implies SGDC(r) = 1 : r = 1, 2, 3$ (data not shown).
- At each frequency, we obtain the maximum group delay consistency across channels (cross-regional effect) accounting for the synchronization of the salient events across the channels. That is, $\phi_r(\omega) = 0 \implies \Delta\phi_r(\omega) = 0 \implies \tau_r(\omega) = -\Delta\phi_r/\Delta\omega = 0 = \text{cte} \implies SGDC(\omega) = 1\ \forall\ \omega$. The resulting $|SGDC(\omega)|$ is shown in Fig. A.5B.
- At each frequency, we obtain the maximum phase coherence across channels (cross-regional effect). That is, $\psi_{r,\omega}(t) - \theta_{r',\omega}(t) = 0 \implies |PLV| = 1\ \forall\ \omega$, where the phase time series $\psi_{r,\omega}(t)$ and $\theta_{r',\omega}(t)$ were extracted from different channels ($(r, r') \in \{1, 2, 3\} : r \neq r'$) and evaluated at the same frequency ω. In other words, $\psi_{r,\omega}(t)$ and $\theta_{r',\omega}(t)$ are the phase time series associated with two tones homologous in frequency and pertaining to different channels. The resulting $|PLV|$ is shown in the Fig. A.5B.

Fig. A.5C shows three time series constituted by the same 10 tones used in Fig. A.5A, with the difference that in this case the phase of the tones were set as $\phi_1(\omega) = 0$, $\phi_2(\omega) \propto -3\pi\omega$ and $\phi_3(\omega) \propto +3\pi\omega$ for the channel 1, 2 and 3, respectively. The linear phase dependence with the frequency associated with the channels 2 and 3 produces a time-shift in the resulting signals. As a consequence, in this multi-channel configuration the resulting above-threshold fluctuations are not synchronized across channels (see the solid black lines in the Fig. A.5C). In this case, the SGDC and PLV measures result,

- In each channel (local effect), we obtain the maximum group delay consistency across frequency values accounting for the emergence of above-threshold fluctuations. That is, $\Delta\phi_r(\omega) = \text{const} \implies \tau_r(\omega) = -\Delta\phi_r/\Delta\omega = \text{cte} \implies SGDC(r) = 1 : r = 1, 2, 3$. Note that this result is similar to what we obtained for a constant group delay (i.e., not a function of the frequency) associated with the channel 3 shown in Fig. A.4.

- At each frequency, we obtain a low group delay consistency across channels (cross-regional effect) accounting for the lack synchronization of the salient events across the channels. That is, $\Delta\phi_1(\omega) = 0, \Delta\phi_2(\omega) < 0, \Delta\phi_3(\omega) > 0 \implies \tau_1(\omega) = 0, \tau_2(\omega) > 0, \tau_3(\omega) < 0 \implies SGDC(\omega) \approx 0 \ \forall\ \omega$. The resulting $|SGDC(\omega)|$ is shown in the Fig. A.5D.

- At each frequency, we obtain the maximum phase coherence across channels (cross-regional effect). That is, $\psi_{r,\omega}(t) - \theta_{r',\omega}(t) = \text{const} \implies |PLV| = 1 \ \forall\ \omega$, where the phase time series $\psi_{r,\omega}(t)$ and $\theta_{r',\omega}(t)$ were extracted from different channels ($(r, r') \in \{1, 2, 3\} : r \neq r'$) and evaluated at the same frequency ω. In other words, $\psi_{r,\omega}(t)$ and $\theta_{r',\omega}(t)$ are the phase time series associated with two tones homologous in frequency and pertaining to different channels. The resulting $|PLV|$ is shown in the Fig. A.5D.

It is essential to note that, the $SGDC(\omega)$ measure is highly sensitive to the cross-regional synchronization of the salient events, whereas the PLV measure is completely blind to this effect (compare Figs. A.5B and A.5D).

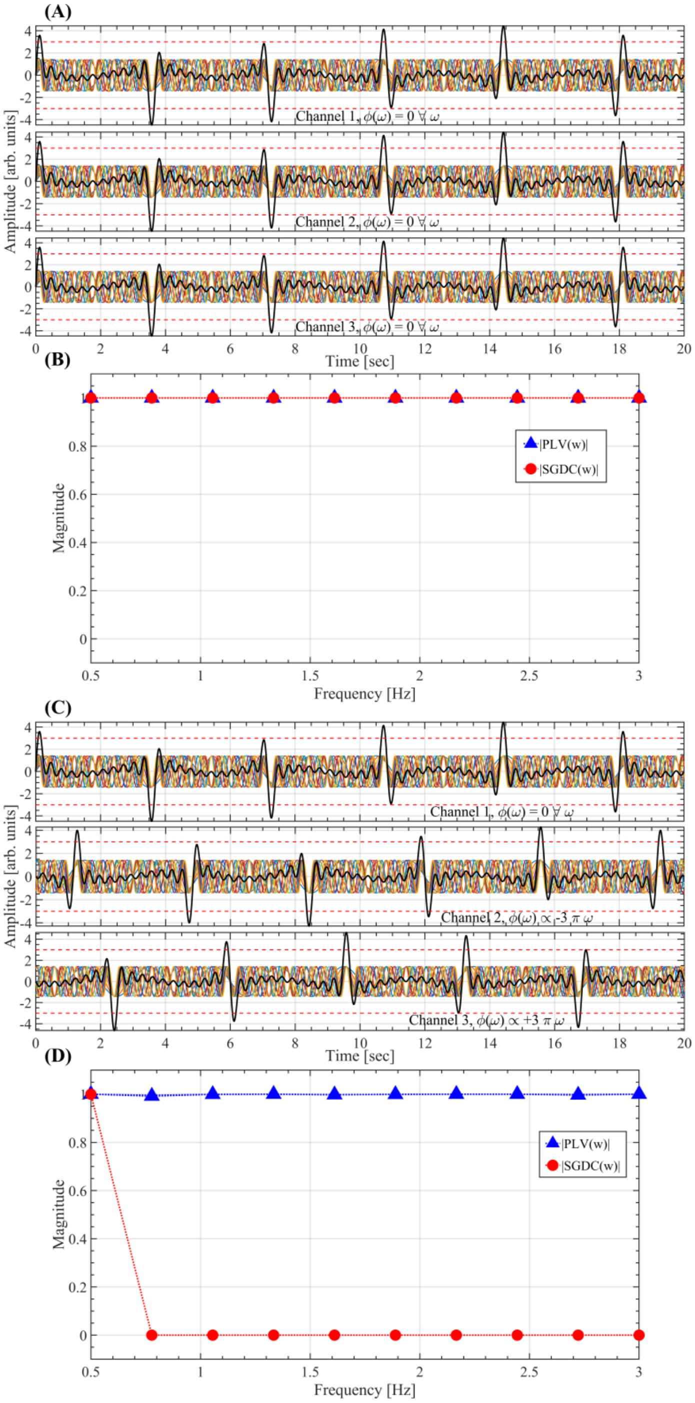

Figure A.5: $SGDC(\omega)$ computed using the Eq. A.19 on a multi-channel configuration. (A) Three time series $x_r(t)$ (black solid lines) synthesized using the Eq. A.11. In each channel, the signal $x_r(t)$ was synthesized by the linear superposition of 10 sinusoidal tones (colored solid lines) with unitary amplitude and uniformly spaced frequencies ($f_s \; \Delta\omega/(2\pi) = 0.278$ Hz) within the range $f_s \; \omega(k)/(2\pi) = 0.5 + k \; f_s \; \Delta\omega/(2\pi) \in [0.5 - 3]$ Hz. In each channel, the phase of all the oscillatory components was set to zero ($\phi_r(\omega) = 0 \; \forall \; \omega$). (B) $SGDC(\omega)$ and PLV measures computed using the Eqs. A.19 and A.27, respectively, for the multi-channel configuration shown in panel A. (C) Same as in A, but in this case the phase of the tones were set as $\phi_1(\omega) = 0$, $\phi_2(\omega) \propto -3\pi\omega$ and $\phi_3(\omega) \propto +3\pi\omega$ for the channel 1, 2 and 3, respectively. (D) Same as in B for the multi-channel configuration shown in panel C.

Fig. A.6A shows three time series constituted by the same 10 tones used in Figs. A.5A and A.5C with the difference that in this case the phase of the tones were set as follows,

$$\begin{aligned} \phi_1(\omega) &= 0 \ \forall \ \omega \ \text{(LF+HF)} \\ \phi_2(\omega) &\propto \begin{cases} 0, & \forall \ 0.5\text{Hz} \leq f_s \ \omega/(2\pi) \leq 1.5\text{Hz (LF)} \\ -3\pi\omega, & \forall \ 1.5\text{Hz} \leq f_s \ \omega/(2\pi) \leq 3\text{Hz (HF)} \end{cases} \\ \phi_3(\omega) &\propto \begin{cases} 0, & \forall \ 0.5\text{Hz} \leq f_s \ \omega/(2\pi) \leq 1.5\text{Hz (LF)} \\ +3\pi\omega, & \forall \ 0.5\text{Hz} \leq f_s \ \omega/(2\pi) \leq 1.5\text{Hz (HF)} \end{cases} \end{aligned} \tag{A.28}$$

This phase configuration produce LF transient fluctuations co-occurring across the channels, while the resulting HF transient fluctuations are not synchronized across the channels (see Fig. A.6A). Importantly, the $SGDC(\omega)$ effectively discriminate the cross-regional synchronization of the transient fluctuations across the frequency values, whereas the PLV measure is again completely blind to this effect (see Fig. A.6B). Fig. A.6C shows three time series constituted by the same 10 tones used in Fig. A.6A (see Eq. A.11) with the difference that in this case the phase of the tones were set as follows,

$$\begin{aligned} \phi_1(\omega) &= 0 \ \forall \ \omega \ \text{(LF+HF)} \\ \phi_2(\omega) &\propto \begin{cases} -3\pi\omega, & \forall \ 0.5\text{Hz} \leq f_s \ \omega/(2\pi) \leq 1.5\text{Hz (LF)} \\ 0, & \forall \ 1.5\text{Hz} \leq f_s \ \omega/(2\pi) \leq 3\text{Hz (HF)} \end{cases} \\ \phi_3(\omega) &\propto \begin{cases} +3\pi\omega, & \forall \ 0.5\text{Hz} \leq f_s \ \omega/(2\pi) \leq 1.5\text{Hz (LF)} \\ 0, & \forall \ 0.5\text{Hz} \leq f_s \ \omega/(2\pi) \leq 1.5\text{Hz (HF)} \end{cases} \end{aligned} \tag{A.29}$$

Similarly to the previous case, the $SGDC(\omega)$ effectively discriminate the cross-regional synchronization of the transient fluctuations across the frequency values, whereas the PLV measure is again completely blind to this effect (see Fig. A.6D). It is worth mentioning that $\Delta\phi_r(\omega)$ in the Eq. A.19 is the incremental phase between adjacent spectral components associated with the activity $x_r(t)$ of the brain region r. Thus, for N spectral components we obtain $N-1$ incremental phase values $\Delta\phi_r(\omega)$. As a convention, we add an extra value $\Delta\phi_r(\omega) = 0$ as the first element (i.e., lowest frequency) of the list of incremental phase values. Hence, for N spectral components the Eqs. A.18 and A.19 produce N values of SGDC. In particular, the first value (i.e., lowest frequency) of $SGDC(\omega)$, associated with the artificially added $\Delta\phi_r(\omega) = 0$, is always equal to 1 (this becomes evident in the Figs. A.5D and A.6D).

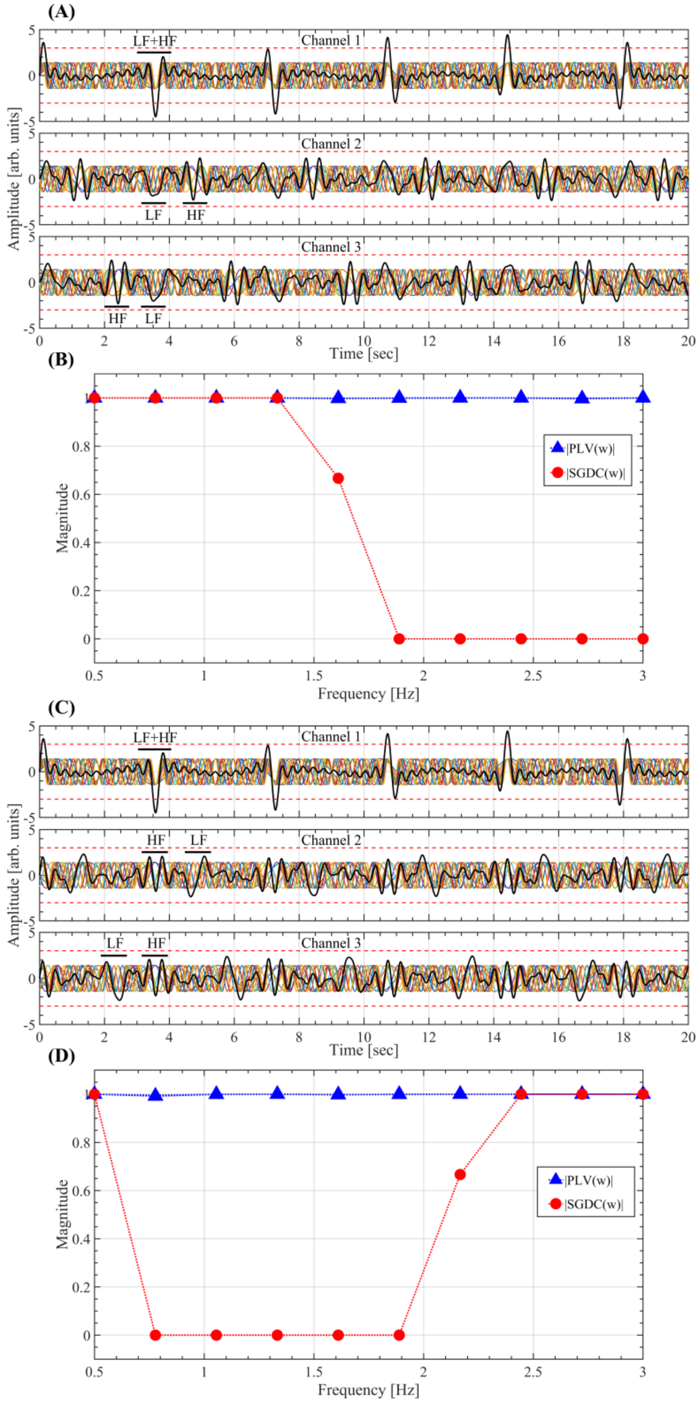

Figure A.6: $SGDC(\omega)$ computed using the Eq. A.19 on a multi-channel configuration. (A) Three time series $x_r(t)$ (black solid lines) synthesized using the Eq. A.11. In each channel, the signal $x_r(t)$ was synthesized by the linear superposition of 10 sinusoidal tones (colored solid lines) with unitary amplitude and uniformly spaced frequencies ($f_s\ \Delta\omega/(2\pi) = 0.278$ Hz) within the range $f_s\ \omega(k)/(2\pi) = 0.5 + k\ f_s\ \Delta\omega/(2\pi) \in [0.5 - 3]$ Hz. In each channel, the phases of the oscillatory components were configured as stated in the set of Eqs. A.28. (B) $SGDC(\omega)$ and PLV measures computed using the Eqs. A.19 and A.27, respectively, for the multi-channel configuration shown in panel A. (C) Same as in A, but in this case the phase of the tones were configured as stated in the set of Eqs. A.29. (D) Same as in B for the multi-channel configuration shown in panel C.

Supplementary Material A.4. Spectral group delay consistency in the surrogate data

Here we analytically show that, on the one hand, A-surrogates significantly reduce the spectral group delay consistency (SGDC) across both frequency components ($SGDC(r)$) and brain regions ($SGDC(\omega)$). On the other hand, B-surrogates significantly reduce the SGDC across frequency components ($SGDC(r)$), while preserving the SGDC across brain regions ($SGDC(\omega)$).

We start by recalling the definition of $SGDC(r)$ and $SGDC(\omega)$ for a multi-regional time series $x_r(t)$,

$$\begin{aligned}
\mathfrak{F}\left\{x_r(t)\right\} &= X_r(\omega) = A_r(\omega)e^{i\phi_r(\omega)} \\
\Delta\phi_r(\omega) &= \phi_r(\omega+\Delta\omega) - \phi_r(\omega) \\
SGDC(r) &= \frac{1}{N}\sum_{\omega} e^{-i\Delta\phi_r(\omega)} : \Delta\omega = \text{const across } r \qquad \text{(A.30)} \\
SGDC(\omega) &= \frac{1}{N}\sum_{r} e^{-i\Delta\phi_r(\omega)} : \Delta\omega = \text{const across } \omega \qquad \text{(A.31)}
\end{aligned}$$

where $A_r(\omega)$ and $\phi_r(\omega)$ are the amplitude and phase Fourier spectra, respectively. In Eqs. A.30 and A.31, N is the number of either frequency values or brain regions, respectively, and $\Delta\phi_r(\omega)$ is the incremental phase computed across the spectral components of the DFT spectrum $X_r(\omega)$ associated with the signals $x_r(t)$. In the case of the surrogate multi-regional time series $x_r^s(t)$, obtained by phase randomization of the original time series in the frequency-domain, we have,

$$\begin{aligned}
\mathfrak{F}\left\{x_r^s(t)\right\} &= X_r^s(\omega) = A_r(\omega)e^{i(\phi_r(\omega)+\theta_r(\omega))} \\
SGDC^s(r) &= \frac{1}{N}\sum_{\omega} e^{-i(\Delta\phi_r(\omega)+\Delta\theta_r(\omega))} : \Delta\omega = \text{const across } r \ \text{(A.32)} \\
SGDC^s(\omega) &= \frac{1}{N}\sum_{r} e^{-i(\Delta\phi_r(\omega)+\Delta\theta_r(\omega))} : \Delta\omega = \text{const across } \omega \text{(A.33)}
\end{aligned}$$

In the Eqs. A.32 and A.33, $\Delta\theta_r(\omega)$ is the incremental phase associated with the random phase-shift $\theta_r(\omega)$ extracted from the surrogate DFT spectrum $X_r^s(\omega)$ of each brain region r. Let us consider two extreme cases derived from the Eqs. A.30 and A.32 with $\Delta\theta_r(\omega)$ varying randomly across ω,

1 For $\Delta\phi_r(\omega) \approx const \implies |SGDC(r)| \approx 1 > |SGDC^s(r)| \approx 0$.

2 For $\Delta\phi_r(\omega)$ varying randomly across $\omega \implies |SGDC(r)| \approx |SGDC^s(r)| \approx 0$.

From these two extreme cases we infer that, for $\theta_r(\omega)$ varying randomly across ω, $|SGDC(r)|$ is the upper bound of $|SGDC^s(r)|$. As a consequence, for the A- and B-surrogates in general we obtain $|SGDC^s(r)| < |SGDC(r)|$. Similarly, in the case of A-surrogates computed with $\theta_r(\omega)$ varying randomly across the brain regions r, Eqs. A.31 and A.33 in general produce $|SGDC^s(\omega)| < |SGDC(\omega)|$. In the particular case of the B-surrogates, at each frequency ω we add the

same phase-shift value $\theta_r(\omega)$ in all the brain regions r, producing $\Delta\theta_r(\omega) = \Delta\theta(\omega)\ \forall\ 1 \leq r \leq N$. As a consequence, by taking the modulus in both sides of the Eq. A.33 we obtain the equivalence between the true data and the B-surrogate in terms of $|SGDC(\omega)|$,

$$\begin{aligned} |SGDC^s(\omega)| &= \left| e^{-i\Delta\theta(\omega)} \frac{1}{N} \sum_r e^{-i\Delta\phi_r(\omega)} \right| \\ &= \left| e^{-i\Delta\theta(\omega)} SGDC(\omega) \right| \\ &= |SGDC(\omega)| \end{aligned}$$

We confirmed this analytical results by computing the $SGDC(r)$ and $SGDC(\omega)$ measures on the whole time series of our empirical MEG dataset and the corresponding A- and B-surrogates (see Section 2.8 in Methods). Fig. A.7A shows that the magnitude of the $SGDC(r)$ measure is not preserved in both the A- and B-surrogates. Besides, Fig. A.7B shows that the magnitude of the $SGDC(\omega)$ measure is preserved in the B-surrogates, and not in the case of A-surrogates. Importantly, the reduction of the regional SGDC, as quantified by the $SGDC(r)$ measure, offers an analytical rationale supporting the evidence showing that B-surrogates failed to reproduce the SEs observed in our MEG dataset (see Section 3.2) despite preserving both the regional PSDs and the cross-spectra (see Supplementary Material A.1). It is important to note that this equivalence between the true MEG data and the B-surrogates in terms of $|SGDC(\omega)|$ holds only when the $SGDC(\omega)$ measure is computed on the whole time series (i.e., non-time-resolved approach). On the other hand, if the $SGDC(r)$ and $SGDC(\omega)$ measures are computed in a time-resolved manner on each salient event (see Fig. A.7C,D), the equivalence between the true MEG data and the B-surrogates in terms of $|SGDC(\omega)|$ does not longer hold. This is mainly due to the fact that true SEs and B-surrogate SEs are different in duration and size and, more crucially, they do not necessarily involve the same brain regions.

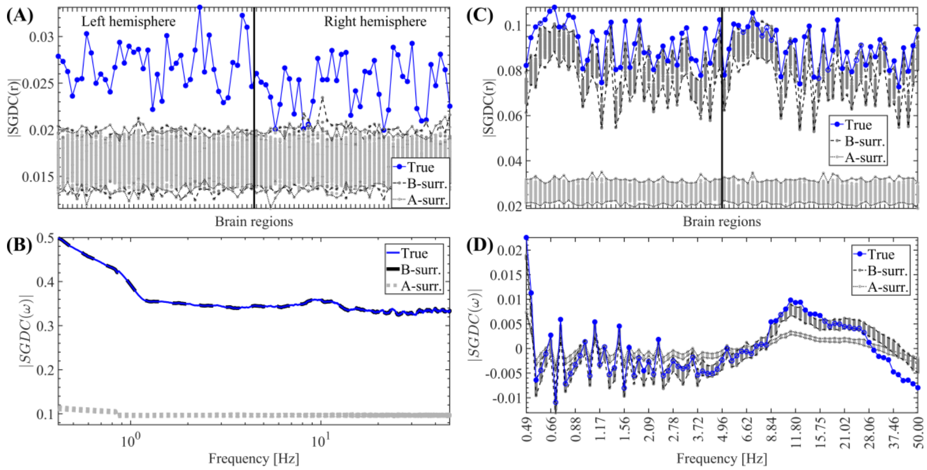

Figure A.7: Spatial profiles associated with the SGDC measures. (A) $SGDC(r)$ measure computed on the whole time series of each brain region (i.e., non-time-resolved approach). (B) $SGDC(\omega)$ measure computed on the whole time series of each brain region (i.e., non-time-resolved approach). Note that the pattern corresponding to the 100 B-surrogates (thick dashed black line) overlap with the spatial profile associated with the true MEG data (thin blue line). (C) $SGDC(r)$ measure computed on each detected SE by considering the brain regions and time interval associated with each particular event (i.e., time-resolved approach). (D) $SGDC(\omega)$ measure computed on each detected SE by considering the brain regions and time interval associated with each particular event (i.e., time-resolved approach). The labels and ordering of the brain regions are the same as those shown in Fig. C.2. Symbols and abbreviations: SE, Salient Event.

Supplementary Material B. Supplementary numerical modeling results

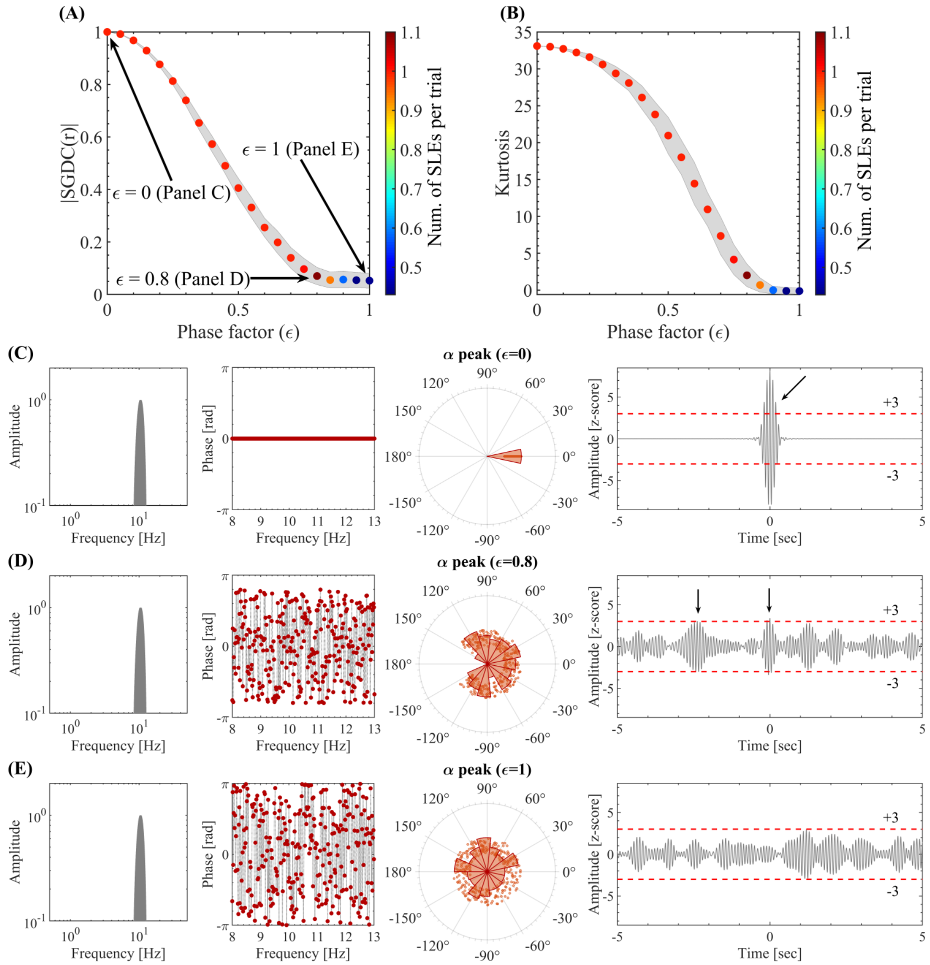

Figure B.1: Spectral group delay consistency underlies the emergence of local above-threshold fluctuations from NOs. (A) Spectral group delay consistency, as quantified by the $SGDC(r)$ measure, as a function of the phase factor values (ϵ). The colored markers indicate the mean $|SGDC(r)|$ value across 100 synthetic time series of 10 sec in duration (trials). The shaded error bars in gray correspond to the standard deviation around the mean value. The pseudocolor scale represents the mean number of SLEs per trial. The $SGDC(r)$ measure was obtained by computing the Eq. 1 on the synthetic phase values assigned to the spectral components in the alpha band. (B) Same as in A for the Kurtosis of the time series amplitude values, obtained by computing the Eq. A.25 on the signals in time-domain. (C) Amplitude spectrum (left), phase spectrum and distribution (middle), and resulting time series (right) corresponding to the signal model for a phase factor $\epsilon = 0$. For the amplitude spectrum we used a Hann window with a null-to-null bandwidth = 8-13 Hz, frequency resolution df = 1/60sec $\approx$ 0.017 Hz. The phase values of the spectral components were constrained within the range $[-\epsilon\pi, \epsilon\pi]$ and having a random dependence with the frequency. The black arrows in the right-most panel highlight the above-threshold fluctuations disclosed by the signal. (D) Same as in C for a phase factor $\epsilon = 0.8$. (E) Same as in C for a phase factor $\epsilon = 1$. Symbols and abbreviations: SLEs, Salient Local Events; SGDC, Spectral Group Delay Consistency.

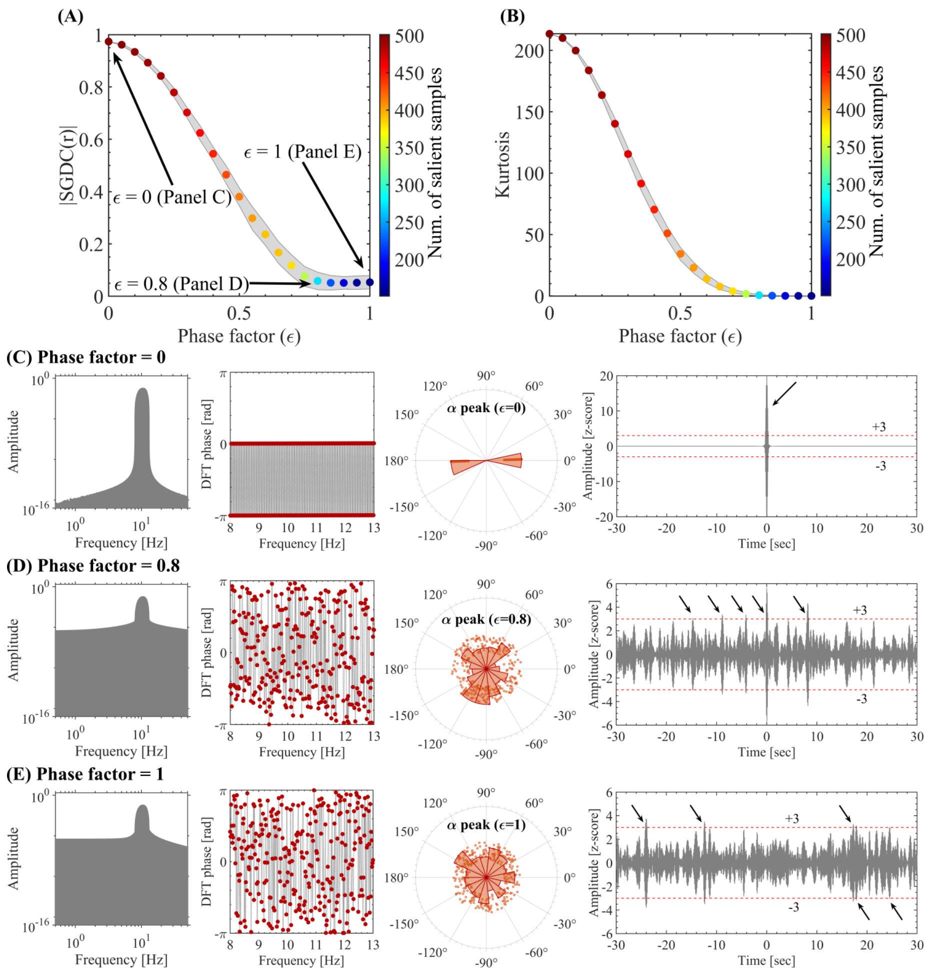

Figure B.2: Spectral group delay consistency underlies the emergence of local above-threshold fluctuations from NOs. (A) Spectral group delay consistency, as quantified by the $SGDC(r)$ measure, as a function of the phase factor values (ϵ). The colored markers indicate the mean $|SGDC(r)|$ value across 100 synthetic time series of 60 sec in duration (trials). The shaded error bars in gray correspond to the standard deviation around the mean value. The pseudocolor scale represents the mean number of SLEs per trial. The $SGDC(r)$ (Eq. 1) was computed using the alpha band phases obtained from the DFT applied to the time series resulting from the signal model (e.g., see the 60 sec in duration signals shown in panels C, D and E). This procedure inherently introduces spectral leakage due to the time-domain tapering (rectangular window), which affects the alpha band phase values involved in the computation of the $SGDC(r)$ measure and is visible in the corresponding power spectra shown in panels C, D and E. (B) Same as in A for the Kurtosis of the time series amplitude values, obtained by computing the Eq. A.25 on the signals in time-domain. (C) Amplitude spectrum (left), phase spectrum and distribution (middle), and resulting time series (right) corresponding to the signal model for a phase factor $\epsilon = 0$. The phase values of the spectral components were constrained within the range $[-\epsilon\pi, \epsilon\pi]$ and having a random dependence with the frequency. The black arrows in the right-most panel highlight the above-threshold fluctuations disclosed by the signal. (D) Same as in C for a phase factor $\epsilon = 0.8$. (E) Same as in C for a phase factor $\epsilon = 1$. Symbols and abbreviations: SLEs, Salient Local Events; SGDC, Spectral Group Delay Consistency.

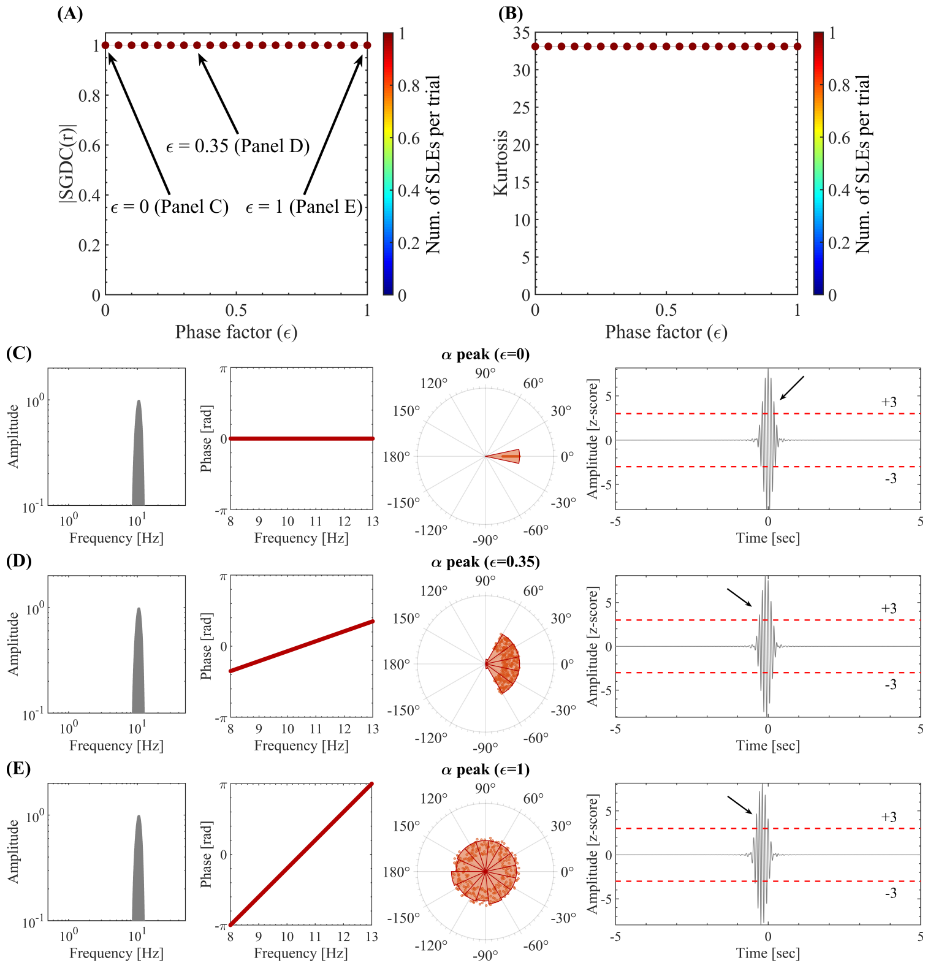

Figure B.3: Spectral group delay consistency underlies the emergence of local above-threshold fluctuations from NOs. (A) Spectral group delay consistency, as quantified by the $SGDC(r)$ measure, as a function of the phase factor values (ϵ). The colored markers indicate the mean $|SGDC(r)|$ value across 100 synthetic time series of 10 sec in duration (trials). The shaded error bars in gray correspond to the standard deviation around the mean value. The pseudocolor scale represents the mean number of SLEs per trial. The $SGDC(r)$ measure was obtained by computing the Eq. 1 on the synthetic phase values assigned to the spectral components in the alpha band. (B) Same as in A for the Kurtosis of the time series amplitude values, obtained by computing the Eq. A.25 on the signals in time-domain. (C) Amplitude spectrum (left), phase spectrum and distribution (middle), and resulting time series (right) corresponding to the signal model for a phase factor $\epsilon = 0$. For the amplitude spectrum we used a Hann window with a null-to-null bandwidth = 8-13 Hz, frequency resolution df = 1/60sec $\approx$ 0.017 Hz. The phase values of the spectral components were constrained within the range $[-\epsilon\pi, \epsilon\pi]$ and having a linear dependence with the frequency. The black arrows in the right-most panel highlight the above-threshold fluctuations disclosed by the signal. (D) Same as in C for a phase factor $\epsilon = 0.8$. (E) Same as in C for a phase factor $\epsilon = 1$. Symbols and abbreviations: SLEs, Salient Local Events; SGDC, Spectral Group Delay Consistency.

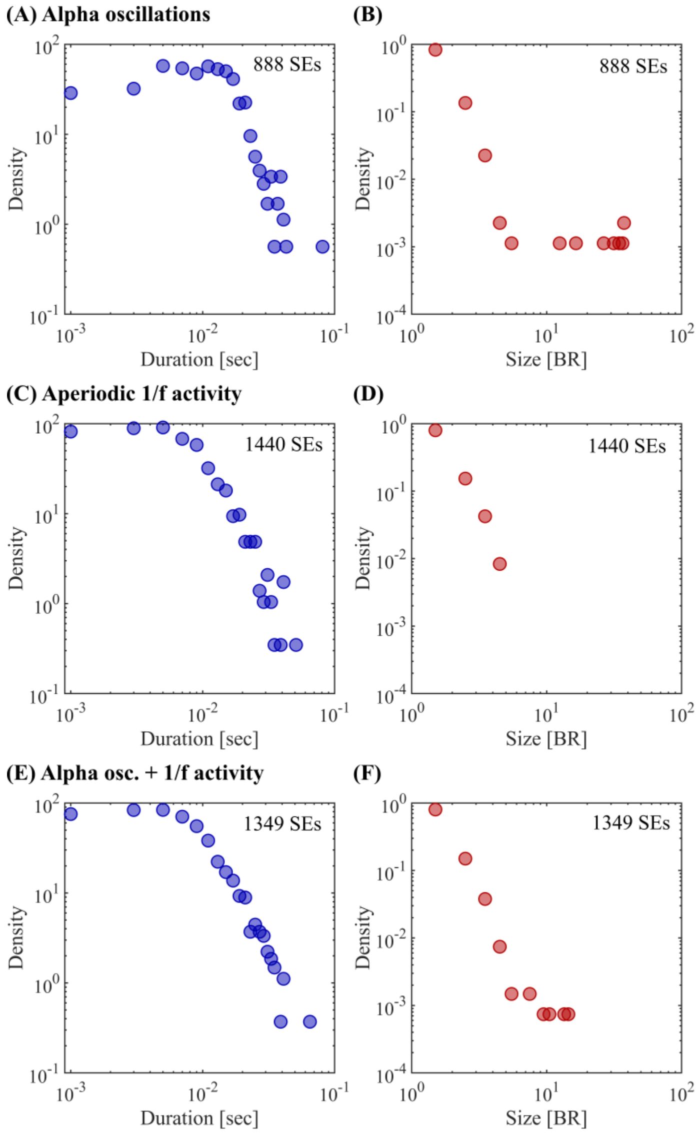

Figure B.4: Distributions of size and duration corresponding to the SEs detected in the large-scale signal model. (A-B) Large-scale model for SEs including only alpha oscillations (random phase values in the alpha band constrained to the range $[-\epsilon\pi, \epsilon\pi]$ with $\epsilon \in [0.75, 1]$). Panels A and B show the distribution of SEs duration and size, respectively, computed on all the SEs detected in a simulated time series of 1-minute duration. See Figs. 6A,B. (C-D) Same as in A-B for the large-scale model including only broadband $1/f$ activity, and no oscillatory activity in the alpha band nor phase consistency values were present ($\epsilon = 1$). See Figs. 6C,D. (E-F) Same as in A-B for the large-scale model including both broadband $1/f$ activity with non-constrained random phases ($\epsilon = 1$) and alpha oscillations with random phases constrained proportionally to the observed alpha power in the range ($\epsilon \in [0.75, 1]$). See Figs. 6E,F. Symbols and abbreviations: SEs, Salient Events.

Supplementary Material C. Supplementary empirical results including the deep sources

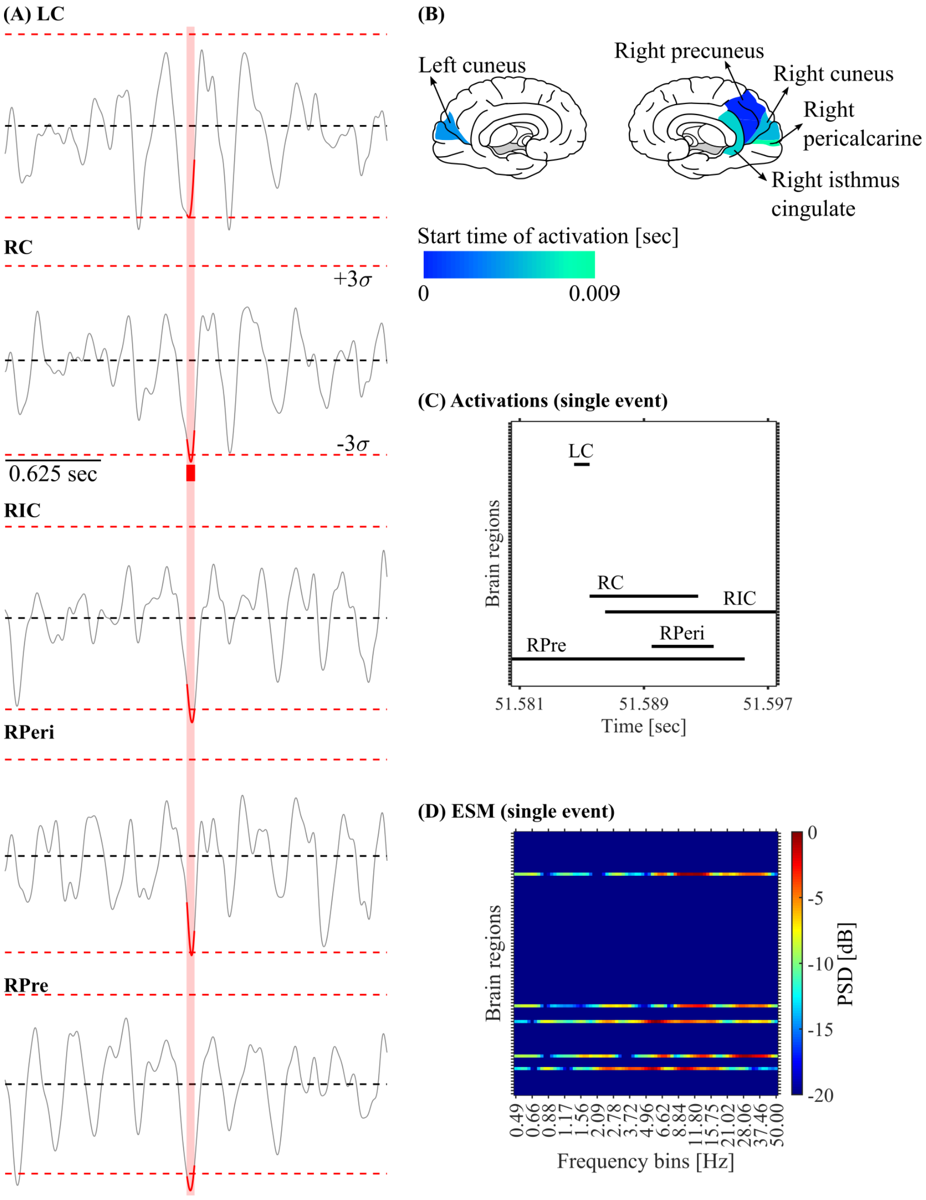

Figure C.1: Salient Network Event (SNE). (A) Z-scored time series disclosing the above-threshold fluctuations associated with a SNE observed in the source-reconstructed MEG data. The time interval in which at least one brain region is active (i.e., duration of the SNE) is highlighted in red. (B) Brain plots showing the activation start time of the 5 brain regions recruited by the SNE shown in panel A. (C) Activation matrix of the SNE shown in panel A. The black segments correspond to the time intervals in which each brain region was active (i.e., absolute amplitude $> 3\sigma$). (D) ESM corresponding to the SNE shown in panel A. Symbols and abbreviations: ESM, Event Spectral Matrix; MEG, Magnetoencephalography; RPre, Right Precuneus; RC, Right Cuneus; RPeri, Right Pericalcarine; RIC, Right Isthmus Cingulate; LC, Left Cuneus.

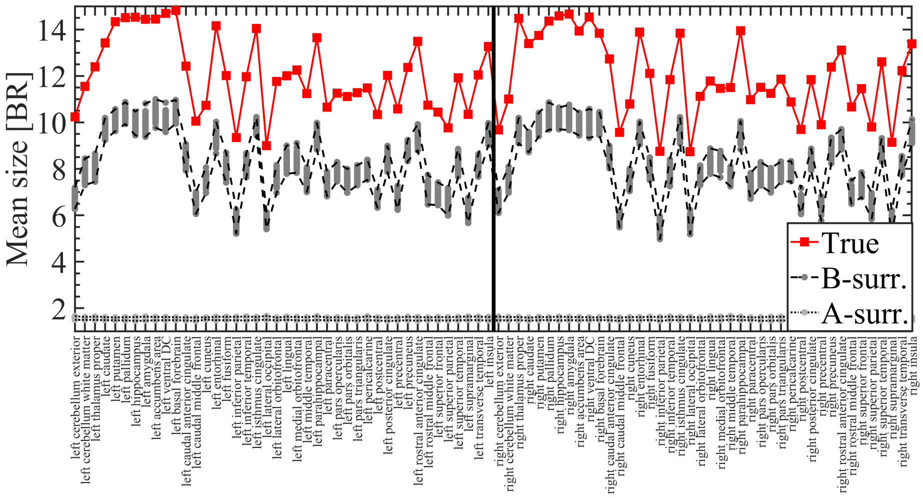

Figure C.2: Labels and ordering of the brain regions used to compute all the spatial profiles shown in this work. Spatial profile showing the mean size of SEs propagating through each brain region (mean value across the 47 participants, see Section 2.4 in Methods). The mean event size is shown for the MEG data together with the 100 A- and B-surrogates (see Section 2.8 in Methods). Symbols and abbreviations: SEs, Salient Events; BR, Brain Regions.

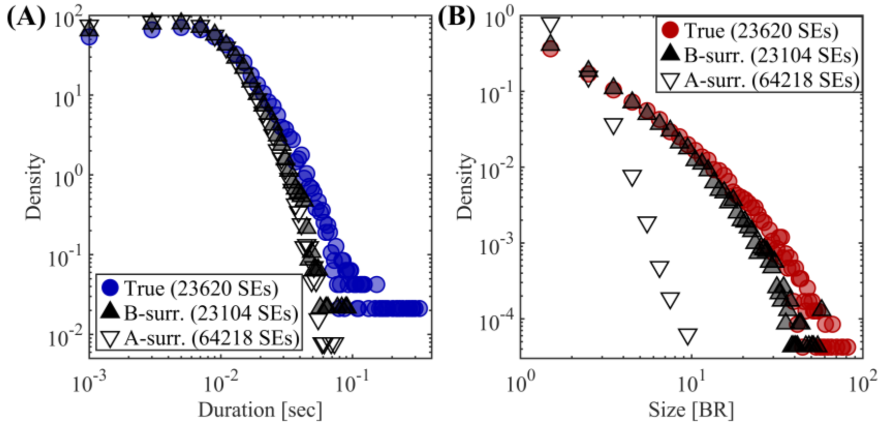

Figure C.3: Statistical characterization of SEs. (A) Distribution of the duration of SEs observed in the true source-reconstructed MEG data (filled blue circles), the A-surrogate (empty down-pointing triangles) and the B-surrogate (filled up-pointing triangles) corresponding to a time binning of 1 time sample per time bin (time binning = 1 ms). In the three cases the SEs were computed on the 47 participants. (B) Same as in A for the size of SEs. To test the significance of the difference of the distribution means between the true MEG data and the surrogates (A and B), we computed a non-parametric permutation test (random sampling without replacement, 1×10^4 permutations). The distributions of the duration and size of SEs observed in the true source-reconstructed MEG data, disclosed statistically significant differences with respect to both A- and B- surrogates ($P < 0.001$). Symbols and abbreviations: SEs, Salient Events; BR, Brain Regions.

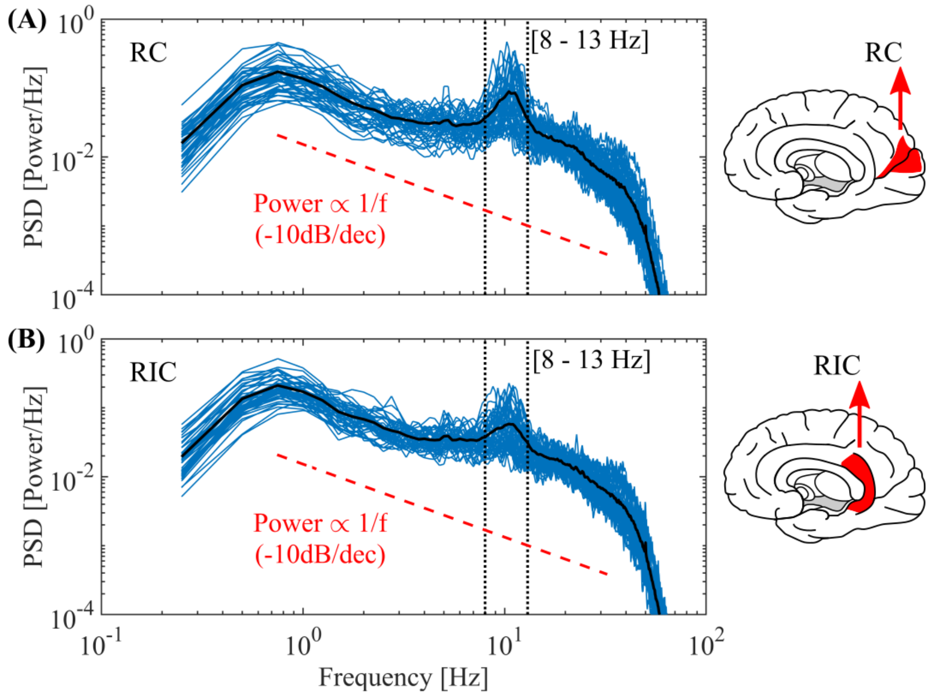

Figure C.4: Power Spectral Density (PSD). Power spectra computed on the Right Cuneus (RC, panel A) and the Right Isthmus Cingulate (RIC, panel B) activities of each patient (blue lines) and the resulting average (black line). The PSDs were computed on 1 min duration source-reconstructed MEG data of 47 subjects. Note that the PSDs of the RC (panel A) disclose a prominent bump in the alpha band (8-13 Hz) characteristic of the occipital brain regions, however, a less prominent bump in the alpha band is also observed in regions away from the occipital cortex (see the PSDs of RIC shown in panel B). Symbols and abbreviations: PSD, Power Spectral Density; RC, Right Cuneus; RIC, Right Isthmus Cingulate.

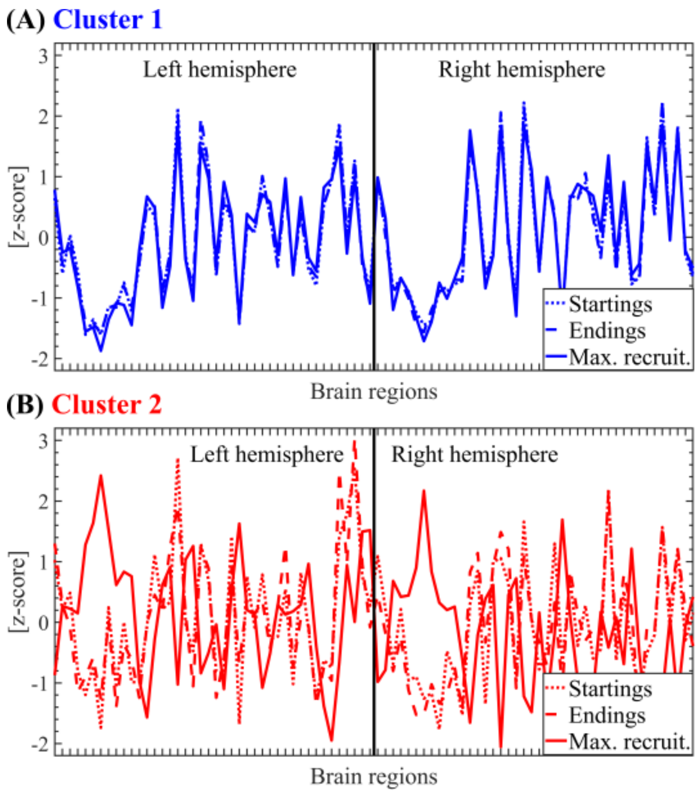

Figure C.5: Salient events propagation modes segregated by SE clusters. (A) Spatial profile for the cluster 1 SEs starting, maximum recruitment and ending modes (see Section 2.7 in Methods) computed on 41 participants. Linear correlations between topographies: Startings vs Endings, $r = 0.995$, $P < 0.001$. Max. recruit. vs Startings, $r = 0.978$, $P < 0.001$. Max. recruit. vs Endings, $r = 0.978$, $P < 0.001$. (B) Same as in A for the cluster 2 SEs starting, maximum recruitment and ending modes. Linear correlations between topographies: Startings vs Endings, $r = 0.895$, $P < 0.001$. Max. recruit. vs Startings, $r = -0.298$, $P < 0.01$. Max. recruit. vs Endings, $r = -0.280$, $P < 0.01$. The SEs obtained from 41 subjects were clustered using the Louvain algorithm (resolution parameter $\gamma = 1$, see Section 2.9 in Methods). The reported P values for the statistical significance of the Pearson's correlation were assessed using Student's t distributions of the two-tailed hypothesis test under the null hypothesis that the correlation is zero. Symbols and abbreviations: SEs, Salient Events.

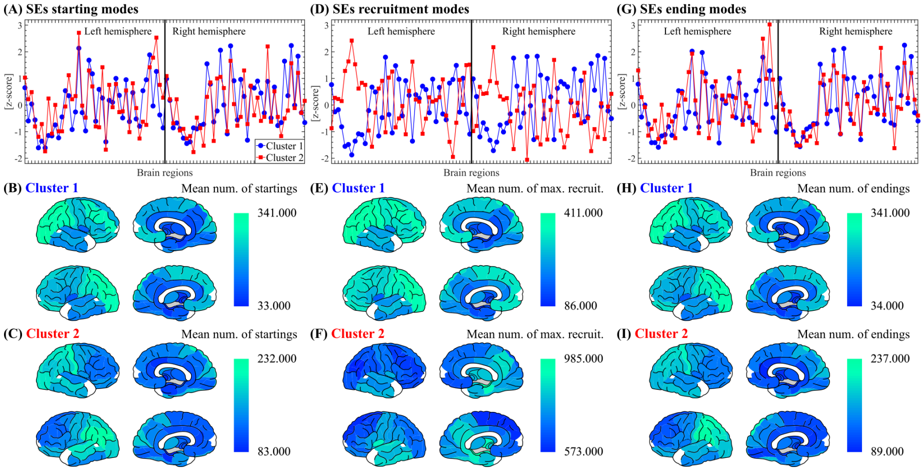

Figure C.6: Salient events propagation modes. (A) Spatial profile for the SEs starting modes (see Section 2.7 in Methods) corresponding to the two SE clusters computed on 41 participants. The SEs obtained from 41 subjects were clustered using the Louvain algorithm (resolution parameter $\gamma = 1$, see Section 2.9 in Methods). The Pearson's correlation between the spatial profiles of cluster 1 and cluster 2 SEs is $r = 0.708$, $P < 0.001$. (B) Brain topographies for the starting modes of cluster 1 SEs as shown in panel A. (C) Brain topographies for the starting modes of cluster 2 SEs as shown in panel A. (D-F) Same as A-C for SEs maximum recruitment modes (see Section 2.7 in Methods). In panel D, the Pearson's correlation between the spatial profiles of cluster 1 and cluster 2 SEs is $r = -0.841$, $P < 0.001$. (G-I) Same as A-C for SEs ending modes (see Section 2.7 in Methods). In panel G, the Pearson's correlation between the spatial profiles of cluster 1 and cluster 2 SEs is $r = 0.718$, $P < 0.001$. The reported P values for the statistical significance of the Pearson's correlation were assessed using Student's t distributions of the two-tailed hypothesis test under the null hypothesis that the correlation is zero. Symbols and abbreviations: SEs, Salient Events.

Supplementary Material C.1. Amplitude threshold analysis

The validity and robustness of using a single amplitude threshold ($|z| = 3$) consistently across all 47 participants was investigated as follows. In each participant, the 1-minute source-reconstructed MEG time series of each brain region were first individually z-scored and then concatenated across all brain regions. Subsequently, we computed the histogram and estimated the empirical Probability Density Function (empirical PDF) corresponding to the amplitude values of the concatenated time series (see blue curves in Figs. C.7A and C.7B). Next, we compute the Gaussian distribution that best fit the empirical PDF within each of the 100 fitting intervals of amplitude values spanning the range $[Q_1(z) - 5 * IQR(z), Q_3(z) + 5 * IQR(z)]$, where Q_1, Q_3, and IQR denote the first quartile, the third quartile and the interquartile range, respectively. This procedure yielded 100 Gaussian PDFs (see grey lines in Fig. C.7A). After that, we computed the RMS error between the empirical PDF and each of the 100 Gaussian PDFs. Where the RMS error was computed using a weighted difference to assign less importance to the difference in the tails of the distributions. As a result of this procedure, we obtained 100 RMS values (see Fig. C.7C). Finally, the optimal threshold for each participant was computed as half the

fitting interval of amplitude values producing the minimum RMS error (see Fig. C.7B and the red arrow in Fig. C.7C). Note that the minimum RMS error is associated with the amplitude value (optimal threshold) beyond which the empirical PDF significantly departs from the (best fitted) Gaussian distribution. This procedure was applied separately to all the 47 participants included in the study (see Fig. C.8). The mean and standard deviation of the amplitude thresholds corresponding to the true MEG data shown in Fig. C.8 are 3.08 ± 0.23. Importantly, the amplitude threshold used in this study ($|z| = 3$) lies approximately at the center of this range. The procedure described above for identifying the optimal amplitude threshold, based on minimizing the RMS error between the empirical PDF and the Gaussian PDFs, was also applied to one A-surrogate and one B-surrogate generated for each participant (see Fig. C.8). The mean and standard deviation of the $|z|$ thresholds across participants were 5 ± 0.23 for the A-surrogates and 4.7 ± 0.58 for the B-surrogates, respectively. Of note, the $|z|$ thresholds for the A- and B-surrogates were substantially higher than those for the true MEG data. This result is consistent with the fact that the phase randomization applied in the construction of A- and B-surrogates produces approximately Gaussian signals (Prichard and Theiler, 1994).

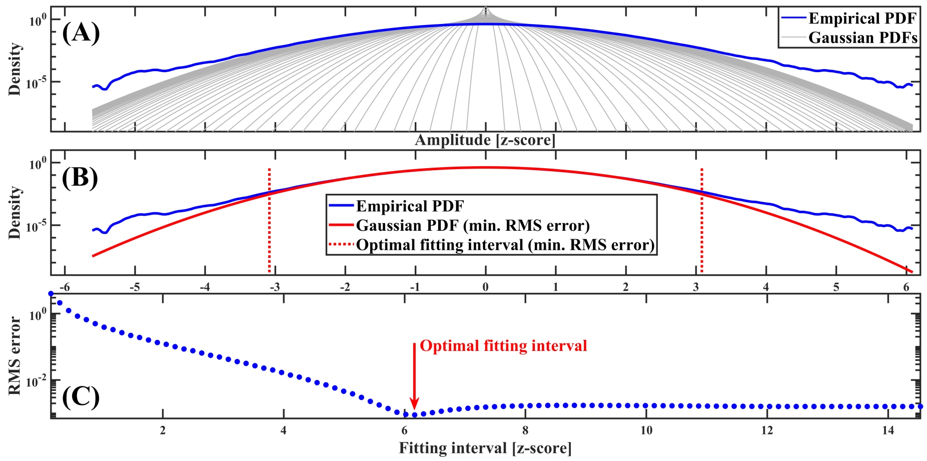

Figure C.7: Procedure to find the optimal $|z|$ threshold for Participant 47. (A) Empirical and the 100 Gaussian PDFs corresponding to the 100 fitting $|z|$ intervals. (B) Empirical PDF together with the Gaussian PDF producing the minimum RMS error. (C) RMS error between the empirical PDF and each of the 100 Gaussian PDFs. Symbols and abbreviations: SNE, Salient Network Event; PDF, Probability Density Function; RMS, Root Mean Square.

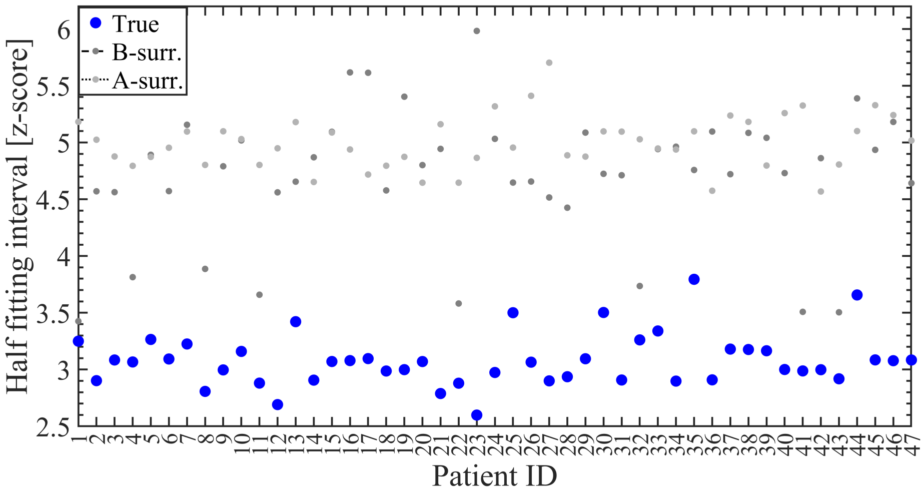

Figure C.8: Optimal $|z|$ thresholds for the 47 participants corresponding to the true MEG data including the deep sources, and one A-surrogate and one B-surrogate generated for each participant. The mean and standard deviation of the amplitude thresholds corresponding to the true MEG data (blue circles) are 3.08 ± 0.23. Symbols and abbreviations: MEG, Magnetoencephalography.

One of the main limitations of this study is related to the uncertain capability of our dataset to accurately identify deep brain sources along the cortical surface, mainly due to the ill-posed nature of the source-reconstructed MEG data. In order to address this issue, we re-computed the thresholding analysis presented above, but this time excluding the deep sources (see brain topographies in Figs. D.1F and D.3). The results are shown in Fig. C.9. It was found that the mean and standard deviation of the amplitude thresholds corresponding to the true MEG data excluding the deep sources are 3.08 ± 0.24. Importantly, the amplitude threshold used in this study ($|z| = 3$) lies approximately at the center of this range. Besides, the mean and standard deviation of the $|z|$ thresholds across participants were 5 ± 0.23 for the A-surrogates and 4.68 ± 0.59 for the B-surrogates, respectively. As a result, by comparing Figs. C.8 and C.9 we can conclude that the optimal $|z|$ thresholds remain essentially unaltered across the 47 participants when the deep sources are excluded from the thresholding analysis in our MEG dataset.

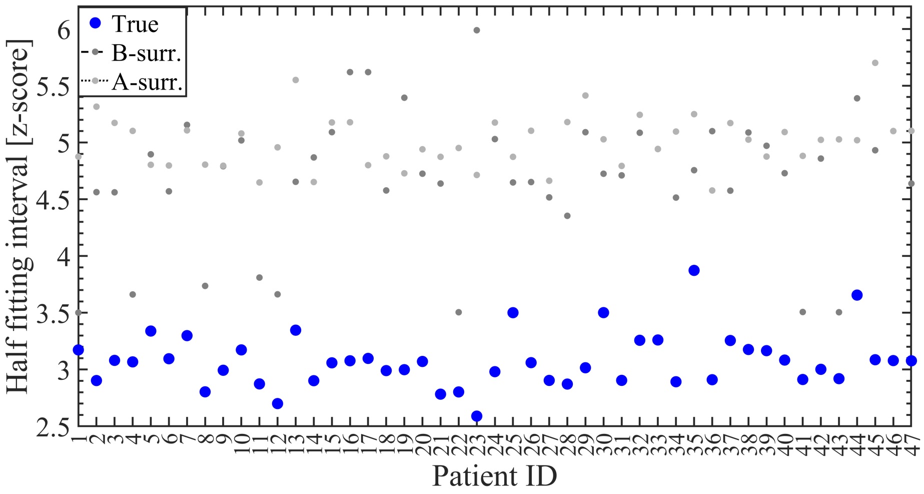

Figure C.9: Optimal $|z|$ thresholds for the 47 participants corresponding to the true MEG data excluding the deep sources, and one A-surrogate and one B-surrogate generated for each participant. The mean and standard deviation of the amplitude thresholds corresponding to the true MEG data (blue circles) are 3.08 ± 0.24. Symbols and abbreviations: MEG, Magnetoencephalography.

Supplementary Material C.2. Spectral group delay consistency, transient cross-regional coherent NOs and BAA underlie SNEs

In Sections 3.4 and 3.5, we showed that the concurrent presence of BAA and NOs disclosing appropriate levels of SGDC, are two key ingredients sufficient to generate realistic above-threshold fluctuations in a single brain signal (i.e., SLEs). Importantly, we have analytically and computationally shown that only the consistency of the Fourier incremental phase values across frequencies (SGDC) provides a quantitative measure of the level of salience of the above-threshold fluctuations exhibited by the signal in the time-domain, and this relationship holds true regardless of the spectral leakage introduced by tapering in the time-domain (see Fig. B.2). In this section, we present empirical evidence supporting the theoretical findings described in Sections 3.4 and 3.5. Fig. C.10A shows the topography of the mean number of salient (above-threshold) samples assessed in each brain region. The Panels B and C of Fig. C.10 show, respectively, the time series and distributions of the amplitude values corresponding to the brain regions disclosing the maximum (Left supramarginal) and minimum (Left superior frontal) number of salient samples. Importantly, the scatter plots in Panels D and E of Fig. C.10 show a significant correlation between the topographies of the salient samples (Panel A) and, respectively, the $SGDC(r)$ magnitude and kurtosis. This empirical evidence, together with the results shown in Figs. B.1, B.2 and B.3, further supports the interpretation of the $SGDC(r)$ as a measure capturing the signal-level mechanism underlying the emergence of local above-threshold fluctuations.

Next, we present the rationale and results pointing out that SGDC is a key conceptualization also in connection with the emergence of realistic SNEs as collective phenomena involving multiple brain regions. Although the $SGDC(r)$ measure assesses the emergence of local above-threshold fluctuations from the Fourier oscillatory constituents of the activity in a single brain region (i.e., SLEs), it does not account for cross-regional effects associated with SNEs. To quantitatively study the cross-regional effects of SGDC on our data we introduce the $SGDC(\omega)$ measure. The magnitude of $SGDC(\omega)$ is bounded in the range $[0, 1]$ and quantifies how much the group delay at a given frequency ω varies across brain regions (Eq. 2). By using synthetic time series, in Supplementary Material A.3 we show that the $SGDC(\omega)$ measure assesses the contribution of each frequency component in the co-activation (synchronization in time) of above-threshold fluctuations across brain regions (see Figs. A.5 and A.6). Of note, Figs. A.5 and A.6 show that the $SGDC(\omega)$ measure effectively resolves the cross-regional synchronization of SEs across frequency bands, whereas phase coherence measures (e.g., PLV: Phase Locking Value) are completely blind to this effect. Then, we used the $SGDC(\omega)$ measure to analyze the two SE clusters observed in our empirical MEG data. Figs. C.11A,B show the average ESMs of the two SE clusters identified by the Louvain algorithm (see Methods) computed on 10 subjects. As shown in Fig. C.11C, only cluster 2 SEs are associated $|SGDC(r)|$ values higher than those disclosed by the C-surrogate SEs. Importantly, Fig. C.11D shows the increase of transient cross-regional coherence around the alpha band, as quantified by the $SGDC(\omega)$ measure, associated with the SEs disclosing the alpha spectral signature in the average ESM (i.e., cluster 2 SEs). These results are further evidence pointing out that the cluster 2 SEs observed in our MEG data co-occur with (or are coupled to) alpha bursts propagating across brain regions. Notably, Fig. C.11E shows that the transient cross-regional coherence around the alpha band associated with the cluster 2 SEs is also captured by the large-scale model presented in Section 3.5.

Next, we used the $SGDC(\omega)$ measure to analyze the surrogate data computed via phase randomization. Our empirical results show that despite preserving both the power spectrum (PSD) in each brain region and the cross-correlations (i.e., functional connectivity) B-surrogates fail to account for the SEs observed in our MEG dataset. Besides, A-surrogates, which only preserve the regional PSD, perform worst than B-surrogates in reproducing realistic SEs (see Figs. 1 and C.3). The analytical derivations presented in Supplementary Material A.4 provide a unifying rationale for this evidence by pointing out that, on one hand, A-surrogates destroy both the burstiness of each brain region as assessed by the $SGDC(r)$ measure and the synchronization of above-threshold fluctuations across brain regions as assessed by the $SGDC(\omega)$ measure (see Figs. A.7A,B). On the other hand, B-surrogates significantly reduce the SGDC across frequency components ($SGDC(r)$, see Fig. A.7A), while preserving the SGDC across brain regions ($SGDC(\omega)$, see Fig. A.7B).

In summary, these results suggest that a) spectral group delay consistency in specific narrow frequency bands (as assessed by the $SGDC(r)$ measure), b) transient cross-regional coherent NOs (intra-frequency coherence across brain

regions assessed by the $SGDC(\omega)$ measure) and c) BAA, are all key ingredients for the emergence of realistic SEs.

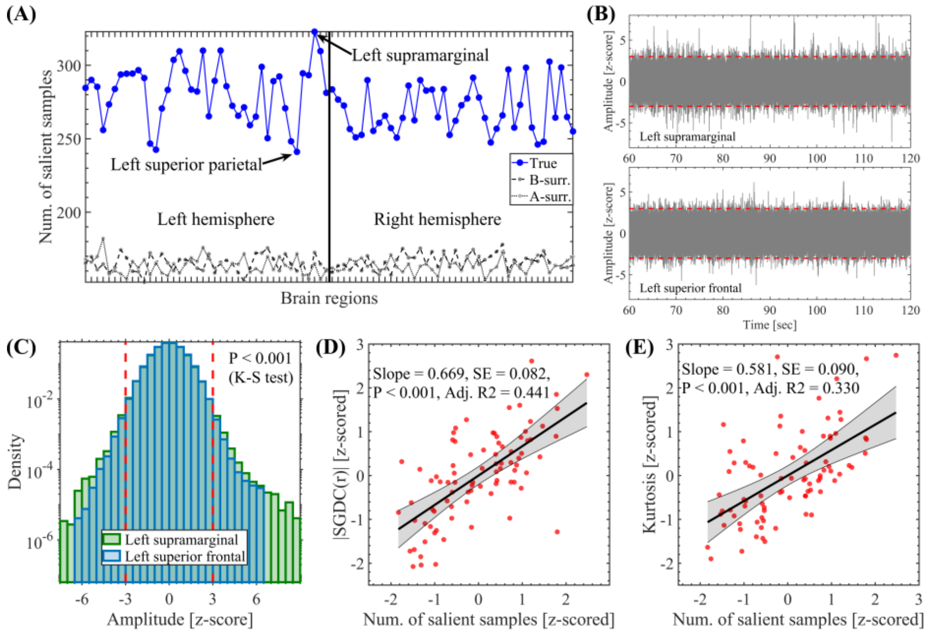

Figure C.10: Measures capturing the salient samples topographies. (A) Topography showing the number of salient samples computed on the whole time series (1 min in duration) of each brain region (mean value across the 47 participants). (B) Time series corresponding to the brain regions disclosing the maximum (Left supramarginal) and the minimum (Left superior frontal) number of salient samples. Each plot shows the time series superimposed across the 47 participants. (C) Distributions of the amplitude values for the Left supramarginal and Left superior frontal time series concatenated the 47 participants. Two-sample Kolmogorov-Smirnov test: $P < 0.001$. (D) Scatter plot showing the correlation between the topographies associated with the salient samples and the magnitude of the $SGDC(r)$ measure. Number of samples (red circles) = Number of brain regions = 84. (E) Same as in (D) for the kurtosis. Symbols and abbreviations: SGDC, Spectral Group Delay Consistency, K-S, Kolmogorov-Smirnov.

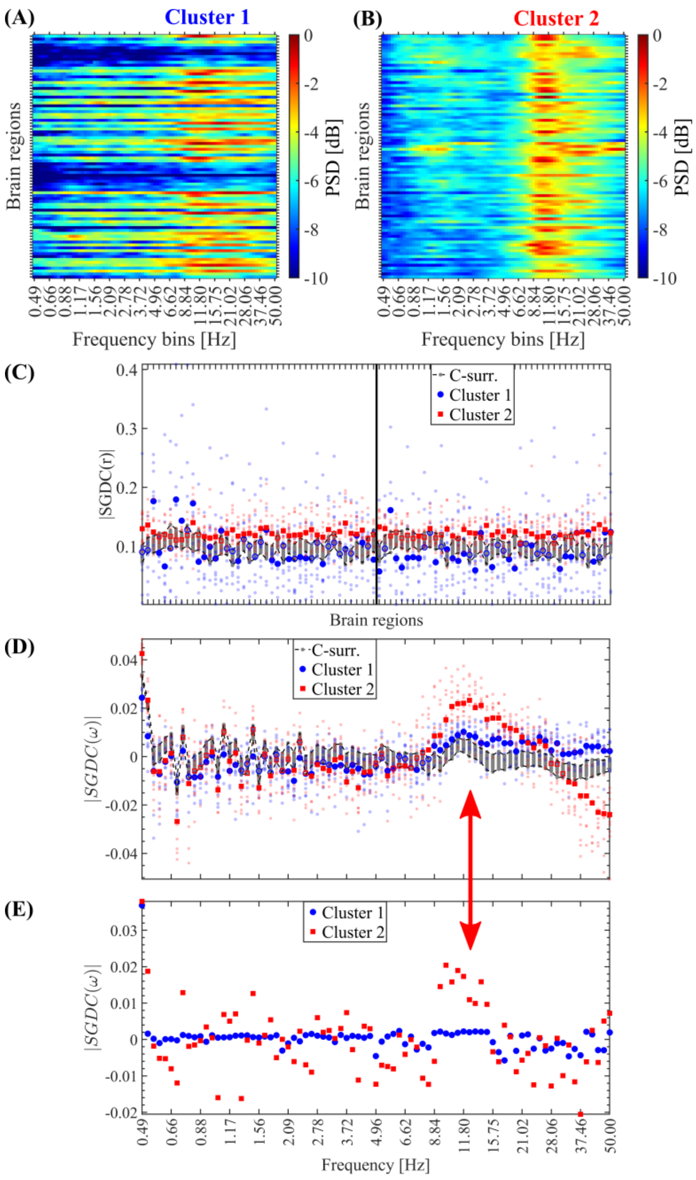

Figure C.11: Transient cross-regional coherence around the alpha band is mainly associated with salient events. (A, B) Mean ESM of the two SE clusters identified by the Louvain algorithm computed on the SEs detected in the 10 participants. (C) Transient cross-frequency coherence quantified by the $SGDC(r)$ measure (see Supplementary Material A.3), associated with the two SE clusters shown in panels A and B. The $SGDC(r)$ measure was computed in a time-resolved manner. That is, the $SGDC(r)$ measure was computed on each detected SE by considering the brain regions and time interval associated with each particular event. Then, the $SGDC(r)$ array was averaged selectively across the SEs segregated in the two clusters produced by the Louvain algorithm (see Section 2.9 in Methods). The small markers represent mean $|SGDC(r)|$ values averaged across the SEs in each individual participant. The big markers represent mean $|SGDC(r)|$ values averaged across the 10 participants. (D) Same as in C for the transient cross-regional coherence quantified by the $SGDC(\omega)$ measure (see Supplementary Material A.3). (G) Same as in D for the synthetic data corresponding to the large-scale signal model (see Section 3.2). The red arrow highlight the increase of the $|SGDC(\omega)|$ values around the alpha band. Symbols and abbreviations: SEs, Salient Events; ESM, Event Spectral Matrix.

Supplementary Material D. Supplementary empirical results excluding the deep sources

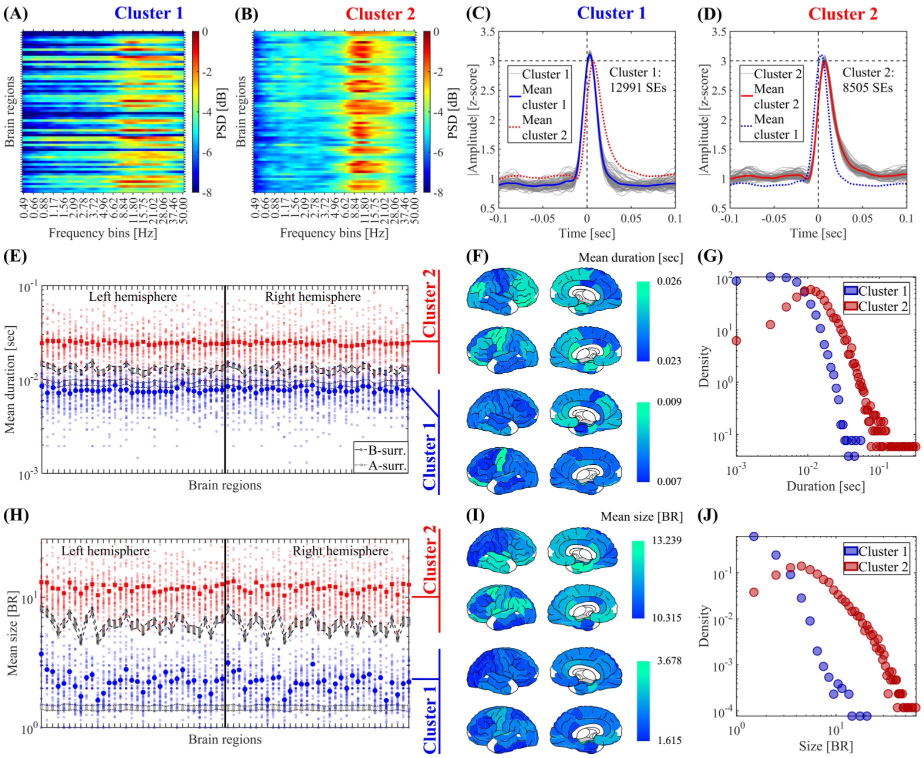

Figure D.1: Clustering of SEs according to their spectral signature. The SEs obtained from 45 subjects were clustered using the Louvain algorithm (resolution parameter $\gamma = 1$, see Methods). (A, B) Mean ESM of the two SE clusters identified by the Louvain algorithm computed on the SEs detected in the 45 participants. (C, D) Waveform shapes of the SEs pertaining to the two SE clusters identified by the Louvain algorithm. Thin gray lines correspond to the average waveform shape in each brain region. Thick blue and red lines correspond to the resulting waveform shape averaged across the brain regions for cluster 1 and 2 SEs, respectively. (E) Spatial profile showing the mean duration of SEs pertaining to cluster 1 (in blue) and cluster 2 (in red). For the true data, the small and big markers correspond to the mean spatial profile in each patient and the average across the 45 participants, respectively (see Methods). The labels and ordering of the brain regions are the same as those shown in Fig. C.2. To test the significance of the difference of the mean SEs duration between cluster 1 and cluster 2, in each brain region we computed a non-parametric permutation test (random sampling without replacement, 1×10^4 permutations). All the brain regions disclosed a statistically significant difference of the mean SEs duration between cluster 1 and 2 (the Bonferroni-adjusted two-tailed P values result $P < 0.001$ in all the brain regions). (F) Brain topographies for the mean duration of SEs averaged across the 45 participants as shown in panel E. (G) Distribution of the duration of SEs pertaining to the cluster 1 and cluster 2 observed in the 45 participants. (H) Same as in E for the size of SEs. To test the significance of the difference of the mean SEs size between cluster 1 and cluster 2, in each brain region we computed a non-parametric permutation test (random sampling without replacement, 1×10^4 permutations). All the brain regions disclosed a statistically significant difference of the mean SEs size between cluster 1 and 2 (the Bonferroni-adjusted two-tailed P values result $P < 0.001$ in all the brain regions). (I) Same as in F for the size of SEs. (J) Same as in G for the size of SEs. Symbols and abbreviations: SEs, Salient Events; ESM, Event Spectral Matrix; BR, Brain Regions.

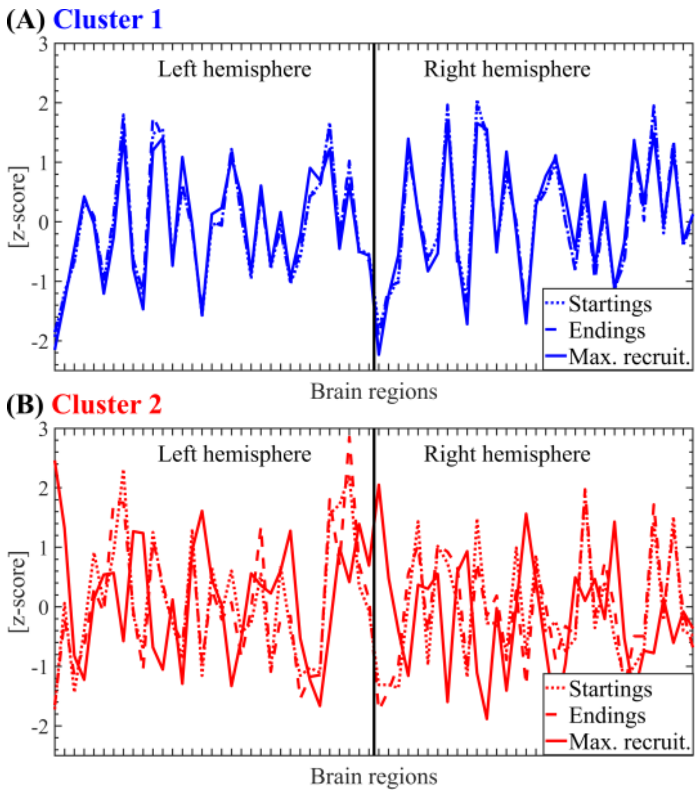

Figure D.2: Salient events propagation modes segregated by SE clusters. (A) Spatial profile for the cluster 1 SEs starting, maximum recruitment and ending modes (see Section 2.7 in Methods) computed on 45 participants. Linear correlations between topographies: Startings vs Endings, $r = 0.995$, $P < 0.001$. Max. recruit. vs Startings, $r = 0.972$, $P < 0.001$. Max. recruit. vs Endings, $r = 0.968$, $P < 0.001$. (B) Same as in A for the cluster 2 SEs starting, maximum recruitment and ending modes. Linear correlations between topographies: Startings vs Endings, $r = 0.917$, $P < 0.001$. Max. recruit. vs Startings, $r = -0.052$, $P = 0.7$. Max. recruit. vs Endings, $r = -0.051$, $P = 0.7$. The SEs obtained from 45 subjects were clustered using the Louvain algorithm (resolution parameter $\gamma = 1$, see Section 2.9 in Methods). The reported P values for the statistical significance of the Pearson's correlation were assessed using Student's t distributions of the two-tailed hypothesis test under the null hypothesis that the correlation is zero. Symbols and abbreviations: SEs, Salient Events.

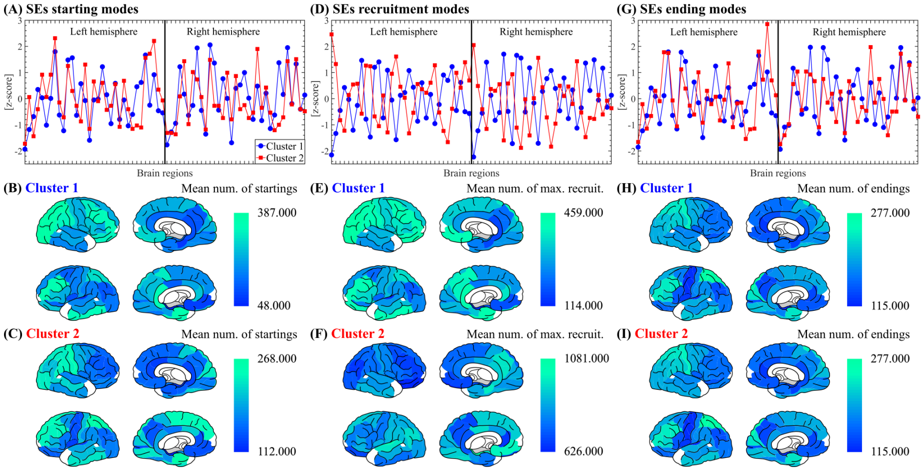

Figure D.3: Salient events propagation modes. (A) Spatial profile for the SEs starting modes (see Section 2.7 in Methods) corresponding to the two SE clusters computed on 45 participants. The SEs obtained from 45 subjects were clustered using the Louvain algorithm (resolution parameter $\gamma = 1$, see Section 2.9 in Methods). The Pearson's correlation between the spatial profiles of cluster 1 and cluster 2 SEs is $r = 0.584$, $P < 0.001$. (B) Brain topographies for the starting modes of cluster 1 SEs as shown in panel A. (C) Brain topographies for the starting modes of cluster 2 SEs as shown in panel A. (D-F) Same as A-C for SEs maximum recruitment modes (see Section 2.7 in Methods). In panel D, the Pearson's correlation between the spatial profiles of cluster 1 and cluster 2 SEs is $r = -0.842$, $P < 0.001$. (G-I) Same as A-C for SEs ending modes (see Section 2.7 in Methods). In panel G, the Pearson's correlation between the spatial profiles of cluster 1 and cluster 2 SEs is $r = 0.571$, $P < 0.001$. The reported P values for the statistical significance of the Pearson's correlation were assessed using Student's t distributions of the two-tailed hypothesis test under the null hypothesis that the correlation is zero. Symbols and abbreviations: SEs, Salient Events.
